# Child development with the D-score: turning milestones into measurement

**DOI:** 10.12688/gatesopenres.13222.1

**Published:** 2021-05-13

**Authors:** Stef van Buuren, Iris Eekhout

**Affiliations:** 1 Netherlands Organisation for Applied Scientific Research TNO, Leiden, 2316 ZL, The Netherlands; 2 University of Utrecht, Utrecht, 3584 CH, The Netherlands

**Keywords:** child development, Rasch model, growth chart, D-score, item difficulty, developmental milestones, Dutch Development Instrument (Van Wiechenschema) measurement, D-score standard deviation score (DAZ), early intervention, children 0-2 years, preterm

## Abstract

The chapter equips the reader with a basic understanding of robust psychometric methods that are needed to turn developmental milestones into measurements, introducing the fundamental issues in defining a unit for child development and demonstrates the relevant quantitative methodology. It reviews quantitative approaches to measuring child development; introduces the Rasch model in a non-technical way; shows how to estimate model parameters from real data; puts forth a set of principles for model evaluation and assessment of scale quality; analyses the relation between early D-scores and later intelligence; and compares the D-scores from three studies that all use the same instrument.

## 1 Introduction

This introductory section outlines why we utilize the D-score:

•    reviewing key discussions about the first 1000 days in a child’s life (1.1)

•    highlighting the relevance of early childhood development for later life (1.2)

•    discussing the use of stunting as a proxy for development (1.3)

•    pointing to existing instruments to quantify neurocognitive development (1.4)

•    explaining why we have written this chapter (1.5)

•    delineating the intended audience (1.6)

### 1.1 First 1000 days

The *first 1000 days* refers to the time needed for a child to grow from conception to the second birthday. It is a time of rapid change. During this period the architecture of the developing brain is very open to the influence of relationships and experiences ( Shonkhoff *et al.*, 2016). Early experiences affect the nature and quality of the brain’s developing architecture by reinforcing some synapses and pruning others through lack of use. The first 1000 days shape the brain’s architecture, but higher-order brain functions continue to develop into adolescence and early adulthood ( Kolb *et al.*, 2017).

The classic *nature versus nurture debate* contrasts the viewpoints that variation in development is primarily due to either genetic or environmental differences. The current scientific consensus is that both genetic predisposition and ecological differences influence all traits ( Rutter, 2007). The environment in which a child develops (before and soon after birth) provides experiences that can modify gene activity ( Caspi *et al.*, 2010). Negative influences, such as exposure to stressful life circumstances or environmental toxins may leave a *chemical signature* on the genes, thereby influencing how genes work in that individual.

**Figure 1.1.**
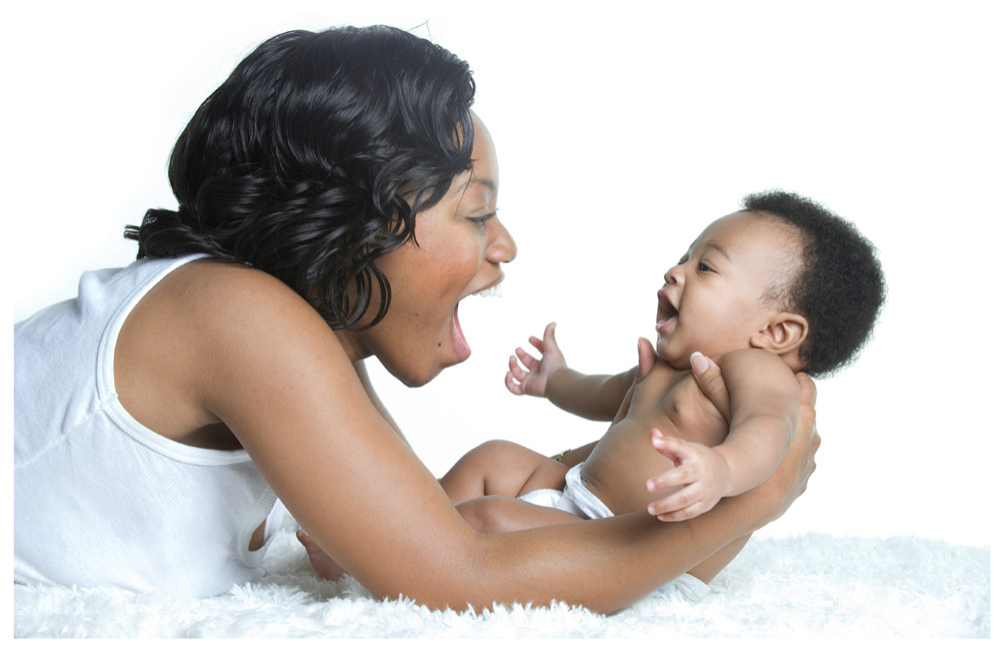
Serve and return interactions shape brain architecture. Source: Shutterstock, under license.

During the first 1000 days, infants are highly dependent on their caregivers to protect them from adversities and to help them regulate their physiology and behavior. As Figure 1.1 illustrates, caregivers can do this through responsive care, including routines for sleeping and feeding. To reach their developmental potential, children require nutrition, responsive caregiving, opportunities to explore and learn, and protection from environmental threats ( Black *et al.*, 2017). Gradually, children build self-regulatory skills that enable them to manage stress as they interact with the world around them ( Johnson *et al.*, 2013).

### 1.2 Relevance of child development

The first 1000 days is a time of rapid change. Early experiences affect brain development and influence lifelong learning and health ( Shonkhoff *et al.*, 2016). Healthy development is associated with future school achievement, well-being, and success in life ( Bellman *et al.*, 2013).

Professionals and parents consider it important to monitor children’s development. Tracking child development enables professionals to identify children with signs of potential delay. Timely identification can help children and their parents to benefit from early intervention. In a normal population, developmental delay affects about 1–3% of children. A delay in development may indicate underlying disorders. About 1% of children have an autism spectrum disorder ( Baird *et al.*, 2006), 1–2% a mild learning disability, and 5–10% have a specific learning disability in a single domain ( Horridge, 2011).

Children develop at different rates, and it is vital to distinguish those who are within the “normal” range from those who are following a more pathological course ( Bellman *et al.*, 2013). There is good evidence that early identification and early intervention improve the outcomes of children ( Britto *et al.*, 2017). Early intervention is crucial for children with developmental disabilities because barriers to healthy development early in life impede progress at each subsequent stage.

Monitoring child development provides caregivers and parents with reliable information about the child and an opportunity to intervene at an early age. Understanding the developmental health of populations of children allows organisations and policymakers to make informed decisions about programmes that support children’s greatest needs ( Bellman *et al.*, 2013).

### 1.3 Stunting as proxy for child development

Stunting is the impaired physical growth and development that children experience from poor nutrition, repeated infection, and inadequate psychosocial stimulation. Linear growth in children is commonly expressed as length-for-age or height-for-age in comparison to normative growth standards ( Wit *et al.*, 2017). According to the World Health Organization (WHO), children are stunted if their height-for-age is more than two standard deviations below the Child Growth Standards median. Stunting caused by chronic nutritional deprivation in early childhood is as an indicator of child development ( Perkins *et al.*, 2017).

There is consistent evidence for an association between stunting and poor child development, despite heterogeneity in the estimation of its magnitude ( Miller *et al.*, 2016; Sudfeld *et al.*, 2015). Considering impaired linear growth as a proxy measure for child development is easy to do, and quite common. Yet, using impaired height growth as a measure for child development is not without limitations:

•    The relation between height and child development is weak after adjustment for age;

•    Height is a physical indicator that does not take into account a direct evaluation of a child’s cognitive or mental performance;

•    There is considerable heterogeneity in heights of children all over the world;

•    Height is not sensitive to rapid changes in child development.

### 1.4 Measuring neurocognitive development

Assessment of early neurocognitive development in children is challenging for many reasons ( Ellingsen, 2016). During the first years of life, developmental change occurs rapidly, and the manifestation of different skills and abilities varies considerably across children. Moreover, a child’s performance on a cognitive task is very susceptible to measurement setting, timing and the health of the child that day.

Recently, a toolkit was published that reviews 147 assessment tools developed for children ages 0–8 years in low- and middle-income countries ( Fernald *et al.*, 2017). Some of the most widely used tools include the Ages & Stages Questionnaires (ASQ), Achenbach Child Behavior Checklist (CBCL), Bayley Scales of Infant Development (BSID), Denver Developmental Screening Test (DEN), Griffiths Scales of Child Development (GRF), Mullen Scale of Early Learning (MSEL), Strengths and Difficulties Questionnaire (SDQ), Wechsler Intelligence Scale for Children (WISC), and its younger age counterpart Wechsler Preschool and Primary Scale of Intelligence (WPPSI).

Each of these tools has its strengths and limitations. For example, the ASQ and DEN are screeners for general child development. The CBCL and SDQ are screeners for behavioral and mental health, not cognition or general development. DEN is relatively easy and quick to administer, but not very precise. It is out of production, not being sold or re-normed. The BSID, MSEL, and GRF provide a clinical assessment at the individual level and requires a skilled professional to administer. Some instruments collect observations through the caregiver (ASQ), whereas others emphasize traits and behavior over performance (SDQ, CBCL). Also, the age ranges to which the instruments are sensitive vary. Furthermore, they may cover different domains of development.

The ideal child development assessment would be easy to administer and has high reliability, validity, and cross-cultural appropriateness. It should also show appropriate sensitivity in scores at different ages and ability levels. It is no surprise that no test can meet all of these criteria. Many tests are too long, difficult to administer, lack cross-cultural validity, or have low reliability. Also, many instruments are proprietary and costly to use.

### 1.5 Why this chapter?

We believe that **there cannot be one instrument** for measuring child development that is suitable for all situations. In general, the tool needs tailoring to the setting. For example, to find a delayed child, we need an instrument that is precise for that individual child, and that is sensitive to different domains of delay. In contrast, if we want to estimate the proportion of children that is *developmentally on track* in a region, we need one culturally unbiased, relatively imprecise low-cost measurement made on many children across many ages. The optimal instrument will look quite different in both cases.

We also believe that **there can be one scale** for measuring child development and that this scale is useful for many applications. Such a scale is similar to well-known measures for body height, body weight or body temperature. These measurements have a clearly defined unit (i.e., centimetre, kilogram, degree Celsius), which moreover is assumed to be constant across all scale locations. We express measurements as the number of scale units (e.g. 92 cm). Note that there may be multiple instruments for measuring a child height (e.g. ruler, laser distance meter, echolocation, ability to reach the door handle, and so on). Still, their result translates into scale units (cm here). The opposite is also true, and perhaps more familiar. We may have one instrument and express the result in multiple units (e.g. cm, inches, light-years).

Instruments and scales are different things. Currently, instruments for measuring child development define their own scales, which renders the measurements made by distinct tools incomparable. No measurement unit for child development yet exists. It would undoubtedly be an advance if we could tailor the measurement instrument to the setting while retaining the advantage of a scale with a clearly defined unit across different tools. We can then compare the data collected by distinct devices. This chapter explores the theory and practice for making that happen.

### 1.6 Intended audience

We aim for three broad audiences:

•    Professionals in the field of child growth and development;

•    Policymakers in international settings;

•    Statisticians, methodologists, and data scientists.

Professionals in child development will become familiar with a new approach to measuring child development in early childhood. We plan to separate the measurement instrument from the scale used to express the result. This formulation allows the user **to select the instrument most suited for a particular setting**. Since instruments differ widely in age coverage, length, administration mode, and domain coverage ( Boggs *et al.*, 2019), the ability to choose the instrument, while not giving up comparability, represents a significant advance over routines that marry the scale to the instrument.

Policymakers in international settings wish to know the effect of different interventions on child development. Gaining insight into such effects is not so easy since different studies use different instruments. The ability to place measurements made by different instruments onto the same scale will allow for a **more accurate understanding of policy effects**. It also enables the setting of priorities and actions that are less dependent on the way the data were collected.

Statisticians and data scientists generally prefer numeric values with an unambiguous unit (e.g., centimeters, kilograms) over a vector of dichotomous data points. This chapter shows how to convert a series of PASS/FAIL scores to a numeric value with interval scale properties. The existence of such a scale opens the way for the **application of precise analytic techniques**, similar to those applied to child height and body weight. The techniques have a solid psychometric backing, and also apply to other types of problems.

## 2 Short history

The measurement of child development has quite an extensive history. This section

•    reviews definitions of child development (2.1)

•    discusses concepts in the nature of child development (2.2)

•    shows a classic example of motor measurements (2.3)

•    summarizes typical questions whose answers need proper measurements (2.4)

### 2.1 What is child development?

In contrast to concepts like height or temperature, it is unclear what exactly constitutes child development. Shirley (1931) executed one of the first rigorous studies in the field with the explicit aim

        that the many aspects of development, anatomical, physical, motor, intellectual, and emotional, be studied simultaneously.

Shirley gave empirical definitions of each of these domains of development.

**Figure 2.1.**
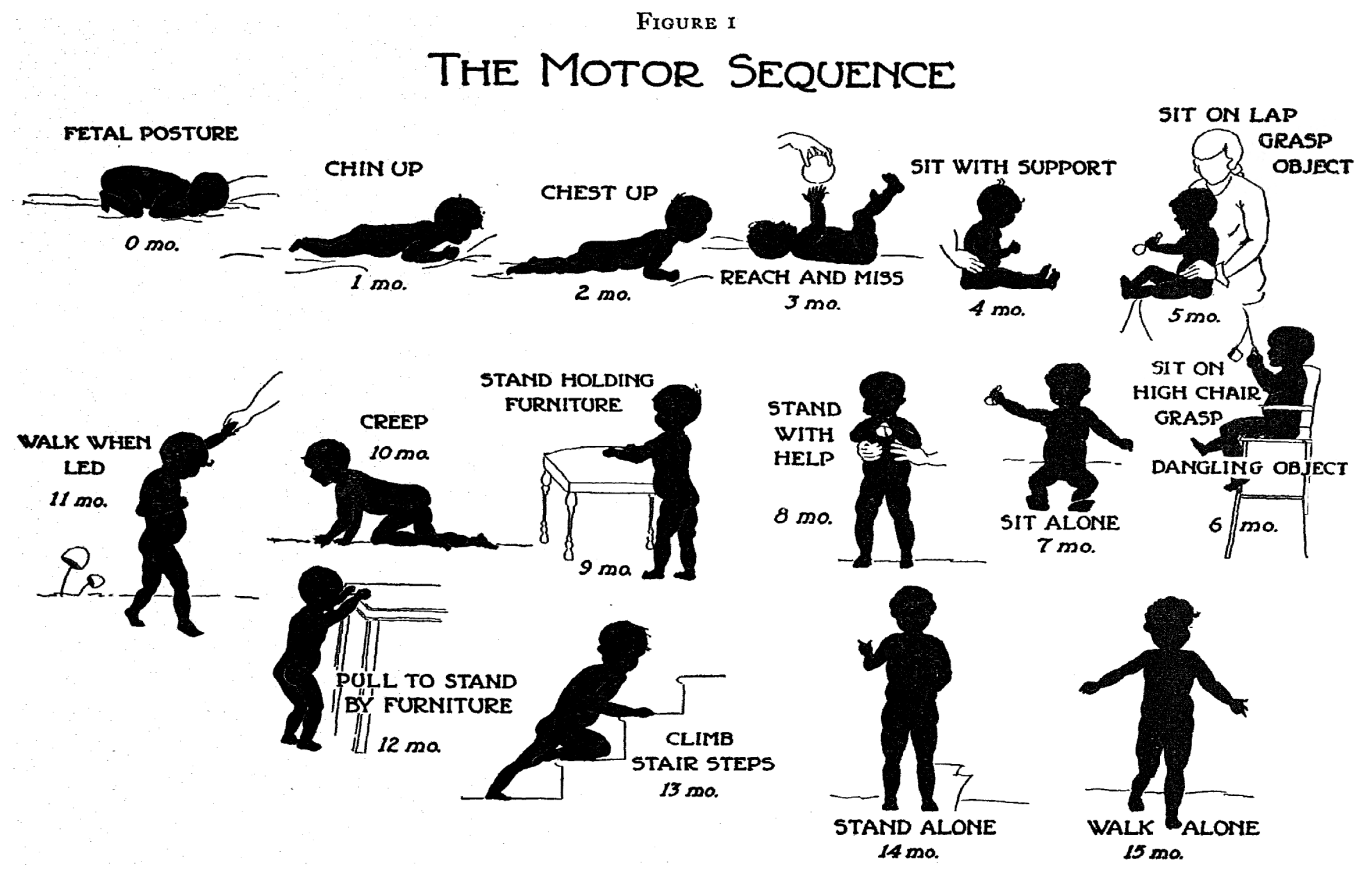
Gross motor development as a sequence of milestones. Source: Shirley (1933), with permission.

Certain domains advance through a fixed sequence. Figure 2.1 illustrates the various stages needed for going from a *fetal posture* to *walking alone*. The ages are indicative of when these events happen, but there is a considerable variation in timing between infants.

Gesell (1943) (p. 88) formulated the following definition of development:

        Development is a continuous process that proceeds stage by stage in an orderly sequence.

Gesell’s definition emphasizes that development is a continuous process. The stages are useful as indicators to infer the level of maturity but are of limited interest by themselves.

Liebert *et al.* (1974) (p. 5) emphasized that development is not a phenomenon that unfolds in isolation.

        Development refers to a process in growth and capability over time, as a function of both maturation and interaction with the environment.

Cameron & Bogin (2012) (p. 11) defined an endpoint of development, as follows:

        “Growth” is defined as an increase in size, while “maturity” or “development” is an increase in functional ability…The endpoint of maturity is when a human is functionally able to procreate successfully … not just biological maturity but also behavioural and perhaps social maturity.

Berk (2011) (p. 30) presented a dynamic systems perspective on child development as follows:

        Development cannot be characterized as a single line of change, and is more like a web of fibres branching out in many directions, each representing a different skill area that may undergo both continuous and stagewise transformation.

There are many more definitions of child development. The ones described here illustrate the main points of view in the field.

### 2.2 Theories of child development

The field of child development is vast and spans multiple academic disciplines. This short overview, therefore, cannot do justice to the enormous richness. Readers new to the field might orient themselves by browsing through an introductory academic titles ( Berk, 2011; Santrock, 2011), or by searching for the topic of interest in an encyclopedia, e.g., Salkind (2002).

The introductions by Santrock (2011) and Berk (2011) both distinguish major theories in child development according to how each answer to following three questions:

***2.2.1 Continuous or discontinuous?*** Does development evolve gradually as a continuous process or are there qualitatively distinct stages, with jumps occurring from one step to another?

Many stage-based theories of human development have been proposed over the years: social and emotional development by psycho-sexual stages introduced by Freud and furthered by Erikson ( Erikson, 1963), Kohlberg’s six stages of moral development ( Kohlberg, 1984) and Piaget’s cognitive development theory ( Piaget & Inhelder, 1969). Piaget distinguishes four main periods throughout childhood. The first period, the *sensorimotor period* (approximately 0–2 years), is subdivided into six stages. When taken together, these six stages describe “the road to conceptual thought.” Piaget’s stages are qualitatively different and aim to unravel the mechanism involved in intellectual development.

On the other hand, Gesell and others emphasize development as a continuous process. Gesell (1943) (p. 88) says:

        A stage represents a degree or level of maturity in the cycle of development. A stage is simply a passing moment, while development, like time, keeps marching on.

***2.2.2 One course or multiple parallel tracks?*** Stage theorists assume that children progress sequentially through the same set of stages. This assumption is also explicit in the work of Gesell.

The ecological and dynamic systems theories view development as continuous, though not necessarily progressing in an orderly fashion, so there may be multiple, parallel ways to reach the same point. The developmental path taken by a given child will depend on the child’s unique combination of personal and environmental circumstances, including cultural diversity in development.

**Figure 2.2.**
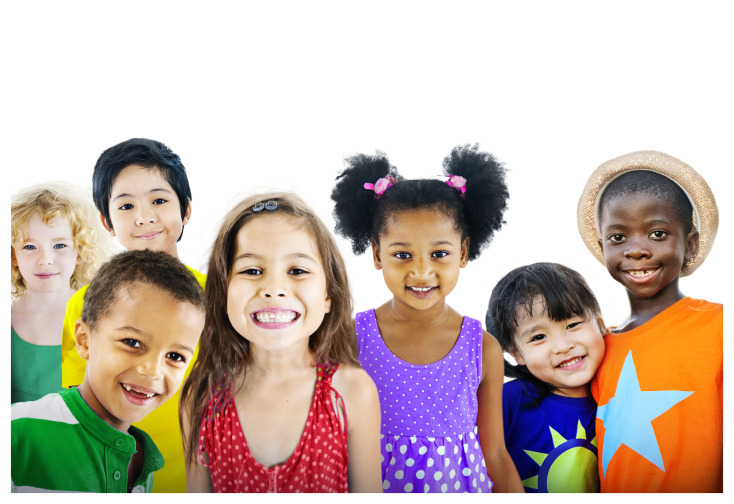
A group of culturally diverse children. Source: Shutterstock, under license.

***2.2.3 Nature or nurture?*** Figure 2.2 illustrates that children vary in appearance. Are genetic or environmental factors more important for influencing development? Most theories generally acknowledge the role of both but differ in emphasis. In practice, the debate centres on the question of how to explain individual differences.

Maturation is the process of becoming fully developed, much like the natural unfolding of a flower. The process depends on both genetic factors (species, breed) as well as environmental influences (sunlight, water, nutrition). Some theorists emphasize that differences in child development are innate and stable over time, although there may be differences in unfolding speed due to different environments. Others argue that environmental factors drive differences in development between children, and changing these factors could very well impact child development.

Our position in this debate has practical implications. If we believe that differences are natural and stable, then it may not make much sense trying to change the environment, as the impact on development is likely to be small. On the other hand, we may consider developmental potential as evenly distributed, with its expression governed by the environment. In the latter case, improving life circumstances may have substantial pay-offs in terms of better development.

### 2.3 Example of motor development

***2.3.1 Shirley’s motor data*.** For illustration, we use data on loco-motor development from a classic study on child development among 25 babies. Shirley (1931) collected measurements of the baby’s walking ability, starting at ages around 13 weeks, in an ingenious way. The investigator lays out a white paper of twelve inches wide on the floor of the living room, and lightly greases the soles of the baby’s feet with olive oil. The baby was invited to “walk” on the sheet. Of course, very young infants need substantial assistance. Footprints left were later coloured by graphite and measured. Measurements during the first year were repeated every week or bi-weekly.

Table 2.1 ( Shirley, 1931, Appendix 8) lists the age (in weeks) of the 21 babies when they started, respectively, stepping, standing, walking with help, and walking alone. Blanks indicate missing data. A blank in the first column means that the baby was already stepping when the observation started (Virginia Ruth, Sibyl, Donovan, Torey and Doris). Max and Martin, who have blanks in the second column, skipped standing and went directly from stepping to walking with help. Doris has a blank in the last column because she passed away before she could walk alone.

**Table 2.1.** Age at beginning stages of walking (in weeks) for 21 babies. Source: Shirley (1931).

Name	Sex	Stepping	Standing	Walking with help	Walking alone
Martin	boy	15		21	50
Carol	girl	15	19	37	50
Max	boy	14		25	54
Virginia Ruth	girl		21	41	54
Sibyl	girl		22	37	58
David	boy	19	27	34	60
James D.	boy	19	30	45	60
Harvey	boy	14	27	42	62
Winnifred	girl	15	30	41	62
Quentin	boy	15	23	38	64
Maurice	boy	18	23	45	66
Judy	girl	18	29	45	66
Irene May	girl	19	34	45	66
Peter	boy	15	29	49	66
Walley	boy	18	33	54	68
Fred	boy	15	32	46	70
Donovan	boy		23	50	70
Patricia	girl	15	30	45	70
Torey	boy		21	72	74
Larry	boy	13	41	54	76
Doris	girl		23	44	

**Figure 2.3.**
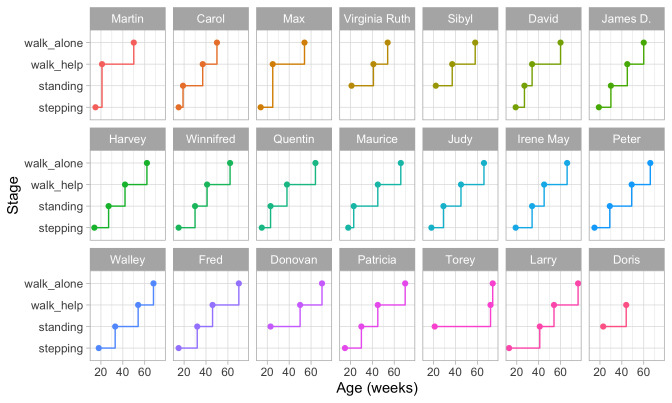
Staircase plot indicating the age at which each baby achieves a new milestone of gross-motor functioning.

***2.3.2 Individual trajectories of motor development.*** Figure 2.3 is a visual representation of the information in Table 2.1. Each data point is the age of the first occurrence of the next stage. Before that age, we assume the baby is in the previous stage.

Figure 2.3 makes it easy to spot the quick walkers (Martin, Carol) and slow walkers (Patricia, Torey, Larry). Furthermore, we may also locate children who remain a long time in a particular stage (Torey, Larry) or who jump over stages (Martin, Max).

For ease of plotting, the categories on the vertical axis are equally spaced. The height of the jump from one stage to the next has no sensible interpretation. We might be inclined to think that the vertical distance portrays to how difficult it is to achieve the next stage, but this is inaccurate. Instead, the ability needed to set the next step corresponds to the *horizontal line length* between stages. For example, on average, the line for stepping is rather short in all plots, so going from stepping to standing is relatively easy.

Figure 2.3 presents data from only those visits where a jump occurred. The number of house visits made during the ages of 0–2 years was far higher. Shirley (1931) collected data from 1370 visits, whereas Figure 2.3 plot only the 76 occasions that showed a jump. Thus the data collection needs to be intense and costly to obtain individual curves. Fortunately, there are alternatives that are much more efficient.

### 2.4 Typical questions asked in child development

The emotional, social and physical development of the young child has a direct effect on the adult he or she will become. We may be interested in measuring child development for answering clinical, policy or public health questions.

Table 2.2 lists typical questions whose answers require measuring child development. Note that all questions compare the amount of child development between groups or time points. A few questions compare development for the same child, group or population at different ages. Others compare development at the same age across different children, groups or populations.

**Table 2.2.** Questions whose answers require quantitative measurements of child development.

Level	Question
Individual	What is the child's gain in development since the last visit?
Individual	What is the difference in development between the child and peers of the same age?
Individual	How does the child's development compare to a norm?
Group	What is the effect of this intervention on child development?
Group	What is the difference in child development between these two groups?
Population	What is the change in average child development since the last measurement?
Population	What was the effect of implementing this policy on child development?
Population	How does this country compare to other countries in terms of child development?

## 3 Quantifying child development

This section discusses four principles to quantify child development:

•    Age-based measurement (3.1)

•    Probability-based measurement (3.2)

•    Score-based measurement (3.3)

•    Unit-based measurement (3.4)

### 3.1 Age-based measurement of development

***3.1.1 Motivation for age-based measurement.*** Milestones form the based building blocks for instruments to measure child development. Methods to quantify growth using separate milestones relate the milestone behaviour to the child’s age. Gesell (1943) (p. 89) formulated this goal as follows:

        We think of behaviour in terms of age, and we think of age in terms of behaviour. For any selected age it is possible to sketch a portrait which delineates the behaviour characteristics typical of the age.

There is an extensive literature that quantifies development in terms of the ages at which the child is expected to show a specific behaviour. The oldest methods for quantifying child development calculate an *age equivalent* for achieving a milestone, and compare the child’s age to this age equivalent.

**Figure 3.1.**
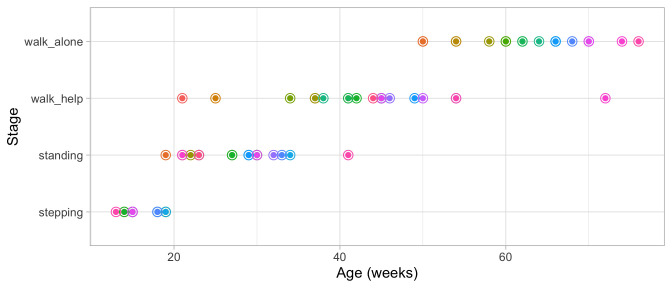
Ages at which 21 children achieve four motor development milestones.

***3.1.2 Age equivalent and developmental age.*** Figure 3.1 graphs the ages at which each of the 21 children enter a given stage in Shirley’s motor data of Table 2.1. Since standing follows stepping, children who can stand are older than the children who are stepping. Hence the ages for standing are located more to the right.

Since age and development are so intimately related, we can express the *difficulty* of a milestone as the *mean age* at which children achieve it. For example, Stott (1967) (p. 25) defines the *age equivalent* and its use for measurement, as follows:

        The age equivalent of a particular stage is simply the average age at which children reach that particular stage.

**Figure 3.2.**
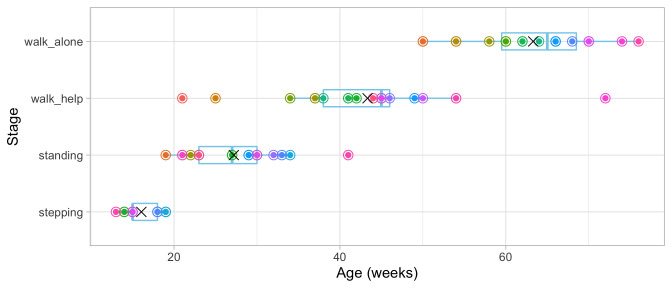
Mean (symbol x) and spread of the ages at which 21 children achieve four motor development milestones.

Figure 3.2 adds the mean age and the boxplot at which the children enter the four stages. The difficulty of these milestones can thus be expressed as age equivalents: 16.1 weeks for stepping, 27.2 weeks for standing, 43.3 weeks for walking with help and 63.3 weeks for walking alone.

Thus, a child that is stepping beyond the age of 16.1 weeks is considered later than average, whereas a child already stepping before 27.2 weeks earlier than average. We may also calculate age delta as the difference between the child’s age and the norm age, and express it as “two weeks late” or “three weeks ahead.” Summarizing age delta’s over different milestones has led to concepts like *developmental age* as a measure of a child’s development.

***3.1.3 Limitations of age-based measurement.*** Age-based measurement is easy to understand, and widely used in the popular press, but not without pitfalls:

1.   Age-based measurement requires us to know the ages at which the child entered a new stage. The mean age can be a biased estimate of item difficulty if visits are widely apart, irregular or missing.

2.   Age-based measurement can inform us whether a child is achieving a given milestone early of late. However, it does not tell us what behaviours are characteristic for children of a given age.

3.   Age-based measurement cannot exist without an age norm. When there are no norms, we cannot quantify development.

4.   Age-based measurement works only at the item level. Although we may average age delta’s over milestones, the choice of milestones is arbitrary.

### 3.2 Probability-based measurement

An alternative is to calculate the *probability* of achieving a milestone at a given age and compare the child’s response to that probability.

The passing probability is an interpretable and relevant measure. An operational advantage of the approach is that the necessary calculations place fewer demands on the available data and can be done even for cross-sectional studies.

**Figure 3.3.**
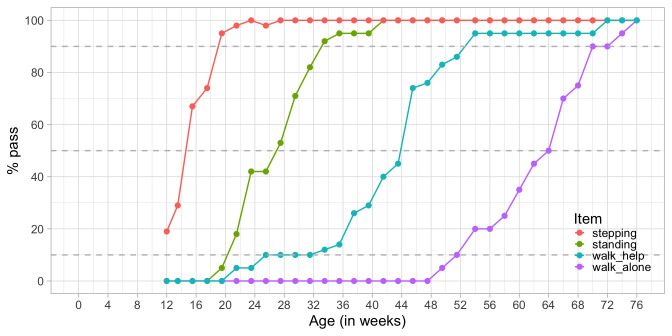
Probability of achieving four motor milestones against age.

***3.2.1 Example of probability-based measurement.*** Figure 3.3 plots the percentage of children achieving each of Shirley’s motor stages against age. There are four cumulative curves, one for each milestone, that indicate the percentage of children that pass.

In analogy to the age equivalent introduced in Section 3.1.2 we can define the *difficulty* of the milestone as the age at which 50 per cent of the children pass. In the Figure we see that the levels of difficulty are approximately 14.2 weeks ( stepping), 27.0 weeks ( standing), 43.8 weeks ( walking with help) and 64.0 weeks ( walking alone). Also, we may easily find the ages at which 10 per cent or 90 per cent of the children pass each milestone.

Observe there is a gradual decline in the steepness as we move from stepping to walk_alone. For example, we need an age interval of 13 weeks (33 - 20) to go from 10 to 90 per cent in standing, but need 19 weeks (71 - 52) to go from 10 to 90 per cent in walking alone. Thus, one step on the age axis corresponds to different increments in probability. The flattening pattern is typical for child development and represents evidence that evolution is faster at earlier ages.

***3.2.2 Limitations of probability-based measurement.*** Probability-based measurement is a popular way to create instruments for screening on developmental delay. For example, each milestone in the Denver II ( Frankenburg *et al.*, 1992) has markers for the 25th, 50th, 75th and 90th age percentile.

1.   The same age step corresponds to different probabilities.

2.   The measurement cannot exist without some norm population. When norms differ, we cannot compare the measurements.

3.   Interpretation is at the milestone level, sometimes supplemented by procedures for counting the number of delays. No aggregate takes all responses into account.

### 3.3 Score-based measurement of development

***3.3.1 Motivation for score-based measurement.*** Score-based measurement takes the responses on multiple milestones and counts the total number of items passed as a measure of development. This approach takes all answers into account, hence leading to a more stable result.

One may order milestones in difficulty, and skip those that are too easy, and stop administration for those that are too difficult. In such cases, we cannot merely interpret the sum score of a measure of development. Instead, we need to correct for the subset of administered milestones. The usual working assumption is that the child would have passed all easier milestones and failed on all more difficult ones. We may repeat this procedure for different domains, e.g. motor, cognitive, and so on.

**Figure 3.4.**
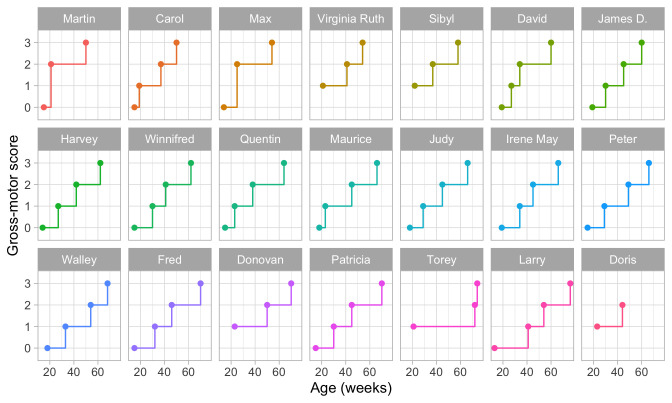
Same data as in Figure 2.3, but now with the vertical axis representing gross-motor score.

***3.3.2 Example of score-based measurement.*** Figure 3.4 is a gross-motor score calculated as the number of milestones passed. It varies from 0 to 3.

The plot suggests that the difference in development between scores 0 and 1 is the same as the difference between, say, scores 2 and 3. *This is not correct*. For example, suppose that we express the difficulty of the milestone as an age-equivalent. From Section 3.1.2 we see that the difference between stepping and standing is 27.2 - 16.1 = 11.1 weeks, whereas the difference between walking alone and walking with help is 63.3 - 43.3 = 20 weeks. Thus, according to age equivalents scores 0 and 1 should be closer to each other, and ratings 2 and 3 should be drawn more apart.

***3.3.3 Limitations of score-based measurement.*** Score-based measurement is today’s dominant approach, but is not without conceptual and logistical issues.

1.   The total score depends not only on the actual developmental status of the child, but also on the set of milestones administered. If a milestone is skipped or added, the sum score cannot be interpreted anymore as a measure of developmental status. It might be possible to correct for starting and stopping rules under the assumptions described in Section 3.3.1, but such will be involved if intermediate milestones are missing.

2.  It is not possible to compare the scores made by different instruments. Some instruments allow conversion to age-conditional scores. However, the sample used to derive such transformations pertain to that tool and does not generalise to others.

3.   Domains are hard to separate. For example, some cognitive milestones tap into fine motor capabilities, and vice versa. There are different ways to define domains, so domain interpretation varies by instrument.

4.   Administration of a full test may take substantial time. The materials are often proprietary and costly.

### 3.4 Unit-based measurement of development

***3.4.1 Motivation for unit-based measurement.*** Unit-based measurement starts by defining ideal properties and derives a procedure to aggregate the responses on milestones into an overall score that will meet this ideal.

Section 2.4 highlighted questions for individuals, groups and populations. There are three questions:

•    What is the difference in development over time for the same child, group or community?

•    What is the difference in development between different children, groups or populations of the same age?

•    How does child development compare to a norm?

In the ideal situation, we would like to have a continuous (latent) variable *D* (for development) that measures child development. The scale should allow us to quantify *ability* of persons, groups or populations from low to high. It should have a *constant unit* so that a given difference in ability refers to the same quantity across the entire scale. We find the same property in height, where a distance of 10 cm represents the same amount for molecules, people or galaxies. When are these conditions are met, we say that we measure on an *interval scale*.

If we succeed in creating an interval scale for child development, an enormous arsenal of techniques developed for quantitative variables opens up to measure, track and analyze child development. We may then evaluate the status of a child in terms of *D* points gained, create age-dependent diagrams (just like growth charts for height and weight), devise age-conditional measures for child development, and intelligent adaptive testing schemes. Promising studies on Dutch data van Buuren (2014) suggest that such benefits are well within reach.

**Figure 3.5.**
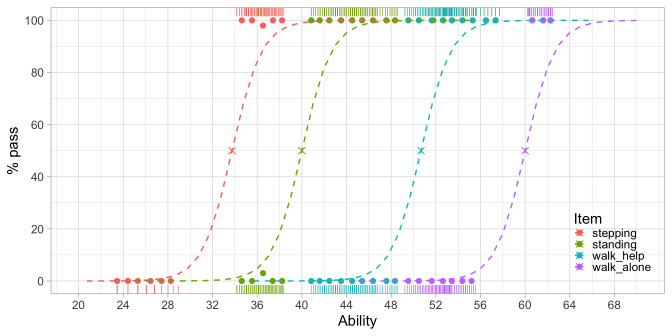
Modeled probability of achieving four motor milestones against the D-score.

***3.4.2 Example of unit-based measurement.*** Figure 3.5 is similar to Figure 3.3, but with Age replaced by Ability. Also, modelled curves have replaced empirical ones, but this is not essential.

We estimated the ability values on the horizontal axis from the data. The values correspond to the amount of development of each visit. Likewise, we calculated the logistic curves from the data. These reflect the probability of passing each milestone *at a given level of ability*.

Figure 3.5 shows that the probability of passing a milestone increases with ability. Items are sorted according to difficulty from left to right. Milestone stepping is the easiest and walk_alone is the most difficult. The point at which a logistic curve crosses the 50 per cent line (marked by a cross) is the *difficulty of the milestone*.

The increase in ability that is needed to go from 10 to 90 per cent is about five units here. Since all curves are parallel, the interval is constant for all scale locations. Thus, the scale is an *interval scale* with a *constant unit of measurement*, the type of measurement needed for answering the basic questions identified in Section 3.4.1.

***3.4.3 Limitations of unit-based measurement.*** While unit-based measurement has many advantages, it cannot perform miracles.

1.   An important assumption is that the milestones “measure the same thing,” or put differently, are manifestations of a continuous latent variable that can be measured by empirical observations. Unit-based measurement won’t work if there is no sensible latent scale.

2.   The portrayed advantages hold only if the discrepancies between the data and the model are relatively small. Since the simplest and most powerful measurement models are strict, it is essential to obtain a good fit between the data and the model.

3.   The construction of unit-based measurement requires psychometric expertise, specialized computer software and considerable sample sizes.

### 3.5 A unified framework

This section brings together the four approaches outlined in this section into a unified framework.

**Figure 3.6.**
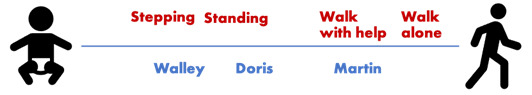
Placing milestones and children onto the same line reveals their positions.

Figure 3.6 shows the imaginary positions on a gross-motor continuum of three babies from Figure 2.1 at the age of 30 weeks. Both milestones and children are ordered along the same continuum. Thus, standing is more difficult than stepping, and at week 30, Doris is ahead of Walley in terms of motor development.

More generally, measurement is the process of locating milestones and children on a line. This line represents a *latent variable*, a continuous construct that defines the different poles of the concept that we want to measure. A latent variable ranges from low to high.

The first part of measurement is to determine the location of the milestones on the latent variable. In many cases, the instrument maker has already done that. For example, each length marker on a ruler corresponds to a milestone for measuring length. The manufacturer of the ruler has already placed the marks at the appropriate places on the tool, and we take for granted that each marker has been calibrated correctly.

A milestone for child development is similar to a length marker, but

•    we may not know how much development the milestone measures, so its location on the line is unknown, or uncertain;

•    we may not know whether the milestone measures child development at all so that it may have no location on the line.

The second part of measurement is to find the location of each child on the line. For child height, this is easy: We place the horizontal headpiece on top of the child’s head and read off the closest height marker. Since we lack a physical ruler for development, we must deduce the child’s location on the line from the responses on a series of well-chosen milestones.

By definition, we cannot observe the values of a latent variable directly. However, we may be able to measure variables (milestones) that are related to the latent variable. For example, we may have scores on tasks like *standing* or *walking with help*.

The *measurement model* specifies the relations between the actual measurements and the latent variable. Under a given measurement model, we may estimate the locations of milestones and children on the line. Section 4.5 discusses measurement models in more detail.

### 3.6 Why unit-based measurement

This section distinguished four approaches to measure child development: *age-based*, *probability-based*, *score-based* and *unit-based* measurement. Table 3.1 summarizes how the approaches evaluate on nine criteria.

**Table 3.1.** Evaluation of four measurement approaches on seven criteria.

Criterion	Age	Probability	Score	Unit
Independent of age norm	No	No	Yes	Yes
Supports multiple milestones	No	No	Yes	Yes
Latent variable	No	No	Yes	Yes
Robust to milestone skipping	Yes	Yes	No	Yes
Comparable scores	Yes	Yes	No	Yes
Probability model	No	Yes	No	Yes
Defines measurement unit	No	No	No	Yes

*Age-based measurement* expresses development in age equivalents, whose precise definition depends on the reference population. Age-based measurement does not support multiple milestones and does not use the concept of a latent variable.

*Probability-based measurement* expresses development as age percentiles for a reference population. It is useful for individual milestones but does not support multiple items or a latent variable interpretation.

*Score-based measurement* quantifies development by summing the number of passes. Different instruments make different selections of milestones, so the scores taken are unique to the tool. Thus comparing the measurement obtained by different devices is difficult. Skipping or adding items require corrections.

*Unit-based measurement* defines a unit by a theoretical model. When the data fit the model, we are able to construct instruments that produce values in a standard metric.

## 4 The D-score

Section 2 provided historical background on the nature of child development. Section 3 discussed three general quantification approaches. This section explains how to apply the unit-based approach to arrive at the D-score scale. The text illustrates the process with real data.

•    Dutch Development Instrument (DDI) (4.1)

•    Milestone passing by age and by D-score (4.2, 4.3)

•    How do age and D-score relate? (4.4)

•    Role of the measurement model (4.5)

•    Item and person response functions (4.6)

•    Engelhard invariance criteria (4.7)

•    Why the Rasch model? (4.8)

### 4.1 The Dutch Development Instrument (DDI)

***4.1.1 Setting.*** The Dutch Youth Health Care (YHC) routinely monitors the development of almost all children living in The Netherlands. During the first four years, there are 13 scheduled visits. During these visits, the YHC professionals evaluate the growth and development of the child.

The *Dutch Development Instrument* (DDI; in Dutch: *Van Wiechenschema*) is the standard instrument used to measure development during the ages 0-4 years. The DDI consists of 75 milestones. The instrument assesses three developmental domains:

1.   Fine motor, adaptation, personality and social behaviour;

2.   Communication;

3.   Gross motor.

The milestones form two sets, one for children aged 0–15 months, and another for children aged 15–54 months. The YHC professionals administer an age-appropriate subset of milestones at each of the scheduled visits, thus building a *longitudinal developmental profile* for each child.

***4.1.2 Description of SMOCC study.*** The Social Medical Survey of Children Attending Child Health Clinics (SMOCC) study is a nationally representative cohort of 2,151 children born in The Netherlands during the years 1988–1989 ( Herngreen *et al.*, 1994). The study monitored child development using observations made on the DDI during nine visits covering the first 24 months of life. The SMOCC study collected information during the first two years on 57 (out of 75) milestones.

The *standard* set in the DDI consists of relatively easy milestones that 90 per cent of the children can pass at the scheduled age. This set is designed to have maximal sensitivity for picking up delays in development. A distinctive feature of the SMOCC study was the inclusion of more difficult milestones beyond the standard set. The *additional* set originates from the next time point. The success rate on these milestones is about 50 per cent.

***4.1.3 Codebook of DDI 0–30 months.*** Table 4.1 shows the 57 milestones from the DDI for ages 0 - 30 months as administered in the SMOCC study. Items are sorted according to *debut*, the age at which the item appears in the DDI. The response to each milestone is either a PASS (1) or a FAIL (0). Children who did not pass a milestone at the debut age were re-measured on that milestone during the next visit. The process continued until the child passed the milestone.

**Table 4.1.** Codebook of DDI as used in the SMOCC study.

Item	Debut	Domain	Label
ddicmm029	1m	Communication	Reacts when spoken to
ddifmd001	1m	Fine motor	Eyes fixate
ddigmd052	1m	Gross motor	Moves arms equally well
ddigmd053	1m	Gross motor	Moves legs equally well
ddigmd056	1m	Gross motor	Lifts chin off table for a moment
ddicmm030	2m	Communication	Smiles in response (M; can ask parents)
ddifmd002	2m	Fine motor	Follows with eyes and head 30d < 0 > 30d
ddicmm031	3m	Communication	vocalizes in response
ddifmd003	3m	Fine motor	Hands open occasionally
ddifmm004	3m	Fine motor	Watches own hands
ddigmd054	3m	Gross motor	Stays suspended when lifted under the armpits
ddigmd057	3m	Gross motor	Lifts head to 45 degrees on prone position
ddicmd116	6m	Communication	Turn head to sound
ddifmd005	6m	Fine motor	Plays with hands in midline
ddigmd006	6m	Gross motor	Grasps object within reach
ddigmd055	6m	Gross motor	No head lag if pulled to sitting
ddigmd058	6m	Gross motor	Looks around to side with angle face-table 90
ddigmd059	6m	Gross motor	Flexes or stomps legs while being swung
ddicmm033	9m	Communication	Says dada, baba, gaga
ddifmd007	9m	Fine motor	Passes cube from hand to hand
ddifmd008	9m	Fine motor	Holds cube, grasps another one with other hand
ddifmm009	9m	Fine motor	Plays with both feet
ddigmm060	9m	Gross motor	Rolls over back to front
ddigmd061	9m	Gross motor	Balances head well while sitting
ddigmd062	9m	Gross motor	Sits on buttocks while legs stretched
ddicmm034	12m	Communication	Babbles while playing
ddicmm036	12m	Communication	Waves 'bye-bye' (M; can ask parents)
ddifmd010	12m	Fine motor	Picks up pellet between thumb and index finger
ddigmd063	12m	Gross motor	Sits in stable position without support
ddigmm064	12m	Gross motor	Crawls forward, abdomen on the floor
ddigmm065	12m	Gross motor	Pulls up to standing position
ddicmm037	15m	Communication	Uses two words with comprehension
ddicmd136	15m	Communication	Reacts to verbal request (M; can ask parents)
ddifmd011	15m	Fine motor	Puts cube in and out of a box
ddifmm012	15m	Fine motor	Plays 'give and take' (M; can ask parents)
ddigmm066	15m	Gross motor	Crawls, abdomen off the floor (M; can ask parents)
ddigmm067	15m	Gross motor	Walks while holding onto play-pen or furniture
ddicmm039	18m	Communication	Says three 'words'
ddicmd141	18m	Communication	Identifies two named objects
ddifmd013	18m	Fine motor	Tower of 2 cubes
ddifmm014	18m	Fine motor	Explores environment energetically (M; can ask parents)
ddigmd068	18m	Gross motor	Walks alone
ddigmd069	18m	Gross motor	Throws ball without falling
ddicmm041	24m	Communication	Says sentences with 2 words
ddicmd148	24m	Communication	Understands 'play' orders
ddifmd015	24m	Fine motor	Builds tower of 3 cubes
ddifmm016	24m	Fine motor	Imitates everyday activities (M; can ask parents)
ddigmd070	24m	Gross motor	Squats or bends to pick things up
ddigmd146	24m	Gross motor	Drinks from cup (M; can ask parents)
ddigmd168	24m	Gross motor	Walks well
ddicmm043	30m	Communication	Refers to self using 'me' or 'I' (M; can ask parents)
ddicmd044	30m	Communication	Points at 5 pictures in the book
ddifmd017	30m	Fine motor	Tower of 6 cubes
ddifmd018	30m	Fine motor	Places round block in board
ddifmm019	30m	Fine motor	Takes off shoes and socks (M; can ask parents)
ddifmd154	30m	Fine motor	Eats with spoon without help (M; can ask parents)
ddigmd071	30m	Gross motor	Kicks ball

### 4.2 Probability of passing a milestone given age

**Figure 4.1.**
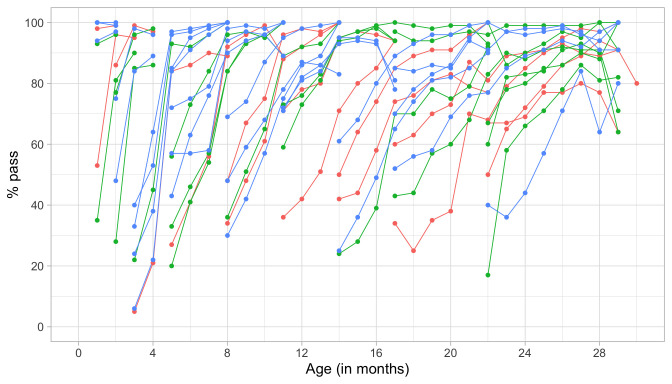
Empirical percentage of passing each milestone in the DDI against age (Source: SMOCC data, *n* = 2151, 9 occasions).

Figure 4.1 summarizes the response obtained on each milestone as a curve against age. The percentage of pass scores increases with age for all milestones. Note that curves on the left have steeper slopes than those on the right, thus indicating that development is faster for younger children.

The domain determines the coloured (blue: gross motor, green: fine motor, red: communication). In general, domains are well mixed across age, though around some ages, e.g., at four months, multiple milestones from the same domain appear.

### 4.3 Probability of passing a milestone given D-score

**Figure 4.2.**
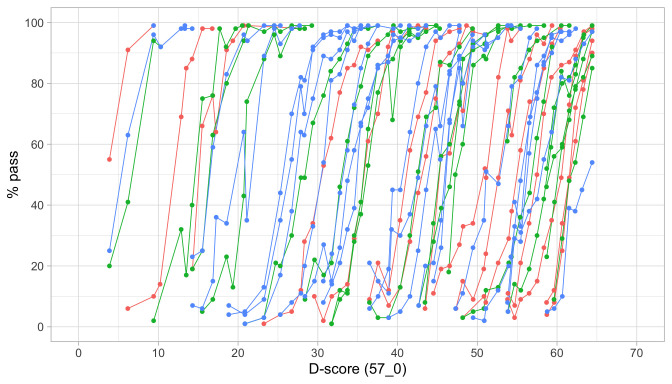
Empirical percentage of passing each milestone in the DDI against the D-score (Source: SMOCC data, 2151 children, 9 occasions).

Figure 4.2 is similar to Figure 4.1, but with the horizontal axis replaced by the D-score. The D-score summarizes development into one number. See 5.3 for a detailed explanation on how to calculate the D-score. The vertical axis with per cent pass is unchanged.

The percentage of successes increases with D-score for all milestones. In contrast to Figure 4.1 all curves have a similar slope, a desirable property needed for an interval scale with a constant unit of measurement (c.f. Section 3.4).

How can the relation between per cent pass and age be so different from the relation between per cent pass and the D-score? The next section explains the reason.

### 4.4 Relation between age and the D-score

**Figure 4.3.**
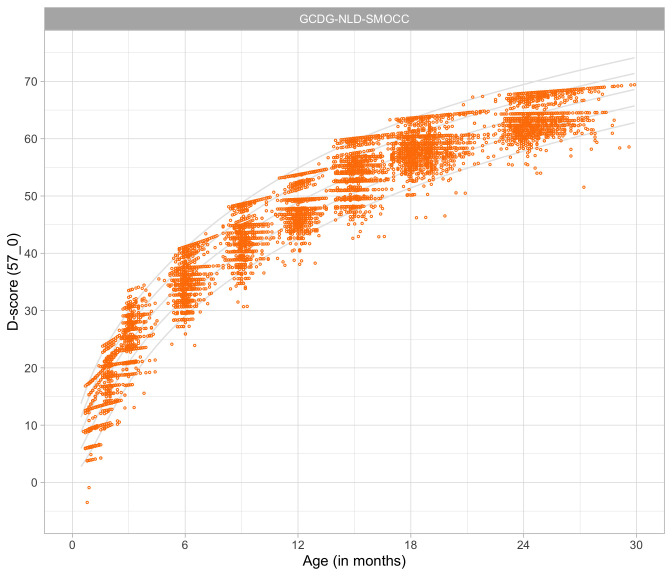
Relation between child D-score and child age in a cohort of Dutch children (Source: SMOCC data, *n* = 2151, 9 occasions).

Figure 4.3 shows that the relation between D-score and age is nonlinear. Development in the first year is more rapid than in the second year. During the first year, infants gain about 40 *D*, whereas in the second year they gain about 20 *D*. A similar change in growth rate occurs in length (first year: 23 cm, second year: 12 cm, for Dutch children).

**Figure 4.4.**
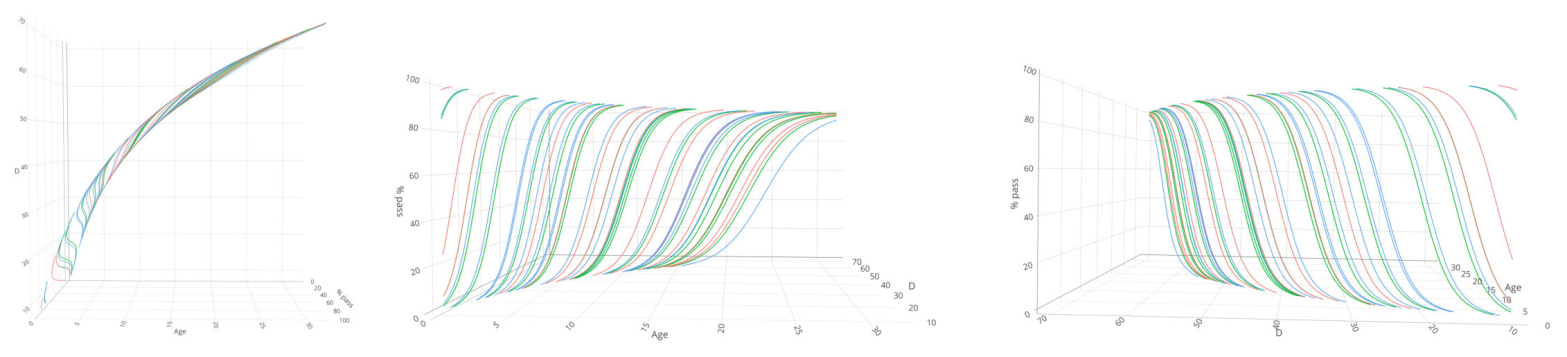
3D-line graph illustrating how the patterns in Figure 4.1 and Figure 4.2 induce the curvature in the relation between D-score and age. The printed version shows three orientations of the relation between age, percent pass and D-score. The online version holds an interactive 3D graph that the reader can actively manipulate the orientation of the graph by click-hold-drag mouse operations.

Figure 4.4 shows the mutual relations between age, percentage of milestone passing and the D-score. There are three main orientations.

•    In the default orientation (age on the horizontal axis, D-score on the vertical axis), we see a curvilinear relation between the age and item difficulty.

•    Rotate the graph (age on the horizontal axis, passing percentage on the vertical axis). Observe that this is the same pattern as in Figure 4.1 (with *unequal slopes*). Curves are coloured by domain.

•    Rotate the graph (D-score on the horizontal axis, passing percentage on the vertical axis). Observe that this pattern is the same as in Figure 4.2 (with *equal slopes*).

All patterns can co-exist because of the curvature in the relation between D-score and age. The curvature is never explicitly modelled or defined, but a consequence of the equal-slopes assumption in the relation between the D-score and the passing percentage of a milestone.

### 4.5 Measurement model for the D-score

***4.5.1 What are measurement models?***

From section 3.5 we quote:

        The measurement model specifies the relations between the data and the latent variable.

The term *Item Response Theory* (IRT) refers to the scientific theory of measurement models. Good introductory works include Embretsen & Reise (2000); Wright & Masters (1982) and Engelhard Jr. (2013).

IRT models enable quantification of the locations of both *items (milestones) and* persons* on the latent variable. We reserve the term *item* for generic properties, and *milestone* for child development. In general, items are part of the measurement instrument, persons are the objects to be measured.

An IRT model has three major structural components:

•    Specification of the underlying *latent variable(s)*. In this work, we restrict ourselves to models with just one latent variable. Multi-dimensional IRT models do have their uses, but they are complicated to fit and not widely used;

•    For a given item, a specification of the *probability of success* given a value on the latent variables. This specification can take many forms. Section 4.6 focuses on this in more detail;

•    Specification how probability models for the different items should be combined. In this work, we will restrict to models that assume *local independence* of the probabilities. In that case, the probability of passing two items is equal to the product of success probabilities.

***4.5.2 Adapt the model? Or adapt the data?*** The measurement model induces a predictable pattern in the observed items. We can test this pattern against the observed data. When there is misfit between the expected and observed data, we can follow two strategies:

•    Make the measurement model more general;

•    Discard items (and sometimes persons) to make the model fit.

These are very different strategies that have led to heated debates among psychometricians. See Engelhard Jr. (2013) for an overview.

In this work, we opt for the - rigorous - Rasch model ( Rasch (1960)) and will adapt the data to reduce discrepancies between model and data. Arguments for this choice are given later, in Section 4.8.

### 4.6 Item response functions

Most measurement models describe the probability of passing an item as a function of the *difference* between the person’s ability and the item’s difficulty. A person with low ability will almost inevitably fail a heavy item, whereas a highly able person will almost surely pass an easy item.

Let us now introduce a few symbols. We adopt the notation used in Wright & Masters (1982). We use *β _n_* (ability) to refer to the true (but unknown) developmental score of child *n*. Symbol *δ _i_* (difficulty) is the true (but unknown) difficulty of an item *i*, and *π _ni_* is the probability that child *n* passes item *i*. See Appendix A for a complete list.

The difference between the ability of child *n* and difficulty of item *i* is

$$\beta_{n}-\delta_{i}$$

In the special case that *β _n_ = δ _i_*, the person will have a probability of 0.5 of passing the item.

***4.6.1 Logistic model***. A widely used method is to express differences on the latent scale in terms of *logistic units* (or *logits*) ( Berkson, 1944). The reason preferring the logistic over the linear unit is that its output returns a probability value that maps to discrete events. In our case, we can describe the probability of passing an item (milestone) as a function of the difference between *β _n_* and *δ _i_* expressed in logits.

**Figure 4.5.**
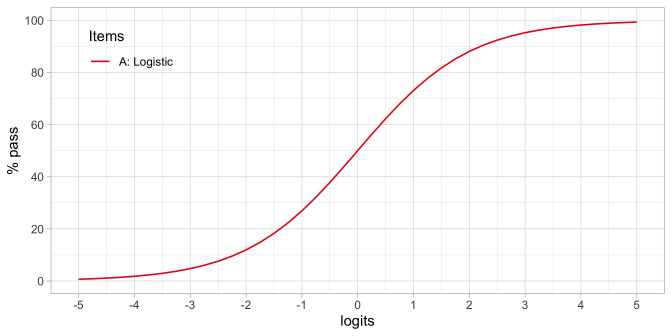
Standard logistic curve. Percentage of children passing an item for a given ability-difficulty gap *β _n_ – δ _i_*.

Figure 4.5 shows how the percentage of children that pass the item varies in terms of the ability-difficulty gap *β _n_ – δ _i_*. The gap can vary either by *β _n_* or *δ _i_* so that we may use the graph in two ways:

•    To find the probability of passing items with various difficulties for a child with ability *β _n_*. If *δ _i_* = *β _n_* then *π _ni_* = 0.5. If *δ _i_* < *β _n_* then *π _ni_* > 0.5, and if *δ _i_* > *β _n_* then *π _ni_* < 0.5. In words: If the difficulty of the item is equal to the child’s ability, then the child has a 50/50 chance to pass. The child will have a higher than 50/50 chance of passing for items with lower difficulty and have a lower than 50/50 chance of passing for items with difficulties that exceed the child’s ability.

•    To find the probability of passing a given item *δ _i_* for children that vary in ability. If *β _n_* < *δ _i_* then *π _ni_* < 0.5, and if *β _n_* > *δ _i_* then *π _ni_* > 0.5. In words: Children with abilities lower than the item’s difficulty will have lower than 50/50 chance of passing, whereas children with abilities that exceed the item’s difficulty will have a higher than 50/50 chance of passing.

Formula (4.1) defines the standard logistic curve:

One way to interpret the formula is as follows. The logarithm of the odds that a person with ability *β _n_* passes an item of difficulty *δ _i_* is equal to the difference *β _n_* – *δ _i_* ( Wright & Masters, 1982). For example, suppose that the probability that person *n* passes milestone *i* is *π _ni_* = 0.5. In that case, the odds of passing is equal to 0.5/(1 – 0.5) = 1, so log(1) = 0 and thus *β _n_* = *δ _i_*. If *β _n_* – *δ _i_* = log(2) = 0.693 person *n* is *two* times more likely to pass than to fail. Likewise, if the difference is *β _n_* – *δ _i_* = log(3) = 1.1, then person *n* is *three* more likely to pass. And so on.

***4.6.2 Types of item response functions***. The standard logistic function is by no means the only option to map the relationship between the latent variable and the probability of passing an item. The logistic function is the dominant choice in IRT, but it is instructive to study some other mappings. The *item response function* maps success probability against ability.

**Figure 4.6.**
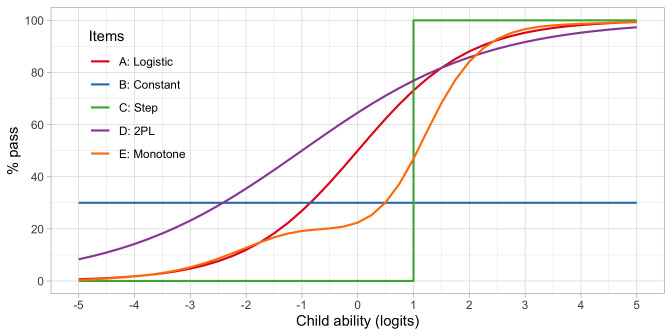
Item response functions for five hypothetical items, each demonstrating a positive relation between ability and probability to pass.

Figure 4.6 illustrates several other possibilities. Let us consider five hypothetical items, A–E. Note that the horizontal axis now refers to the ability, instead of the ability-item gap in 4.5.

•    A: Item A is the logistic function discussed in Section 4.6.

•    B: For item B, the probability of passing is constant at 30 per cent. This 30 per cent is not related to ability. Item B does not measure ability, only adds to the noise, and is of low quality.

•    C: Item C is a step function centred at an ability level of 1, so *all* children with an ability below 1 logit fail and *all* children with ability above 1 logit pass. Item C is the ideal item for discriminating children with abilities above and below 1. The item is not sensitive to differences at other ability levels, and often not so realistic in practice.

•    D: Like A, item D is a smoothly increasing logistic function, but it has an extra parameter that allows it to vary its slope (or discrimination). The extra parameter can make the curve steeper (more discriminatory) than the red curve, in the limit approaching a step curve. It can also become shallower (less discriminatory) than the red curve (as plotted here), in the limit approaching a constant curve (item B). Thus, item D generalizes items A, B or C.

•    E: Item E is even more general in the sense that it need not be logistic, but a general monotonically increasing function. As plotted, the item is insensitive to abilities between -1 and 0 logits, and more sensitive to abilities between 0 to 2 logits.

These are just some examples of how the relationship between the child’s ability and passing probability could look. In practice, the curves need not start at 0 per cent or end at 100 per cent. They could also be U-shaped, or have other non-monotonic forms. See Coombs (1964) for a thorough overview of such models. In practice, most models are restricted to shapes A-D.

***4.6.3 Person response functions***. We can reverse the roles of persons and items. The *person response function* tells us how likely it is that a single person can pass an item, or more commonly, a set of items.

Let us continue with items A, C and D from Figure 4.6, and calculate the response function for three children, respectively with abilities *β*_1_ = –2, *β*_2_ = 0 and *β*_3_ = 2.

**Figure 4.7.**
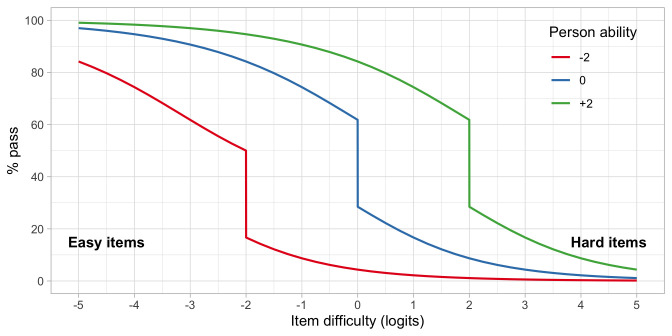
Person response functions for three children with abilities -2, 0 and +2, using a small test of items A, C and D.

Figure 4.7 presents the person response functions from three persons with abilities of -2, 0 and +2 logits. We calculate the functions as the average of response probabilities on items A, C and D. Thus, on average, we expect that child 1 logit will pass an easy item of difficulty -3 in about 60 per cent of the time, whereas for an intermediate item of difficulty of -1 the passing probability would be 10 per cent. For child 3, with higher ability, these probabilities are quite different: 97% and 90%. The substantial drop in the middle of the curve is due to the step function of item A.

### 4.7 Engelhard criteria for invariant measurement

In this work, we strive to achieve *invariant measurement*, a strict form of measurements that is subject to the following requirements ( Engelhard Jr., 2013, 14):

1. *Item-invariant measurement of persons*: The measurement of persons must be independent of the particular items used for the measuring.

2. *Non-crossing person response functions*: A more able person must always have a better chance of success on an item that a less able person.

3. *Person-invariant calibration of test items*: The calibration of the items must be independent of the particular persons used for calibration.

4. *Non-crossing item response functions*: Any person must have a better chance of success on an easy item than on a more difficult item.

5. *Unidimensionality*: Items and persons take on values on a *single* latent variable. Under this assumption, the relations between the items are fully explainable by the scores on the latent scale. In practice, the requirement implies that items should measure the same construct. ( Hattie, 1985)

Three families of IRT models support invariant measurement:

1.   Scalogram model ( Guttman, 1950)

2.   Rasch model ( Andrich, 1978; Rasch, 1960; Wright & Masters, 1982)

3.   Mokken scaling model ( Mokken, 1971; Molenaar, 1997)

The Guttman and Mokken models yield an ordinal latent scale, while the Rasch model yields an interval scale (with a constant unit).

### 4.8 Why take the Rasch model?

• *Invariant measurement*: The Rasch model meets the five Engelhard criteria (c.f. Section 4.7).

• *Interval scale*: When it fits, the Rasch model provides an interval scale, the de-facto requirement for any numerical comparisons (c.f. Section 3.4.1).

• *Parsimonious*: The Rasch model has one parameter for each item and one parameter for each person. The Rash model one of the most parsimonious IRT models, and can easily be applied to thousands of items and millions of persons.

• *Specific objectivity*: Person and item parameters are mathematically separate entities in the Rasch model. In practice, this means that the estimated difference in ability between two persons does not depend on the difficulty of the test. Also, the estimated differences in difficulties between two items do not depend on the abilities in the calibration sample. The property is especially important in the analysis of combined data, where abilities can vary widely between sources. See Rasch (1977) for derivations and examples.

• *Unified model*: The Rasch model unifies distinct traditions in measurement theory. One may derive the Rasch model from

– Thorndike’s 1904 criteria
– Guttman scalogram model
– Ratio-scale counts
– Raw scores as sufficient statistics
– Thurstone’s scaling requirements
– Campbell concatenation
– Rasch’s specific objectivity

• *Fits child development data*: Last but not least, as we will see in Section 6, the Rasch model provides an excellent fit to child development milestones.

## 5 Computation

This section explains the basic computations needed for fitting and evaluating the Rasch model. We distinguish the following steps:

•    Identify nature of the problem (5.1)

•    Estimation of item parameters (5.2)

•    Anchoring (5.2.2)

•    Estimation of the D-score (5.3)

•    Estimation of age-conditional references (5.4)

Readers not interested in these details may continue to model evaluation in Section 6.

### 5.1 Identify nature of the problem

The SMOCC dataset, introduced in Section 4.1.2, contains scores on the DDI of Dutch children aged 0-2 years made during nine visits.

Table 5.1 contains data of three children, measured on nine visits between ages 0 – 2 years. The DDI scores take values 0 (FAIL) and 1 (PASS). In order to save horizontal space, we truncated the column headers to the last two digits of the item names.

**Table 5.1.** SMOCC DDI milestones, first three children, 0–2 years.

29	30	31	33	34	36	37	39	41	43	44	16	36	41	48	01	02	03	04	05	07	08	09	10	11	12	13	14	15	16	17	18	19	54	06	52	53	54	55	56	57	58	59	60	61	62	63	64	65	66	67	68	69	70	71	46	68
**1**	**0**														**1**	**0**																			**1**	**1**			**1**																	
	**1**															**1**																																								
		**1**									**0**						**1**	**1**	**0**															**0**			**1**	**1**		**1**	**1**	**1**														
			**0**								**1**								**1**	**1**	**0**	**1**												**1**				**1**			**1**	**1**	**0**	**1**	**1**											
			**1**																	**1**	**0**	**1**																					**1**	**1**	**1**	**1**										
				**1**	**1**	**1**						**1**											**1**																							**1**	**0**	**1**								
						**1**						**1**												**1**	**1**																								**0**	**0**						
							**1**																			**0**	**1**																								**0**	**0**				
								**1**						**1**															**1**																										**0**	**1**
**1**	**1**														**1**	**1**																			**1**	**1**			**1**																	
	**1**															**1**																																								
		**1**									**0**						**1**	**1**	**0**															**0**			**1**	**0**		**1**	**0**	**0**														
			**0**								**1**								**1**	**0**	**0**	**1**												**1**				**1**			**1**	**0**	**1**	**0**	**1**											
			**1**																	**1**	**1**	**1**																					**1**	**1**	**1**	**0**		**1**								
				**1**	**1**																		**1**	**1**																						**1**	**1**	**1**	**1**	**1**						
								**1**		**1**				**1**														**1**	**1**		**1**		**1**																				**1**		**1**	**1**
							**1**																			**0**	**1**																								**1**					
**1**	**0**														**1**	**0**																			**1**	**1**			**1**																	
	**1**															**0**																																								
		**1**									**0**						**1**	**1**	**0**															**0**			**1**	**0**		**1**	**1**	**0**														
			**1**								**1**								**1**	**0**	**0**	**1**												**0**				**1**			**1**	**1**	**1**	**1**	**1**											
			**1**	**1**																**1**	**1**	**1**																					**1**	**1**	**1**	**0**	**0**									
				**1**	**1**																		**1**																							**1**	**1**	**0**								
						**0**						**0**												**1**	**1**																								**1**	**1**						
							**1**						**1**													**0**	**1**		**1**																						**1**	**0**	**1**			**1**
								**1**	**1**	**1**				**1**														**1**	**1**		**1**	**1**	**1**																				**1**		**1**	**1**

Since the selection of milestones depends on age, the dataset contains a large number of empty cells. Naive use of sum scores as a proxy to ability is therefore problematic. An empty cell is not a FAIL, so it is incorrect to impute those cells by zeroes.

Note that some rows contain only 1’s, e.g., in row 2. Many computer programs for Rasch analysis routinely remove such *perfect scores* before fitting. However, unless the number of perfect scores is very small, this is not recommended because doing so can severely affect the ability distribution.

In order to effectively handle the missing data and to preserve all persons in the analysis we separate estimation of item difficulties (c.f. Section 5.2) and person abilities (c.f. Section 5.3).

### 5.2 Item parameter estimation

***5.2.1 Pairwise estimation of item difficulties***. There are many methods for estimating the difficulty parameters of the Rasch estimation. See Linacre (2004) for an overview.

We will use the pairwise estimation method. This method writes the probability that child *n* passes item *i* but not item *j* given that the child passed one of them as exp( *δ _i_*)/(exp( *δ _i_*) + exp( *δ _j_*)). The method optimizes the pseudo-likelihood of all item pairs over the difficulty estimates by a simple iterative procedure.

Zwinderman (1995) has shown that this procedure provides consistent estimates with similar efficiency computationally more-intensive conditional and marginal maximum likelihood methods.

The beauty of the method is that it is independent of the ability distribution, so there is no need to remove perfect scores. We use the function rasch.pairwise.itemcluster() as implemented in the sirt package ( Robitzsch, 2016).

**Figure 5.1.**
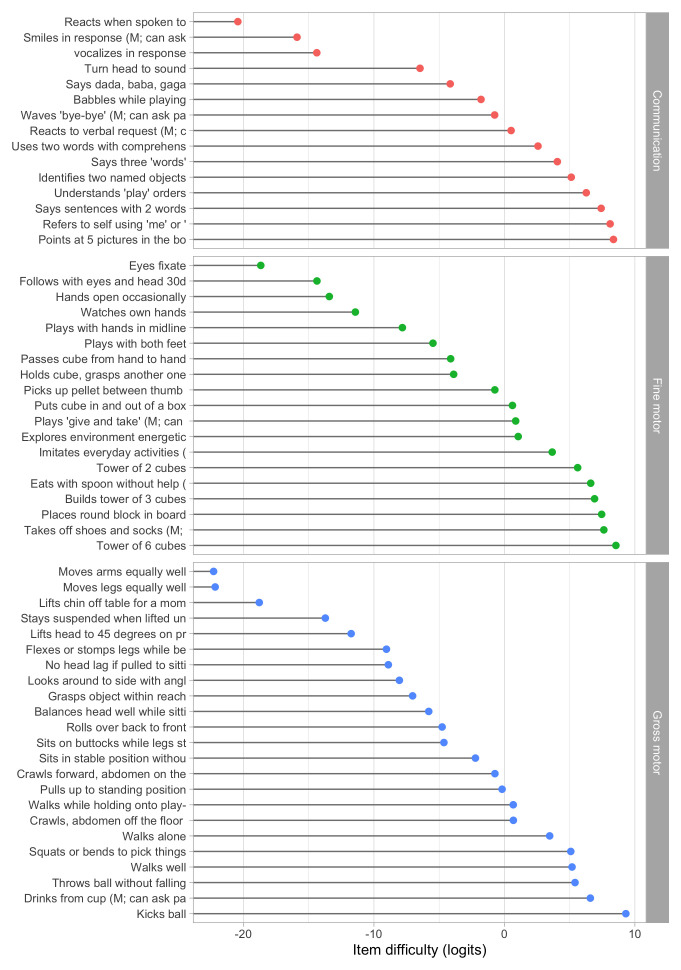
Estimated item difficulty parameters ( *d _i_*) for 57 milestones of the DDI (0 – 2 years).

Figure 5.1 summarizes the estimated item difficulty parameters. Although the model makes no distinction between domains, the results have been ordered to ease spotting of the natural progression of the milestones per domain. The figure also suggests that not all domain have equal representation across the scale. For example, there are no communication milestones around the logit of –10.

***5.2.2 Anchoring***. The Rasch model identifies the item difficulties up to a linear transformation. By default, the software produces estimates in the logit scale (c.f. Figure 5.1). The logit scale is inconvenient for two reasons:

•    The logit scale has negative values. Negative values do not have a sensible interpretation in child development, and are likely to introduce errors in practice;

•    Both the zero in the logit scale, as well as its variance, depend on the sample used to calibrate the item difficulties.

**Figure 5.2.**
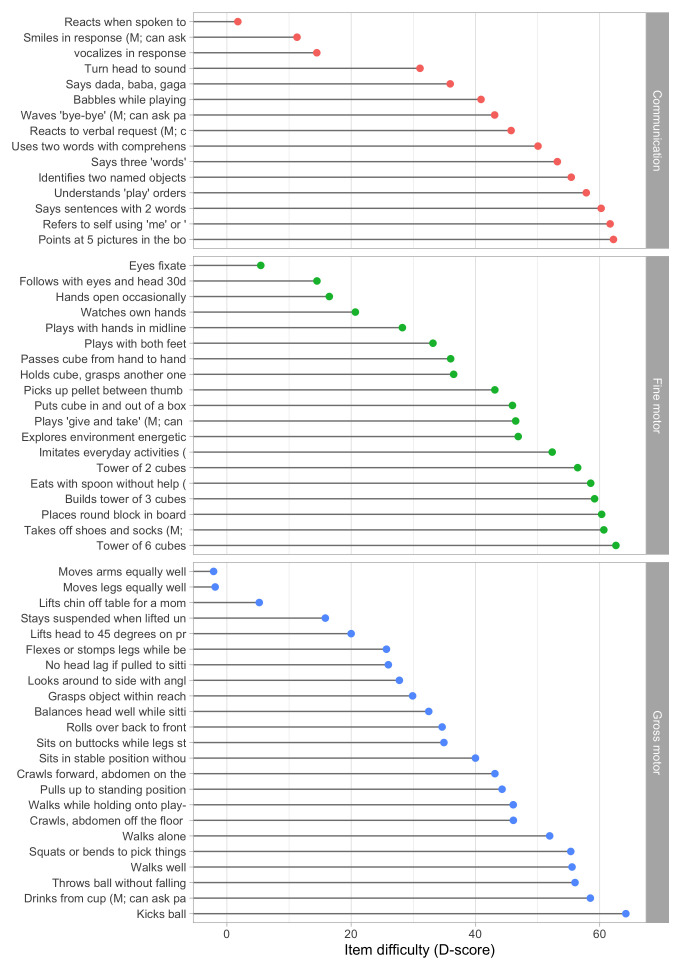
Estimated item difficulty parameters ( *d _i_*) for 57 milestones of the DDI (0 – 2 years). Milestones ddigmd057 and ddigmd063 are anchored at values of 20 *D* and 40 *D*, respectively.

Rescaling preserves the properties of the Rasch model. To make the scale independent of the specified sample, we transform the scale so that two items will always have the same value on the transformed scale. The choice of the two anchor items is essentially arbitrary, but they should correspond to milestones that are easy to measure with small error. In the sequel, we use the two milestones to anchor the D-score scale:

With the choice of Table 5.2, D-score values are approximately 0 *D* around birth. At the age of 1 year, the score will around 50 *D*, so during the first year of life, one *D* unit corresponds to approximately a one-week interval. Figure 5.2 shows the difficulty estimates in the D-score scale.

**Table 5.2.** Anchoring values used to identify the D-score scale.

Item	Label	Value
ddigmd057	Lifts head to 45 degrees on prone position	20
ddigmd063	Sits in stable position without support	40

### 5.3 Estimation of the D-score

The second part of the estimation process is to estimate a D-score. The D-score quantifies the development of a child at a given age. Whereas the instrument developer is responsible for the estimation of item parameters, D-score estimation is more of a task for the user. To calculate the D-score, we need the following ingredients:

•    Child’s PASS/FAIL scores on the milestones administered;

•    The difficulty estimates of each milestone administered;

•    A prior distribution, an estimate of the D-score distribution before seeing any PASS/FAIL score.

Using these inputs, we may use Bayes theorem to calculate the position of the person on the latent variable.

***5.3.1 Role of the starting prior***. The first two inputs to the D-score will be self-evident. The third component, the prior distribution, is needed to be able to deal with perfect responses. The prior distribution summarizes our knowledge about the D-score before we see any of the child’s PASS/FAIL scores. In general, we like the prior to be non-informative, so that the observed responses and item difficulties entirely determine the value of the D-score. In practice, we cannot use truly non-informative prior because that would leave the D-score for perfect responses (i.e., all PASS or all FAIL) undefined. The choice of the prior is essentially arbitrary, but we can make it in such a way that its impact on the value D-score is negligible, especially for tests where we have more than, say, four items.

Since we know that the D-score depends on age, a logical choice for the prior is to make it dependent on age. In particular, we will define the prior as a normal distribution equal to the expected mean in Figure 4.3 at the child’s age, and with a standard deviation that considerably higher than in Figure 4.3. Numerical example: the mean D-score at the age of 15 months is equal to 53.6 *D*. The standard deviation in Figure 4.3 varies between 2.6 *D* and 3.0 *D*, with an average of 2.9 *D*. After some experimentation, we found that using a value of 5.0 *D* for the prior yields a good compromise between non-informativeness and robustness of D-score estimates for perfect patterns. The resulting starting prior for a child aged 15 months is thus *N*(53.6,5).

The reader now probably wonders about a chicken-and-egg problem: To calculate the D-score, we need a prior, and to determine the prior we need the D-score. So how did we calculate the D-scores in Figure 4.3? The answer is that we first took at rougher prior, and calculated two temporary models in succession using the D-scores obtained after solution 1 to inform the prior before solution 2, and so on. It turned out that D-scores in Figure 4.3 hardly changed after two steps, and so there we stopped.

**Figure 5.3.**
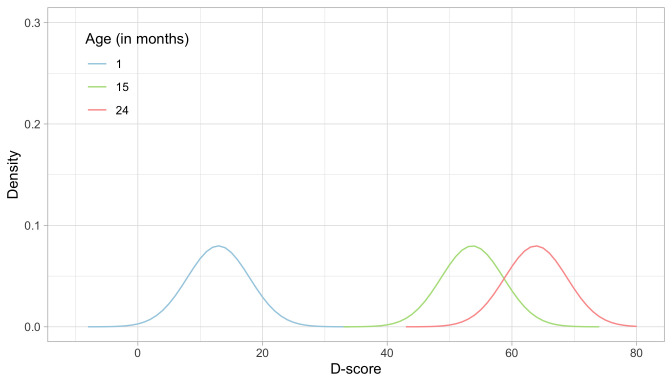
Age-dependent starting priors for the D-score at the ages of 1, 15 and 24 months.

***5.3.2 Starting prior: Numerical example***. Figure 5.3 illustrates starting distributions (priors) chosen according to the principles set above for the ages of 1, 15 and 24 months. As expected, the assumed ability of an infant aged one month is much lower than that of a child aged 15 months, which in turn is lower than the ability of a toddler aged 24 months. The green distribution for 15 months corresponds to the normal distribution *N* (53.6,5).

Another choice that we need to make is the grid of points on which we calculate the prior and posterior distributions. Figure 5.3 uses a grid from -10 *D* to +80 *D*, with a step size of 1 *D*. These are fixed *quadrature points*, and there are 91 of them. While these quadrature points are sufficient to estimate D-score for ages up to 2.5 years, it is wise to extend the range for older children with higher D-scores.

***5.3.3 EAP algorithm***. The algorithm for estimating the D-score is known as the Expected a posteriori (EAP) method, first described by Bock & Mislevy (1982). Calculation of the D-score proceeds item by item. Suppose we have some vague and preliminary idea about the distribution of *D*, the starting prior (c.f. section 5.3.1), based on age. The procedure uses Bayes rule to update this prior knowledge with data from the first item (using the child’s FAIL/PASS score and the estimated item difficulty) to calculate the posterior. The next step uses this posterior as prior before processing the next item, and so on. The procedure stops when the item pool is exhausted. The order in which items enter does not matter for the result. The D-score is equal to the mean of the posterior calculated after the last question.

***5.3.4 EAP algorithm: Numerical example***. Suppose we measure two boys aged 15 months, David and Rob, by the DDI. David passes the first four milestones but does not complete the test. Rob completes the test but fails on two out of five items.

Table 5.3 shows the difficulty of each milestone (in the column labelled “Delta”), and the responses of David and Rob for the standard five DDI milestones for the age of 15 months.

**Table 5.3.** Scores of David and Rob on five milestones from the DDI.

item	label	delta	David	Rob
ddifmd011	Puts cube in and out of a box	46.0	1	1
ddifmm012	Plays 'give and take' (M; can ask parents)	46.5	1	0
ddicmm037	Uses two words with comprehension	50.1	1	1
ddigmm066	Crawls, abdomen off the floor (M; can ask parents)	46.1	1	1
ddigmm067	Walks while holding onto play-pen or furniture	46.1		0

The mean D-score for Dutch children aged 15 months is 53.6 *D*, so the milestones are easy to pass at this age, with the most difficult is ddicmm037. David passed all milestones but has no score on the last. Rob fails on ddifmm012 and ddigmm067. How do we calculate the D-score for David and Rob?

**Figure 5.4.**
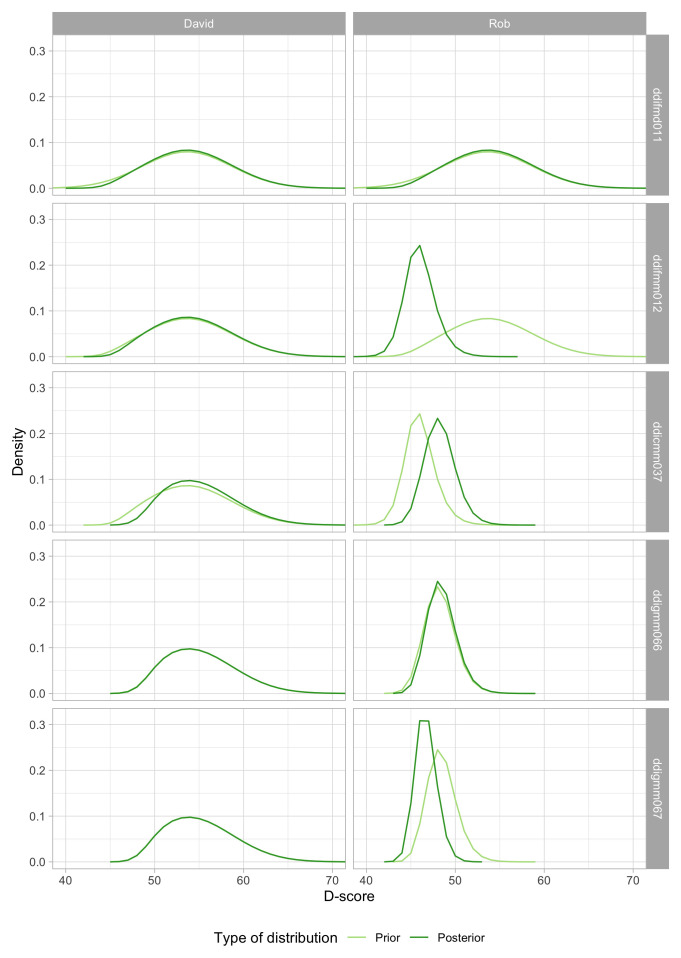
D-score distribution for David and Rob before (prior) and after (posterior) a milestone is taken into account.

Figure 5.4 shows how the prior transforms into the posterior after we successively feed the measurements into the calculation. There are five milestones, so the calculation comprises five steps:

1.    Both David and Rob pass ddifmd011. The prior (light green) is the same as in Figure 5.3. After a PASS, the posterior will be located more to the right, and will often be more peaked. Both happen here, but the change is small. The reason is that a PASS on this milestone is not very informative. For a child with a true D-score of 53 *D*, the probability of passing ddifmd011 is equal to 0.966. If passing is so common, there is not much information in the measurement.

2.    David passes ddifmm012, but Rob does not. Observe that the prior is identical to the posterior of ddifmd011. For David, the posterior is only slightly different from the prior, for the same reason as above. For Rob, we find a considerable change to the left, both for location (from 54.3 *D* to 47.1 *D*) and peakedness. This one FAIL lowers Rob’s score by 7.2 *D*.

3.    Milestone ddicmm037 is more difficult than the previous two milestones, so a pass on ddicmm037 does have a definite effect on the posterior for both David and Rob.

4.    David’s PASS on ddigmm066 does not bring any additional information, so his prior and posterior are virtually indistinguishable. For Rob, we find a slight shift to the right.

5.    There is no measurement for David on ddigmm067, so the prior and posterior are equivalent. For Rob, we observe a FAIL, which shifts his posterior to the left.

We calculate the D-score as the mean of the posterior. David’s D-score is equal to 55.7 *D*. Note that the measurement error, as estimated from the variance of the posterior, is relatively large. Rob’s D-score is equal to 47.7 *D*, with a much smaller measurement error. This result is consistent with the design principles of the DDI, which is meant to detect children with developmental delay.

The example illustrates that the quality of the D-score depends on two factors, the match between the true (but unknown) D-score of the child and the difficulty of the milestone.

***5.3.5 Technical observations on D-score estimation***

•    Administration of a too easy set of milestones introduces a *ceiling* with children that pass all milestones, but whose true D-score could extend well beyond the maximum. Depending on the goal of the measurement, this may or may not be a problem.

•    The specification of the prior and posterior distributions requires a set of quadrature points. The quadrature points are taken here as the static and evenly-spaced set of integers between -10 and +80. Using other quadrature points may affect the estimate, especially if the range of the quadrature points does not cover the entire D-score range.

•    The actual calculations are here done item by item. A more efficient method is to handle all responses at once. The result will be the same.

### 5.4 Age-conditional references

***5.4.1 Motivation***. The last step involves estimation an age-conditional reference distribution for the D-score. This distribution can be used to construct growth charts that portray the normal variation in development. Also, the references can be used to calculate age-standardized D-scores, called DAZ, that emphasize the location of the measurement in comparison to age peers.

Estimation of reference centiles is reasonably standard. Here we follow van Buuren (2014) to fit age-conditional references of the D-score for boys and girls combined by the LMS method. The LMS method by Cole & Green (1992) assumes that the outcome has a normal distribution after a Box-Cox transformation. The reference distribution has three parameters, which model respectively the location ( *M*), the spread ( *S*), and the skewness ( *L*) of the distribution. Each of the three parameters can vary smoothly with age.

***5.4.2 Estimation of the reference distribution***. The parameters are estimated using the BCCG distribution of gamlss 5.1-3 ( Stasinopoulos & Rigby, 2008) using cubic splines smoothers. The final solution used a log-transformed age scale and fitted the model with smoothing parameters df( *M*) = 2, df( *S*) = 2 and df( *L*) = 1.

Figure 4.3 plots the D-scores together with five grey lines, corresponding to the centiles -2SD (P2), -1SD (P16), 0SD (P50), +1SD (P84) and +2SD (P98). The area between the -2SD and +2SD lines delineates the D-score expected if development is healthy. Note that the shape of the reference is quite similar to that of weight and height, with rapid growth occurring in the first few months.

Table 5.4 defines age-conditional references for Dutch children as the *M*-curve (median), *S*-curve (spread) and *L*-curve (skewness) by age. This table can be used to calculate centile lines and *Z*-scores.

**Table 5.4.** Dutch reference values for the D-score. M-curve (median), S-curve (spread) and L-curve (skewness).

Age	M	S	L
0.0383	8.81	0.3126	1.3917
0.0575	10.59	0.2801	1.4418
0.0767	12.27	0.2526	1.4891
0.0958	13.87	0.2291	1.5331
0.1150	15.39	0.2089	1.5722
0.1342	16.83	0.1916	1.6049
0.1533	18.20	0.1767	1.6304
0.1725	19.50	0.1640	1.6487
0.1916	20.75	0.1531	1.6607
0.2108	21.94	0.1436	1.6676
0.2300	23.07	0.1354	1.6706
0.2491	24.16	0.1283	1.6711
0.2683	25.21	0.1220	1.6698
0.2875	26.21	0.1165	1.6673
0.3066	27.17	0.1117	1.6636
0.3258	28.10	0.1074	1.6589
0.3450	28.99	0.1035	1.6533
0.3641	29.86	0.1001	1.6471
0.3833	30.70	0.0970	1.6403
0.4025	31.50	0.0942	1.6330
0.4216	32.29	0.0917	1.6255
0.4408	33.05	0.0894	1.6178
0.4600	33.79	0.0873	1.6100
0.4791	34.51	0.0854	1.6022
0.4983	35.21	0.0837	1.5946
0.5175	35.89	0.0821	1.5870
0.5366	36.55	0.0807	1.5797
0.5558	37.20	0.0793	1.5725
0.5749	37.83	0.0781	1.5656
0.5941	38.44	0.0770	1.5588
0.6133	39.04	0.0759	1.5523
0.6324	39.63	0.0749	1.5460
0.6516	40.21	0.0740	1.5399
0.6708	40.77	0.0731	1.5340
0.6899	41.32	0.0723	1.5284
0.7091	41.86	0.0715	1.5230
0.7283	42.39	0.0707	1.5178
0.7474	42.91	0.0700	1.5128
0.7666	43.42	0.0693	1.5081
0.7858	43.92	0.0687	1.5036
0.8049	44.40	0.0681	1.4993
0.8241	44.88	0.0674	1.4952
0.8433	45.36	0.0669	1.4913
0.8624	45.82	0.0663	1.4876
0.8816	46.27	0.0657	1.4841
0.9008	46.72	0.0652	1.4809
0.9199	47.16	0.0647	1.4778
0.9391	47.59	0.0642	1.4749
0.9582	48.01	0.0637	1.4723
0.9774	48.43	0.0632	1.4698
0.9966	48.84	0.0627	1.4676
1.0157	49.24	0.0622	1.4655
1.0349	49.64	0.0618	1.4637
1.0541	50.03	0.0613	1.4620
1.0732	50.41	0.0608	1.4605
1.0924	50.79	0.0604	1.4592
1.1116	51.16	0.0600	1.4580
1.1307	51.53	0.0595	1.4570
1.1499	51.89	0.0591	1.4561
1.1691	52.24	0.0587	1.4553
1.1882	52.59	0.0583	1.4547
1.2074	52.94	0.0578	1.4542
1.2266	53.27	0.0574	1.4538
1.2457	53.61	0.0570	1.4535
1.2649	53.94	0.0566	1.4534
1.2841	54.26	0.0562	1.4533
1.3032	54.58	0.0559	1.4533
1.3224	54.89	0.0555	1.4533
1.3415	55.20	0.0551	1.4535
1.3607	55.50	0.0547	1.4537
1.3799	55.81	0.0544	1.4539
1.3990	56.10	0.0540	1.4542
1.4182	56.39	0.0536	1.4546
1.4374	56.68	0.0533	1.4551
1.4565	56.97	0.0530	1.4555
1.4757	57.25	0.0526	1.4561
1.4949	57.52	0.0523	1.4567
1.5140	57.80	0.0520	1.4573
1.5332	58.06	0.0517	1.4580
1.5524	58.33	0.0514	1.4587
1.5715	58.59	0.0510	1.4595
1.5907	58.85	0.0508	1.4603
1.6099	59.11	0.0505	1.4612
1.6290	59.36	0.0502	1.4620
1.6482	59.61	0.0499	1.4630
1.6674	59.86	0.0496	1.4639
1.6865	60.11	0.0494	1.4649
1.7057	60.35	0.0491	1.4660
1.7248	60.59	0.0488	1.4670
1.7440	60.82	0.0486	1.4681
1.7632	61.06	0.0483	1.4692
1.7823	61.29	0.0481	1.4704
1.8015	61.52	0.0478	1.4716
1.8207	61.75	0.0476	1.4728
1.8398	61.97	0.0474	1.4740
1.8590	62.20	0.0471	1.4752
1.8782	62.42	0.0469	1.4765
1.8973	62.64	0.0467	1.4778
1.9165	62.85	0.0465	1.4791
1.9357	63.07	0.0463	1.4805
1.9548	63.28	0.0461	1.4818
1.9740	63.49	0.0459	1.4832
1.9932	63.70	0.0457	1.4846
2.0123	63.91	0.0455	1.4861
2.0315	64.11	0.0453	1.4875
2.0507	64.32	0.0451	1.4890
2.0698	64.52	0.0449	1.4904
2.0890	64.72	0.0447	1.4919
2.1081	64.92	0.0445	1.4934
2.1273	65.11	0.0443	1.4949
2.1465	65.31	0.0441	1.4964
2.1656	65.50	0.0440	1.4979
2.1848	65.70	0.0438	1.4994
2.2040	65.89	0.0436	1.5009
2.2231	66.08	0.0434	1.5024
2.2423	66.26	0.0433	1.5039
2.2615	66.45	0.0431	1.5054
2.2806	66.64	0.0429	1.5069
2.2998	66.82	0.0428	1.5084
2.3190	67.00	0.0426	1.5098
2.3381	67.18	0.0425	1.5113
2.3573	67.36	0.0423	1.5127
2.3765	67.54	0.0421	1.5142
2.3956	67.72	0.0420	1.5156
2.4148	67.89	0.0418	1.5170
2.4339	68.07	0.0417	1.5185
2.4531	68.24	0.0415	1.5199
2.4723	68.41	0.0414	1.5213
2.4914	68.59	0.0412	1.5226
2.5106	68.75	0.0411	1.5240
2.5298	68.92	0.0410	1.5254
2.5489	69.09	0.0408	1.5267
2.5681	69.26	0.0407	1.5281
2.5873	69.42	0.0405	1.5294
2.6064	69.59	0.0404	1.5308
2.6256	69.75	0.0403	1.5321
2.6448	69.91	0.0401	1.5334
2.6639	70.07	0.0400	1.5347
2.6831	70.23	0.0399	1.5360
2.7023	70.39	0.0397	1.5373
2.7214	70.55	0.0396	1.5386
2.7406	70.71	0.0395	1.5398
2.7598	70.86	0.0394	1.5411
2.7789	71.02	0.0392	1.5423

The references are purely cross-sectional and do not account for the correlation structure between ages. For prediction purposes, it is useful to extend the modelling to include velocities and change scores.

***5.4.3 Conversion of *D* to DAZ, and vice versa***. Suppose that *M _t_*, *S _t_* and *L _t_* are the parameter values at age *t*. Cole (1988) shows that the transformation

$$Z=\frac{{\left(D_{t}/M_{t}\right)}^{L_{t}}-1}{L_{t}S_{t}}$$

converts measurement *D _t_* into its normal equivalent deviate *Z*. If *L _t_* is close to zero, we use

$$Z=\frac{\ln(D_{t}/M_{t})}{S_{t}}$$

We may derive any required centile curve from Table 5.4. First, choose *Z _α_* as the *Z*-score that delineates 100 *α* per cent of the distribution, for example, *Z*_0.05_ = –1.64. The D-score that defines the 100 *α* centile is equal to

$$D_{t}(\alpha )=M_{t}{\left(1+L_{t}S_{t}Z_{\alpha }\right)}^{1/L_{t}}$$

If *L _t_* is close to zero, we use

$$D_{t}(\alpha )=M_{t}\exp(S_{t}Z_{\alpha }).$$

## 6 Evaluation

The properties cut-off Rasch model (c.f. Section 4.8) only hold when the data and model agree. It is, therefore, essential to study and remove discrepancies between model and data. This section explains several techniques that aid in the evaluation of model fit.

•    Item fit (6.1)

•    Person fit (6.2)

•    Differential item functioning (6.3)

•    Item information (6.4)

•    Reliability (6.5)

These topics address different aspects of the solution. In practice, we have found that item fit is the most critical concern.

### 6.1 Item fit

The philosophy of the Rasch model is different from conventional statistical modelling. It is not the task of the Rasch model to account for the data. Rather it is the task of the data to fit the Rasch model. We saw this distinction before in Section 4.5.2.

The goal of model-fit assessment is to explore and quantify how well empirical data meet the requirements of the Rasch model. One way to gauge model-fit is to compare the observed probability of passing an item to the fitted item response curve for endorsing the item.

The fitted item response curve for each item *i* is modeled as:

$$P_{ni}=\frac{\exp({\hat{\beta }}_{n}-{\hat{\delta }}_{i})}{1+\exp({\hat{\beta }}_{n}-{\hat{\delta }}_{i})},$$

where ${\hat{\beta }}_{n}$ is the estimated ability of child *n* (the child’s D-score), and where ${\hat{\delta }}_{i}$ is the estimated difficulty of item *i*. This is equivalent to formula (4.1) with the parameters replaced by estimates. Section 5 described process of parameter estimation in some detail.

**Figure 6.1.**
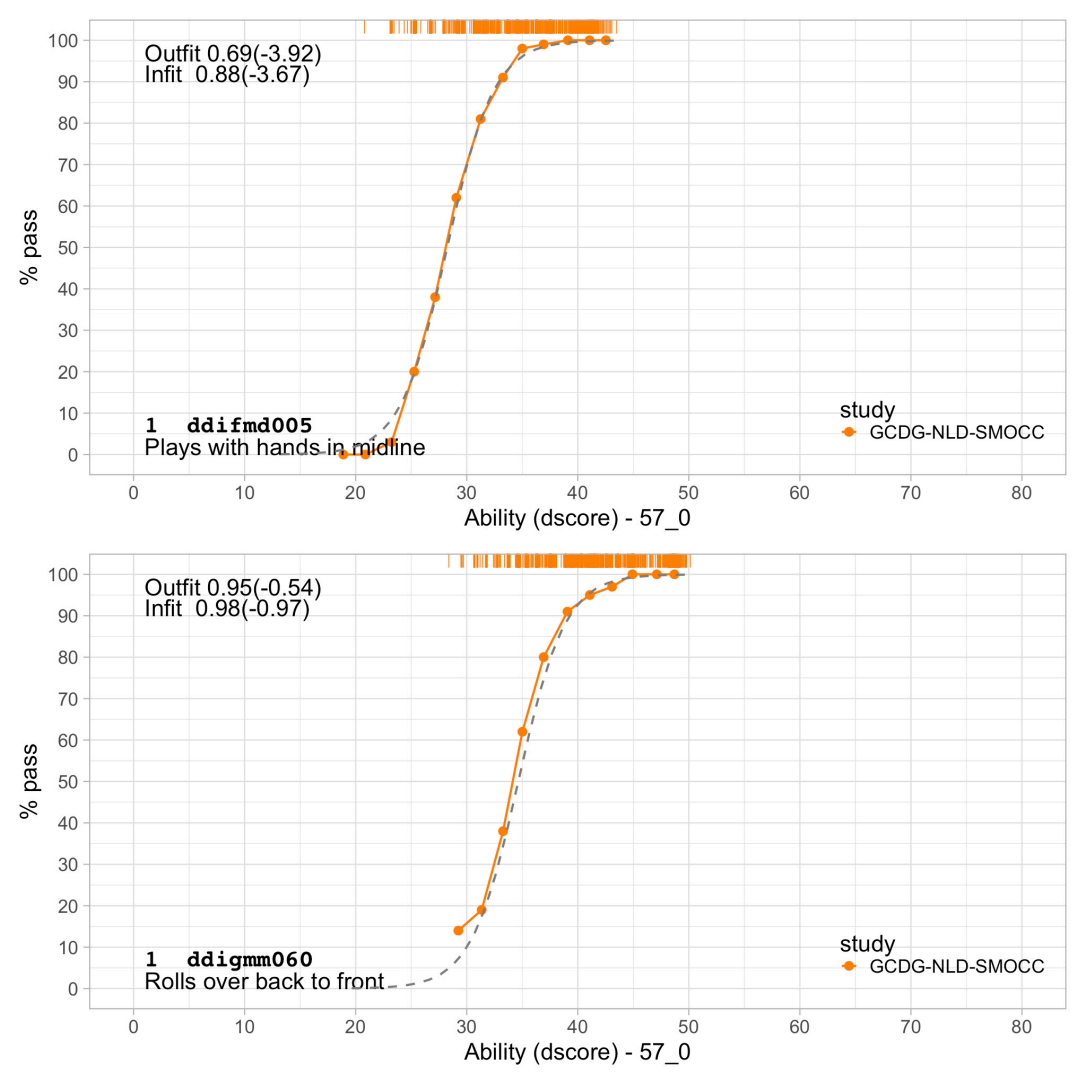
Empirical and fitted item response curves for two milestones from the DDI (SMOCC data).

***6.1.1 Well-fitting item response curves***. The study of *item fit* involves comparing the empirical and fitted probabilities at various levels of ability. Figure 6.1 shows the item characteristics curves of two DDI milestones. The orange line represents the empirical probability at different ability levels. The dashed line represents the estimated item response curve according to the Rasch model. The observed and estimated curves are close together, so both items fit the model very well.

***6.1.2 Item response curves showing severe underfit***. There are many cases where things are less bright.

**Figure 6.2.**
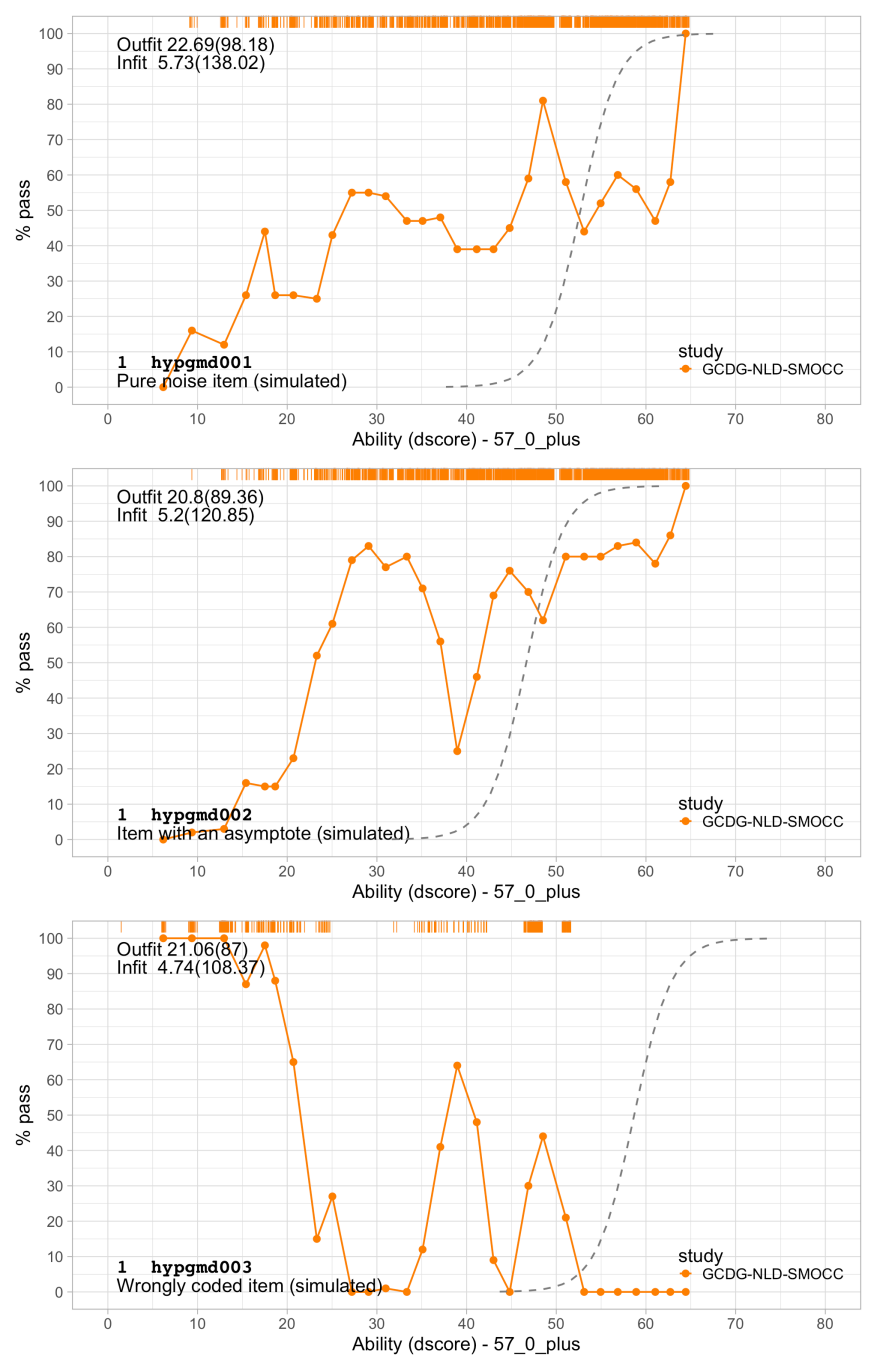
Three simulated items that illustrate various forms of item misfit.

Figure 6.2 shows three forms of severe underfit from three artificial items. These items were simulated to have a low fit, added to the DDI, and we estimated their parameters by the methods of Section 5. For the first item, hypgmd001, the probability of passing is almost constant across ability, so retaining this item essentially only adds to the noise. Item hypgmd002 converges to an asymptote around 80 per cent and has a severe dip in the middle. The strong relation to age causes the drop. Item hypgmd003 appears to have the wrong coding. Also, we often see the spike-like behaviour in the middle when two or more different items erroneously share identical names.

Removal of items with a low fit can substantially improve overall model fit.

**Figure 6.3.**
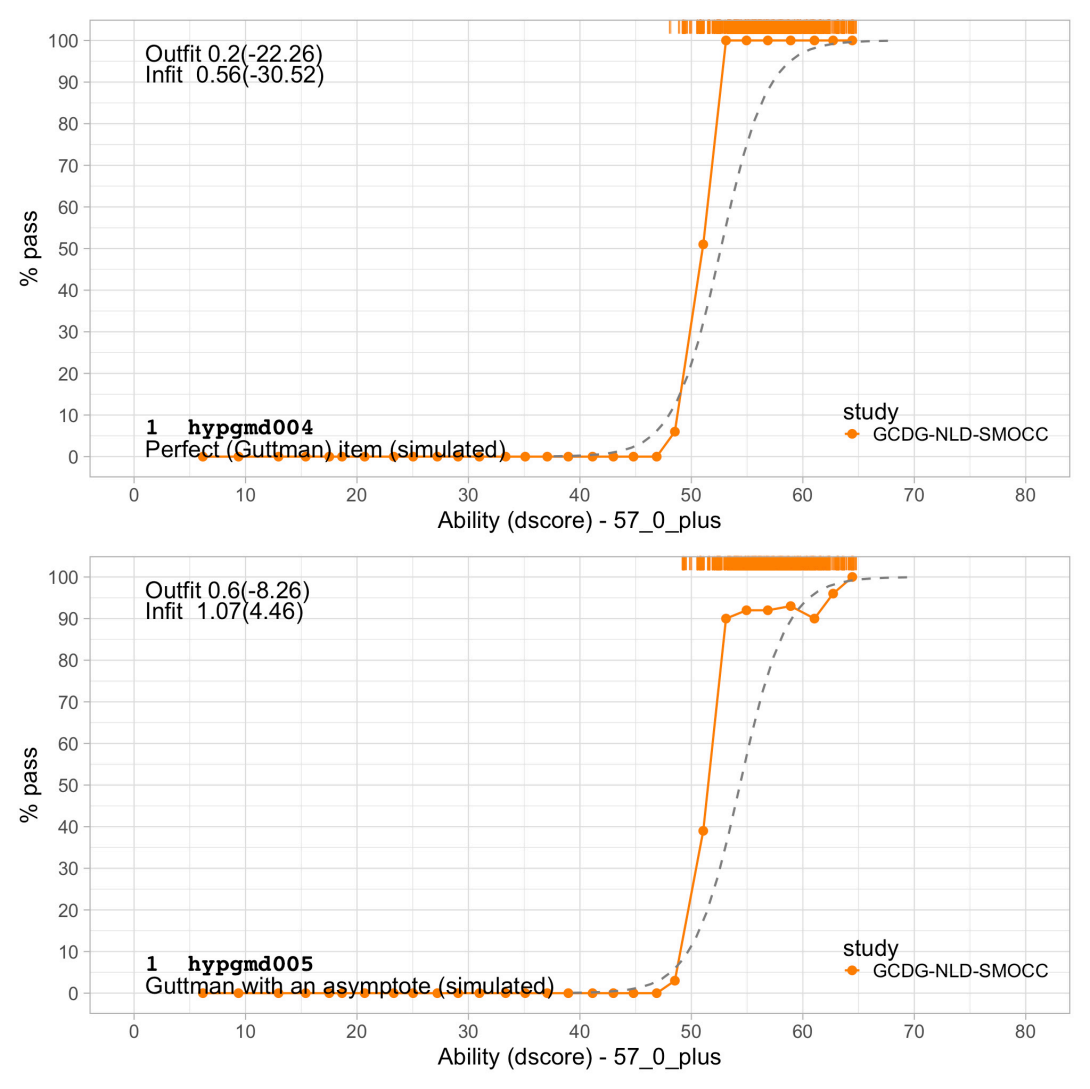
Two simulated items that illustrate item overfit.

***6.1.3 Item response curves showing overfit***. Figure 6.3 shows two artificial items with two forms of overfitting. The curve of item hypgmd004 is much steeper than the modelled curve. Thus, just this one item is exceptionally well-suited to distinguish children with a D-score below 50 *D* from those with a score above 50 *D*. Note that the item isn’t sensitive anywhere else on the scale. In general, having items like these is good news, because they allow us to increase the reliability of the instrument. One should make sure, though, that FAIL and PASS scores are all measured (not imputed) values.

Multiple perfect items could hint to a violation of the *local independence assumption* (c.f. Section 4.5). Developmental milestones sometimes have combinations of responses that are impossible. For example, one cannot walk without being able to stand, so we will not observe the inconsistent combination (stand: FAIL, walk: PASS). This impossibility leads to more consistent responses that would be expected by chance alone. In principle, one could combine the two such items into one three-category item, which effectively set the probability of inconsistent combinations to zero.

Item hypgmd005 is also steep, but has an asymptote around 80 per cent. This tail behaviour causes discrepancies between the empirical and modeled curves around the middle of the probability scale. In general, we may remove such items if a sufficient number of alternatives is available.

***6.1.4 Item infit and outfit***. We quantify item fit by item *infit* and *outfit*. Both are aggregates of the model residuals. The observed response *x _ni_* of person *n* on item *i* can be 0 or 1.

The *standardized residual* *z _ni_* is the difference between the observed response *x _ni_* and the expected response *p _ni_*, divided by the expected binomial standard deviation,

$$z_{ni}=\frac{x_{ni}-P_{ni}}{\sqrt{W_{ni}}},$$

where the expected response variance *W _ni_* is calculated as

$$W_{ni}=P_{ni}(1-P_{ni}).$$

*Item infit* is the total of the squared residuals divided by the sum of the expected response variances *W _ni_*

$$\text{Item}\;\text{infit}=\frac{\sum_{n}^{N}{\left(x_{ni}-P_{ni}\right)}^{2}}{\sum_{n}^{N}W_{ni}}.$$

*Item outfit* is calculated as the average (over *N* measurements) of the squared standardized residual

$$\text{Item}\;\text{outfit}=\frac{\sum_{n}^{N}z_{ni}^{2}}{N}.$$

The expected value of both infit and outfit is equal to 1.0. The interpretation is as follows:

•    If infit and outfit are 1.0, then the item perfectly fits the Rasch model, as in Figure 6.1;

•    If infit and outfit > 1.0, then the item is not fitting well. The amount of underfit is quantified by infit and outfit, as in 6.2;

•    If infit and outfit < 1.0, then the item fits the model better than expected (overfit). Overfitting is quantified by infit and outfit, as in 6.3.

Infit is more sensitive to disparities in the middle of the probability scale, whereas outfit is the better measure for discrepancies at probabilities close to 0 or 1. Lack of fit is generally easier to spot at the extremes. The two measures are highly correlated. Achieving good infit is more valuable than a high outfit.

Values near 1.0 are desirable. There is no cut and dried cut-off value for infit and outfit. In general, we want to remove underfitting items with infit or outfit values higher than, say, 1.3. Overfitting items (with values lower than 1.0) are not harmful. Preserving these items may help to increase the reliability of the scale. The cut-off chosen also depends on the number of available items. When there are many items to choose from, we could use a stricter criterion, say infit and outfit < 1.0 to select only the absolute best items.

**Figure 6.4.**
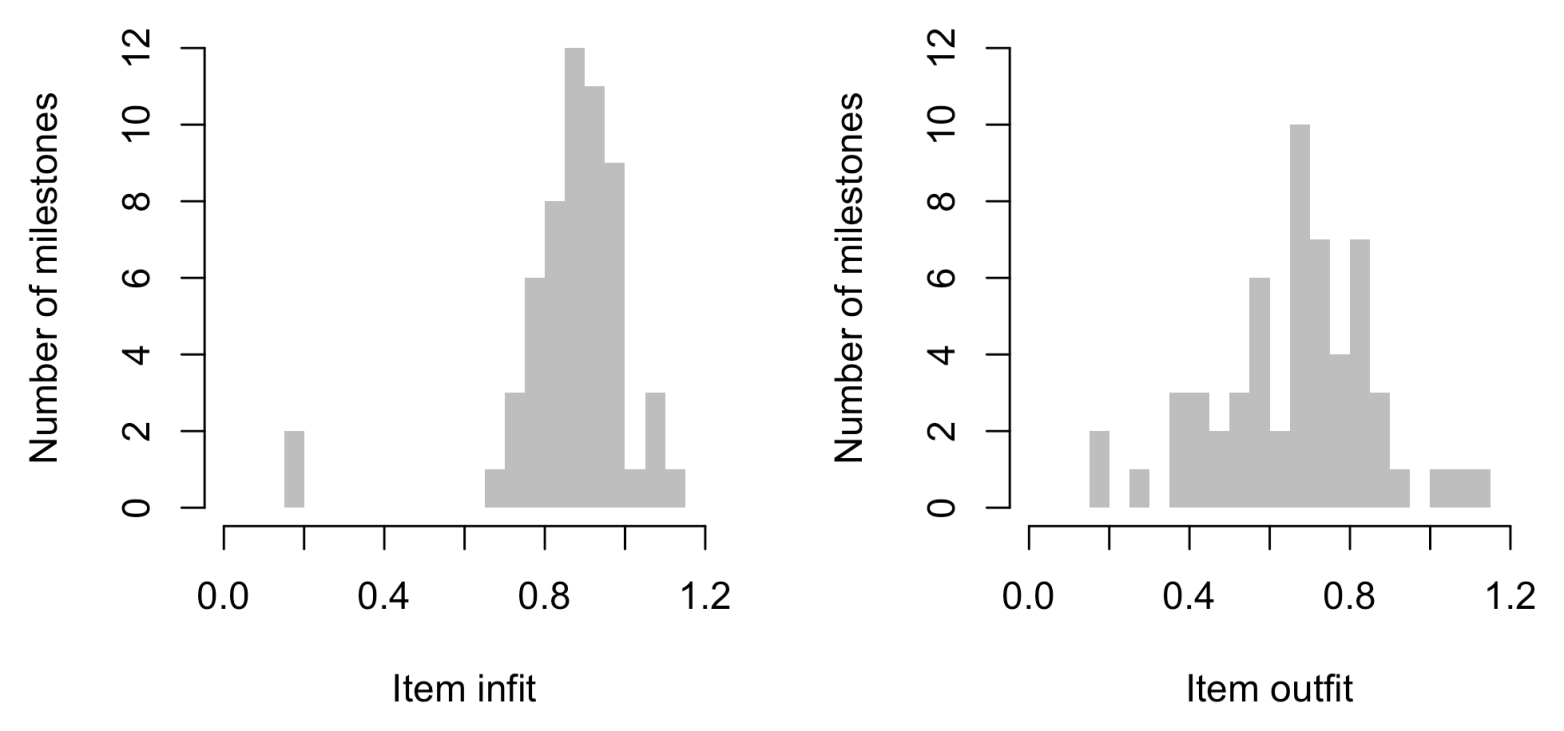
Frequency distribution of infit (left) and outfit (right) of 57 milestones from the DDI (SMOCC data).

***6.1.5 Infit and outfit in the DDI***. Figure 6.4 displays the histogram of the 57 milestones from the DDI (c.f. Section 4.1). Most infit values are within the range 0.6 - 1.1, thus indicating excellent fit. The two milestones with shallow infit values are ddigmd052 and ddigmd053. These two items screen for paralysis for newborns, so the data contain hardly any fails on these milestones. The outfit statistics also indicate a good fit.

### 6.2 Person fit

Person fit quantifies the extent to which the responses of a given child conform to the Rasch model expectation. The Rasch model expects that a more able child has a higher probability of passing an item than a less developed child. Person fit analysis evaluates the extent to which this is true.

***6.2.1 Person infit and outfit.*** In parallel to item fit, we can calculate *person infit* and *person outfit*. Both statistics evaluate how well the responses of the persons are consistent with the model. Outlying answers that do not fit the expected pattern increase the outfit statistic. The outfit is high, for example, when the child fails easy items but passes difficult ones. The infit is the information weighted fit and is more sensitive to inlaying, on-target, unexpected responses.

Similar to item fit, person fit is also calculated from the residuals, but aggregated differently. We calculate person infit as

$$\text{Person}\;\text{infit}=\frac{\sum_{i}^{L}{\left(x_{ni}-P_{ni}\right)}^{2}}{\sum_{i}^{L}W_{ni}}$$

and person outfit as

$$\text{Person}\;\text{outfit}=\frac{\sum_{i}^{L}z_{ni}^{2}}{L}$$

A threshold for person fit > 3.0 is customary to clean out children with implausible response patterns.

**Figure 6.5.**
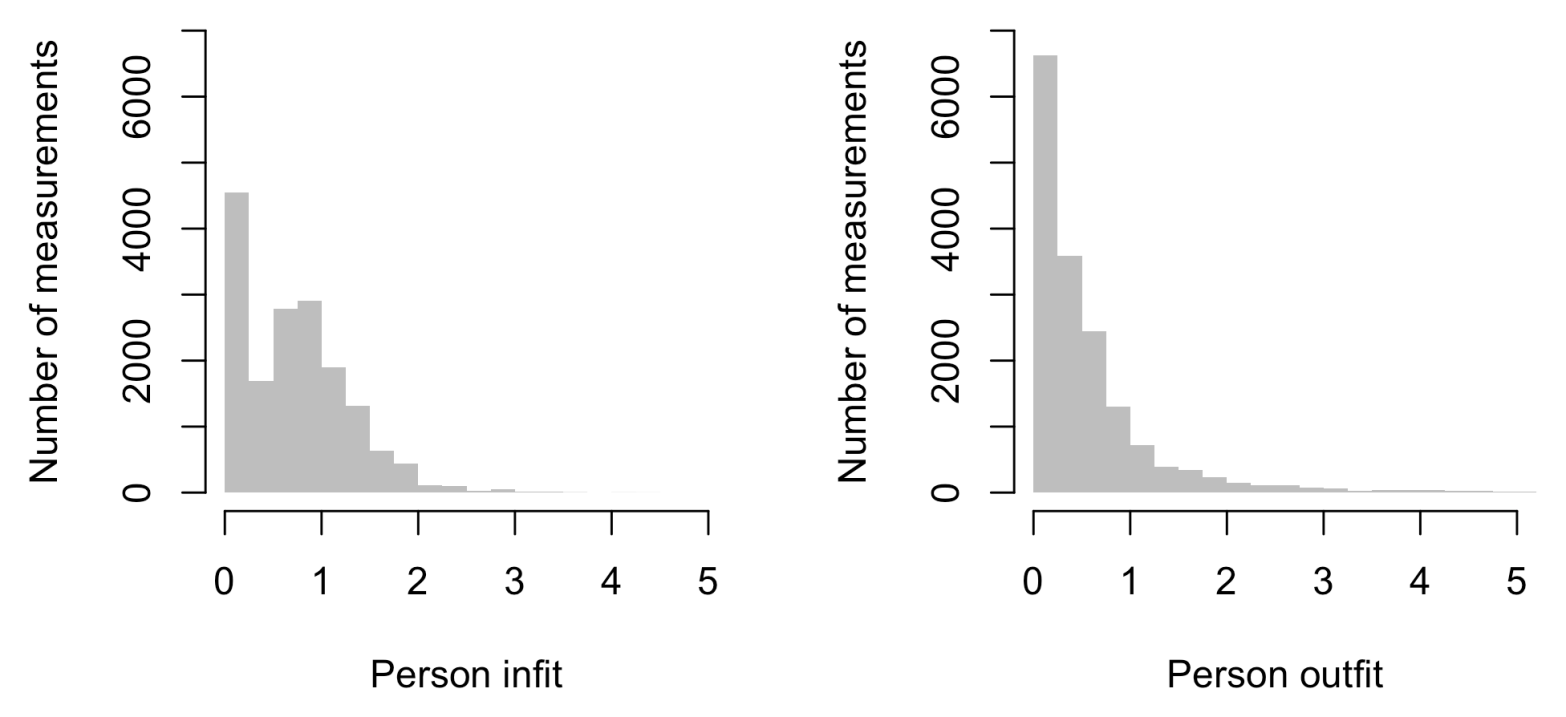
Frequency distribution of person infit (left) and person outfit (right) for 16538 measurements of the DDI (SMOCC data).

***6.2.2 Person infit and outfit in the DDI.*** Figure 6.5 displays the frequency distribution of person infit and person outfit 16538 measurements of the DDI in the SMOCC data. The majority of the values falls below 3.0. For infit, only 43 out of 16538 fit values (0.3 per cent) is above 3.0. There are 446 out of 16538 outfit value (2.7 per cent) above 3.0. Expect the solution to improve after deleting these measurements.

### 6.3 Differential item functioning (DIF)

***6.3.1 Relevance of DIF for cross-cultural equivalence.*** An essential assumption in the Rasch model is that a given item has the same difficulty in different subgroups of respondents. Climbing stairs is an example where this assumption is suspect. The exposure to stairs, and hence the opportunity for a child to practice, varies across different cultures. It could thus be that two children with the same ability but from different cultures have different success probabilities for climbing stairs. When these probabilities systematically vary between subgroup, we say there is *Differential Item Functioning*, or *DIF* ( Holland & Wainer, 1983). DIF is undesirable since it can make the instrument culturally biased.

***6.3.2 How to detect DIF?***

Zumbo (1999) provided a clear definition of DIF:

DIF occurs when examinees from different groups show differing probabilities of success on (or endorsing) the item after matching on the underlying ability that the item is intended to measure.

There are various ways to detect DIF. Here we will model the probability of endorsing an item by logistic regression using the observed item responses as the outcome. Predictors include the ability, the grouping variable, and the ability-grouping interaction. If the latter two terms explain the residual variance of the item scores after adjusting for ability, the item shows DIF for that group. DIF can be visually inspected by plotting the curves for the subgroups separately.

There are two forms of DIF:

• *Uniform DIF*: The item response curves differ between groups in location, but are parallel;

• *Non-uniform DIF*: The item response curve differ between groups in location, in slope and possibly in other characteristics.

These forms correspond to, respectively, the main effect of group and the ability-group interaction in the logistic regression model.

**Figure 6.6.**
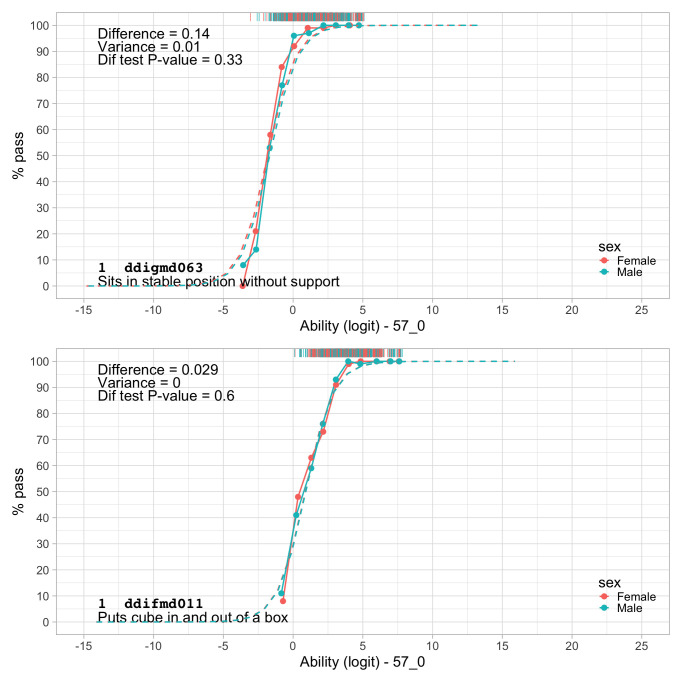
Two milestones from the DDI with similar item response curves for boys and girls. There is no DIF for sex.

***6.3.3 Examples of DIF.*** Figure 6.6 shows an example comparing boys and girls. For both milestones, the item response curves are similar for boys and girls, so we see no evidence of DIF here.

**Figure 6.7.**
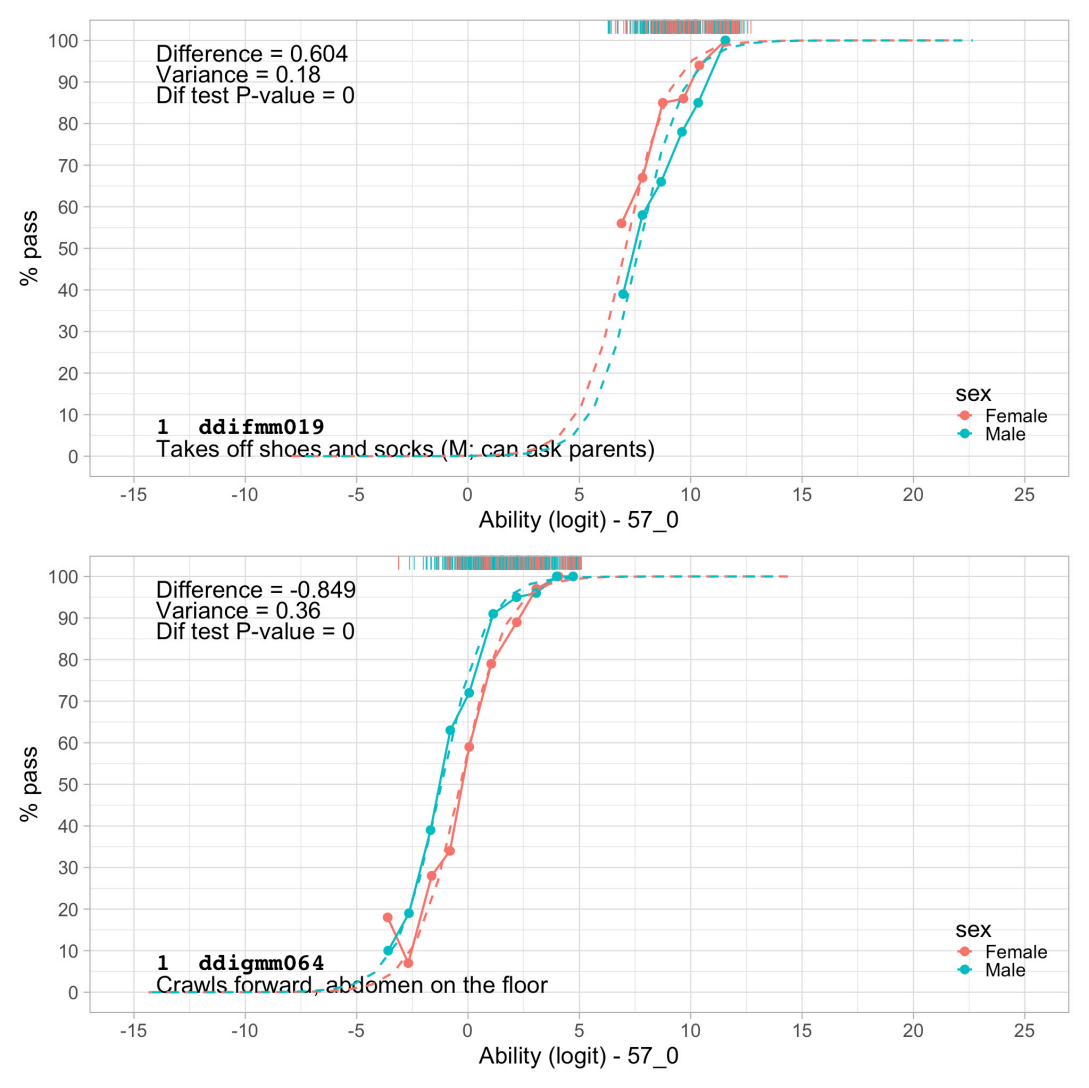
Two milestones from the DDI with different item response curves for boys and girls. There is evidence for uniform DIF.

Figure 6.7 displays two milestones with DIF between boys and girls. Provided that the ability estimate (as estimated from all items in the model) is fair for both boys and girls, we see that milestone ddifmm019 (“Takes off shoes and socks”) is easier for girls by about 0.86 logits (= the difference in ability at the intersection of 50 per cent). Conversely, milestone ddigmm064 (“Crawls forward, abdomen on the floor”) is easier for boys by about 0.84 logits. These are the most substantial differences found for sex in the DDI. Both are uniform DIF.

In practice, having milestones with opposite directions of DIF in the same instrument will cancel out one another, so one need not be overly concerned in that case. However, we should be careful when the tool consists of milestones that all have DIF in the same direction.

The DDI did not contain items for which the ability-group interaction was statistically significant, so we conclude that there is no non-uniform DIF in the DDI.

### 6.4 Item information

***6.4.1 Item information at a given ability.*** Items are generally sensitive to only a part of the ability scale. Item information is a psychometric measure that quantifies how illuminating the item is at different levels of ability. We may visualize item information as a curve per item.

The formula to obtain the item information is the first derivative of the item response curve and can be written as follows:

$$I({\hat{\delta }}_{i})=P({\hat{\delta }}_{i})(1-P({\hat{\delta }}_{i}))$$

where $P({\hat{\delta }}_{i})$ is the conditional probability of endorsing item *i*, and where ${\hat{\delta }}_{i}$ is the estimated item difficulty in the logit scale. For example for milestone ddicmm039 (“Says three words”) ${\hat{\delta }}_{i}$ equals 4.06.

**Figure 6.8.**
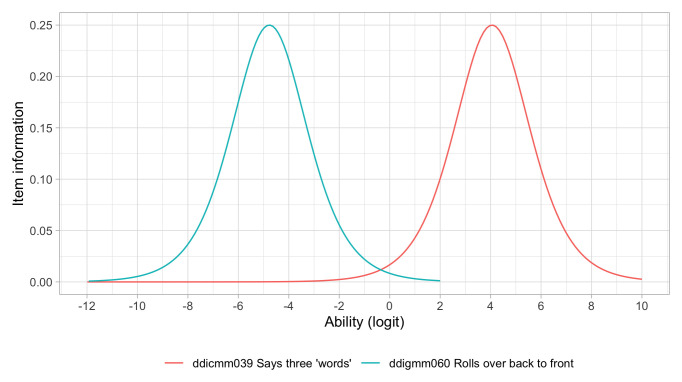
The item information curve for two milestones from the DDI.

Figure 6.8 displays the item information curves for two milestones from the DDI. Note that the amount of information for the item is maximal around the item difficulty.

The probability of endorsing milestone ddicmm039 for a child with an ability of 2 logits is

$$P_{ni}=\frac{\exp(2-4.06)}{1+\exp(2-4.06)}=0.113$$

At this ability level, milestone ddicmm039 has information

$$I({\hat{\delta }}_{i})=0.113\times (1-0.113)=0.10$$

***6.4.2 Item information at a given age.*** In practice, it is often interesting to express the item information against age. By doing so, one can identify at what ages an item provides the most information.

**Figure 6.9.**
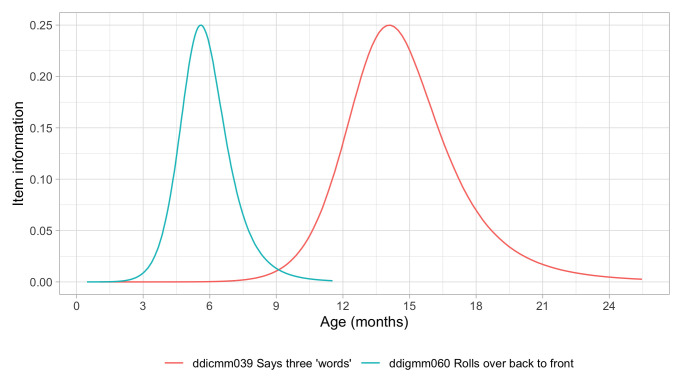
Information information of Figure 6.8 plotted against age.

Figure 6.9 shows that the sensitive age ranges differ considerably between items. Suppose we use 0.05 as a criterion. Then ddigmm060 is susceptible between ages 4–8 months, a period of four months. Item ddicmm039 is receptive in the period 10–19 months, a range that is about twice as broad. The symmetric nature of the curves in Figure 6.8 is not present in Figure 6.9. In general, the relation between age and item sensitivity is more complicated than the relationship between ability and item sensitivity.

The item information by age curve helps to determine at what ages we should administer the item. The item will be most informative if delivered at the age at which 50% of the children will pass the milestone. This age corresponds to an item information is equal to 0.5 * 0.5 = 0.25. Administering the item closely around that age provide the most efficient measurement of ability. When space is at a premium (e.g. as in population surveys) using a well-chosen set of age-sensitive milestones will help in reducing the total number of milestones.

In other contexts, milestones may be used as a screening instrument to identify developmental delay. In that case, it is more efficient to administer items that are very easy for the age, e.g. milestones on which, say, 90% of the children will pass.

### 6.5 Reliability

The reliability is a one-number summary of the accuracy of an instrument. Statisticians define reliability as the proportion of variance attributable to the variation between children’s abilities relative to the total variance. More specifically, the reliability *R* of a test is written as

$$R\equiv \frac{\sigma_{\beta }^{2}}{\sigma_{\beta }^{2}+\sigma_{e}^{2}},$$

where $\sigma_{\beta }^{2}$ is the variance of true scores and $\sigma_{e}^{2}$ is the error variance.

In general, high reliability is desirable. We often use reliability to decide between instruments. Cronbach’s *α* is a widely used estimate of the lower bound of the reliability of a test. In the Rasch model, we can estimate reliability by the ratio

$$\overset{=}{R}\equiv \frac{{\hat{\sigma }}_{\hat{\beta }}^{2}-{\hat{\sigma }}_{\hat{e}}^{2}}{{\hat{\sigma }}_{\hat{\beta }}^{2}}.$$

For a given model, we can calculate ${\hat{\sigma }}_{\hat{\beta }}^{2}$ directly as the sampling variance of the estimated abilities. Getting an estimate for ${\hat{\sigma }}_{\hat{e}}^{2}$ is more complicated. We use the modelled person abilities and item difficulties to generate a hypothetical data set of the same size and same missing data pattern, and re-estimate the person ability from the simulated data. Then ${\hat{\sigma }}_{\hat{e}}^{2}$ is computable as the variance of the difference between the modelled and re-estimated person ability.

The estimated variance of the modeled abilities is ${\hat{\sigma }}_{\hat{\beta }}^{2}$ = 76.6, and the variance of the difference between modeled and re-estimated abilities is equal to ${\hat{\sigma }}_{\hat{e}}^{2}$ = 1.74. The corresponding *standard error of measurement (sem)* is ${\hat{\sigma }}_{\hat{e}}$ = 1.32 logits.

The estimated reliability in the SMOCC data is equal to (76.6 – 1.74)/76.6 = 0.977. We may interpret this estimate in the same way as Cronbach’s *α*, for which typically any value beyond 0.9 is classified as *excellent*. Note that the reliability is very high because of the large variation in D-scores. Newborns are very different from 2-year old toddlers, which helps to increase reliability. In practice, it is perhaps more useful to use a measure of accuracy that is less dependent on the variation within the sample. The *sem*, as explained above, seems to be a more relevant measure of precision.

## 7 Validity

Validity is a generic term that refers to the question of how well an instrument measures what it claims to measure. There are various aspects of validity. This section briefly reviews the main types of validity:

•      Internal validity (7.1)

•      External validity (7.2)

### 7.1 Internal validity

***7.1.1 Content validity.*** Content validity is the extent to which the D-score represents all facets of development. In contrast to “face validity,” which assesses whether the test appears valid to respondents, content validity is about what is measured.

One important form of content validity is that we wish to make sure that the measurement scale represents the various developmental domains in a fair way. In the simplest case, we can assign each milestone uniquely to one domain and evaluate coverage by splitting the cumulative item information.

**Figure 7.1.**
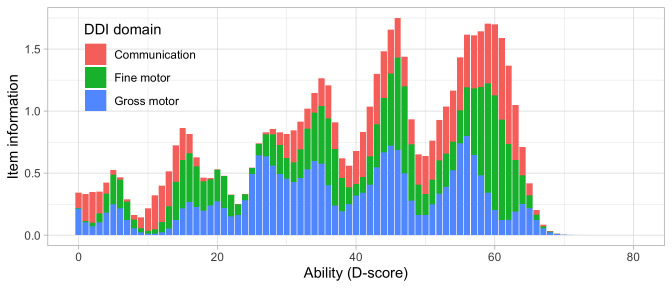
Cumulative item information by DDI domain.

Figure 7.1 shows the coverage of the three domains of the DDI at various levels of the D-score. The three domains of the DDI are relevant at most ability levels. The DDI contains no communication milestones between 20 *D* and 30 *D*, so at these levels, the DDI measures primarily motor performance.

***7.1.2 Construct validity.*** Construct validity is the extent to which the D-score behaves like the theory says the construct should behave. For example, we expect that child development advances with age. Figure 4.3 provides convincing evidence that the D-score increases fastest in the first six months and keeps rising at a slower rate as children age. This phenomenon is consistent with theories in growth and child development.

**Figure 7.2.**
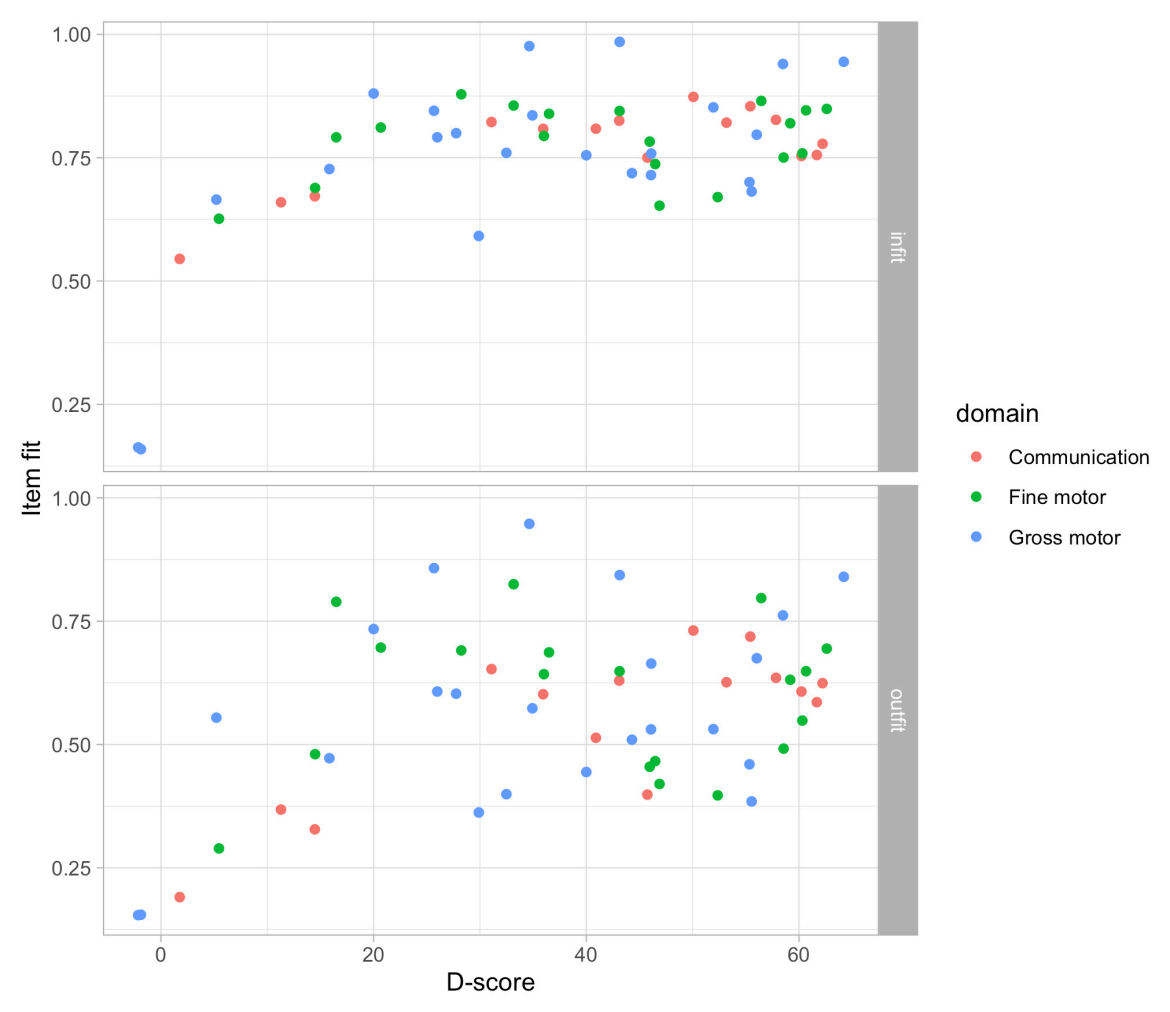
Item fit by D-score for the DDI domains.

In Section 4, we assumed that child development is a latent variable. Figure 7.2 provides one way to evaluate the validity of this assumption. The figure plots the item fit for each milestone coloured by domain. Items from different domains fit equally well, so there is no evidence that the D-score favours a particular area. Put in more technical terms; the DDI domains do not explain differences in the item fit residuals of the model.

### 7.2 External validity

***7.2.1 Discriminatory validity.*** Discriminatory validity indicates the extent to which the D-score can distinguish children with non-normal development from children that are developing normally. We may evaluate this by identifying children with lagging development, for example, indicated by reflex or tonus problems, and study whether the D-score can discriminate those children from the general population. Section 9.3 presents some examples.

***7.2.2 Convergent and divergent validity.*** Convergent validity is the extent to which the D-score relates to similar constructs. We measure it by the correlation between the D-score and the total score on Bayley-III or Denver.

The correlation with the other construct should be 0.6, or higher for good convergent validity. Unfortunately, at present, only limited data is available for the DDI, so we cannot assess convergent validity for the D-score at this point.

Divergent validity is the extent to the D-score is uncorrelated with measures of a different construct.

**Figure 7.3.**
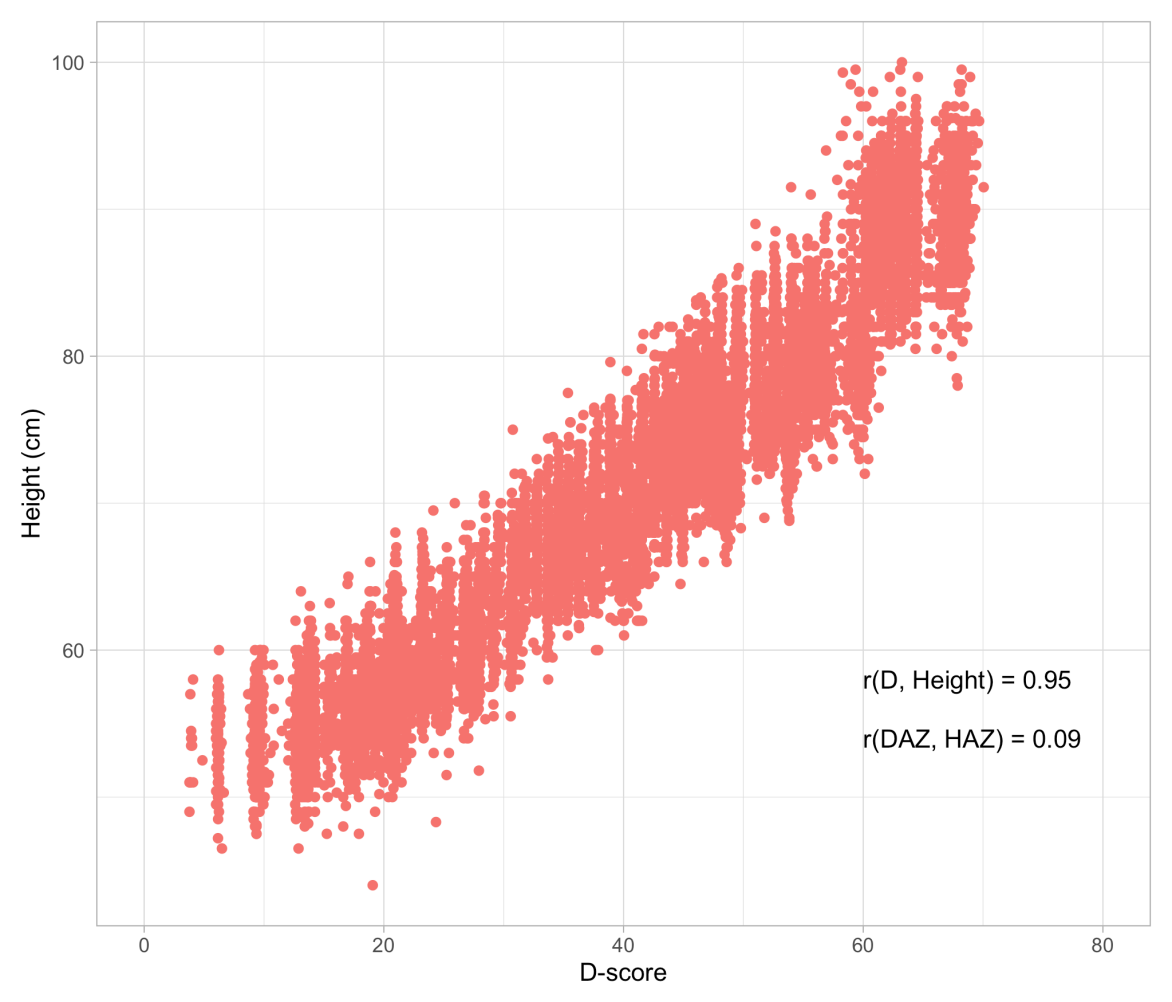
Relation between body height and the D-score in the SMOCC data.

Figure 7.3 shows both convergent and divergent validity at work. The figure shows that, as expected, there is a strong and almost linear relation between body height and the D-score. However, after correction for the child’s age, the relationship between height and D-score almost disappears. Thus, growth and development are entirely different concepts.

We can also evaluate the strength of the relations between the D-score and proxy measures of child development, such as stunted height growth (see section 1.3). The low correlation between DAZ and HAZ suggests that stunting is a poor proxy for child development.

***7.2.3 Predictive validity.*** Predictive validity refers to the degree to which the D-score predicts the score on a criterion that is measured later. For the D-score, we may compare to measures for IQ at the school-age as a possible criterion.

Vlasblom *et al.* (2019) found strong evidence that individual milestones of the DDI measured during the first years of life predict later intellectual functioning at ages 5–10 years. It is to be expected that the D-score, which builds upon these individual items, will also predict limited intellectual functioning, perhaps even better.

## 8 Precision

This section shows the properties of the D-score when calculated from short tests. The study of quick tests is useful because it reveals the behaviour of the D-score when the measurement is inherently imprecise.

This section covers:

•      Structure of milestone subsets (8.1)

•      Impact of short tests on D-score (8.2)

•      Impact of short tests on predicting IQ (8.3)

### 8.1 SMOCC design: Standard and additional milestones

At each visit, the SMOCC study collected scores on a set of *standard milestones* (that about 90 per cent of the children will pass) and a set of *additional milestones* (that about 50 per cent of the children will pass). See Section 4.1.2.

The SMOCC dataset covers nine different *waves*. The set of milestones used in the DDI varies per visit. The number of standard milestones varies between 2 and 7 on various occasions. The additional milestones equal the standard ones from the next wave.

Table 8.1 summarizes the scheduled age for each wave, the number of standard milestones and the number of additional milestones.

**Table 8.1.** Number of items administered per wave in the SMOCC data.

Age	Standard	Additional
1m	5	2
2m	2	5
3m	5	6
6m	6	7
9m	7	6
12m	6	6
15m	6	6
18m	6	7
24m	7	7

**Figure 8.1.**
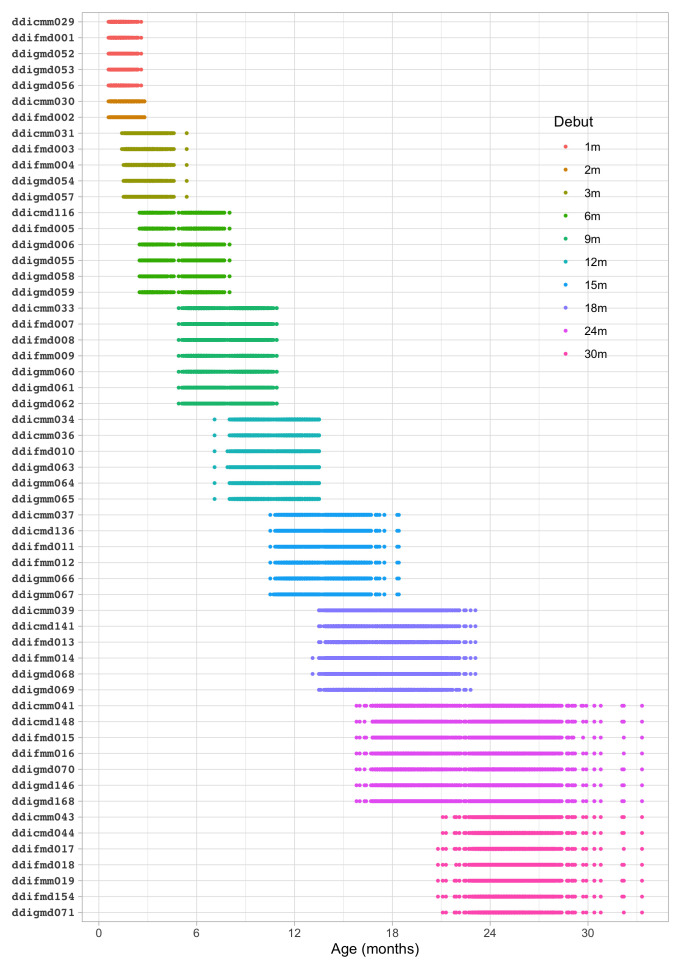
Age-item grid of the SMOCC study, illustrating how the 57 DDI items are distributed over nine visits during the first 24 months.

Figure 8.1 shows the subsets of DDI items administered at each age. For example, at the 1-month visit, the five standard milestones are ddicmm029 - ddigm056, and the two additional ones are ddicmm030 and ddifmd002. At the 2-month visit, the standard milestones are ddicmm030 and ddifmd002, and the five additional ones are ddicmm031 - ddigmd057. And so on.

### 8.2 D-score from short tests

***8.2.1 Milestone sets.*** In the analyses done thus far, we have calculated D-scores from responses on the combined (standard plus additional) milestones. Thus, at the 2-month visit, the D-score was calculated from 2 (standard) + 5 (additional) = 7 milestones.

In daily practice, the set of additional milestones is often lacking. This section explores the impact of using the (smaller) subset of standard milestones on measurement error and prediction.

This section reports and compares three D-scores:

1.      D-score from standard milestones;

2.      D-score from additional milestones.

3.      D-score from all available milestones;

Estimation of 1 is more complicated than for 2 and 3, for the following reasons:

•      There are fewer milestones, so the estimate is less precise and more influenced by choice of the prior distribution;

•      The standard set contains only easy milestones, which are uninformative for the majority of children.

**Figure 8.2.**
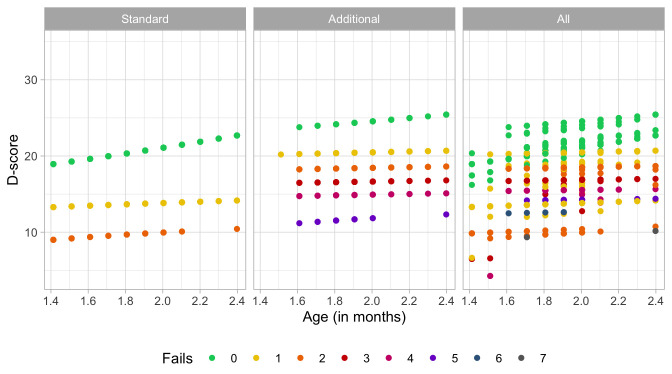
Distribution of the D-scores calculated from the standard, additional and all available milestones at month 2. Colors correspond to the number of fails.

***8.2.2 Milestone sets at month 2.*** The vertical axis of Figure 8.2 shows the D-score, separately calculated from the standard, additional and all milestones for children aged two months. The colour of the dots represents the number of FAIL ratings within each set of milestones.

At month two there are just two standard milestones: ddicmm030 and ddifmd002. About 90 per cent of the infants will pass these. The green dots in the left-hand side figure represent the estimated D-scores corresponding to two passes. As explained in Section 5.3.2, we calculate the D-score with an age-dependent prior. If the ages vary (and they do), then the D-score for infants having the same total score will also vary.

If a child fails either ddicmm030 or ddifmd002, then the D-score is substantially lower. The left-hand figure shows a *gap* between the green dots (perfect score) and the yellow dots (one FAIL). The impact of a FAIL on the D-score is substantial. For example, the D-score of an infant with one FAIL on a standard milestone drops from about 20 *D* to 14 *D*. Thus, with these two milestones, there cannot be a D-score in the range 15 *D* - 18 *D*. It depends on the purposes of the measurement if this is acceptable. We can prevent gaps by measuring more milestones, e.g., milestones taken from the additional set. Another gap occurs between 14 *D* and 11 *D*. These gaps illustrate that precision is constrained if we administer only two milestones.

The middle figure shows the estimated D-score at the same visit but now calculated from the five additional milestones (i.e., the standard milestones from month 3). Infant aged two months have approximately a 50 per cent chance of passing each. Note that administration of the additional milestones will cover the range 14D-20 *D* quite well. Note the ceiling is also higher with these milestones.

Note that the range of the estimated D-scores is quite similar in both plots. This similarity is a result of accounting for the difficulty level of milestones. The estimate of the D-score is *unbiased* for difficulty.

The figure on the right-hand side provides the D-score calculated from all milestones. We can easily recognise the points coming from the standard and additional sets. Also, there is a limited number of ratings on easier items that belong to month 1. We rescored these because the child failed these milestones at the previous visit. Rescoring effectively extends the range of possible D-scores to the lower end, so now we can find some children who have D-score lower than 10 *D*.

**Figure 8.3.**
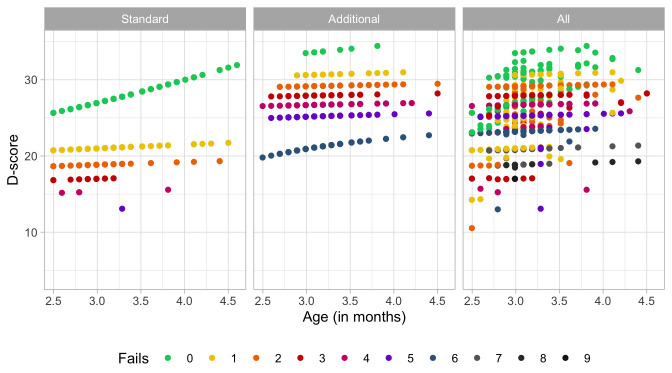
Distribution of the D-scores calculated from the standard, additional and all available milestones at month 3. Colors correspond to the number of fails.

***8.2.3 Milestone sets at month 3.*** Figure 8.3 is the same plot as before, but now for month 3. Compared to Figure 8.2, all points shifted upwards because the children are now one month older.

The additional milestones from month 2 are the standard milestones of month 3. In Figure 8.2, there were at least 11 children (in purple) failed all five additional milestones. One month later, one child has five fails.

**Figure 8.4.**
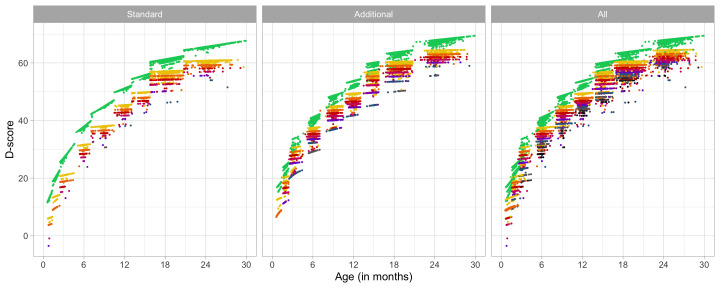
D-score by age 0–30 months for standard, additional and all available milestones at each measurement occasion.

***8.2.4 Floor and ceiling effects.*** Figure 8.4 plot the D-score distribution for all occasions. Some observations:

• *Ceiling effect*: The ceiling effect (green) is most prominent in the *standard* set, but is also present in the other two sets. None of the three sets can filter out children with really advanced development. To achieve more precision at the upper end, we would need to include more difficult milestones.

• *Floor effect*: There are almost no floor effects in the *standard* and *all* sets. These sets discriminate well among children with delayed development, which was the designed purpose of the DDI. Note that floor effects are visible in the *additional* set.

• *Average level*: All three sets capture the overall relation between age and development. The *additional* set is quite efficient for measuring average levels development but lacks detail on the extremes.

Figure 8.4 shows that a short test (5–6 milestones) can precisely measure the lower tail of the D-score distribution ( *standard* set) or the middle of the D-score distribution ( *additional* set), but cannot do both at the same time.

### 8.3 Impact of short tests on predicting IQ

***8.3.1 Measurement and prediction.*** In Section 8.2, we saw that a short test can measure the middle or one tail of the distribution, but cannot be precise for both at the same time. If we want to identify children at risk for delayed development, we are interested in the lower tail of the distribution, so in that case, the *standard* set is suitable. But what set should we use if we want to predict a later outcome?

This section explores that effect of taking different milestone sets on the quality of prediction.

***8.3.2 UKKI.*** Hafkamp-de Groen *et al.* (2009) studied the effect of the D-score on later intelligence, using a subset of 557 SMOCC children that were followed up at the age of five years.

The Utrechtse Korte Kleuter Intelligentietest (UKKI) ( Baarda, 1978) is a short test to measure intelligence. The UKKI is a simple test with just three components:

•      Redraw five figures (square, triangle, cross, trapezoid, rhomboid);

•      Draw human figure, with 28 characteristics, like legs, eyes, and so on;

•      Give meaning to 13 words like knife, banana, umbrella, and so on.

Administration time is about 15–20 minutes. The UKKI has a reasonable test-retest reliability for group use (Pearson *r* = 0.74, 3-month interval).

**Figure 8.5.**
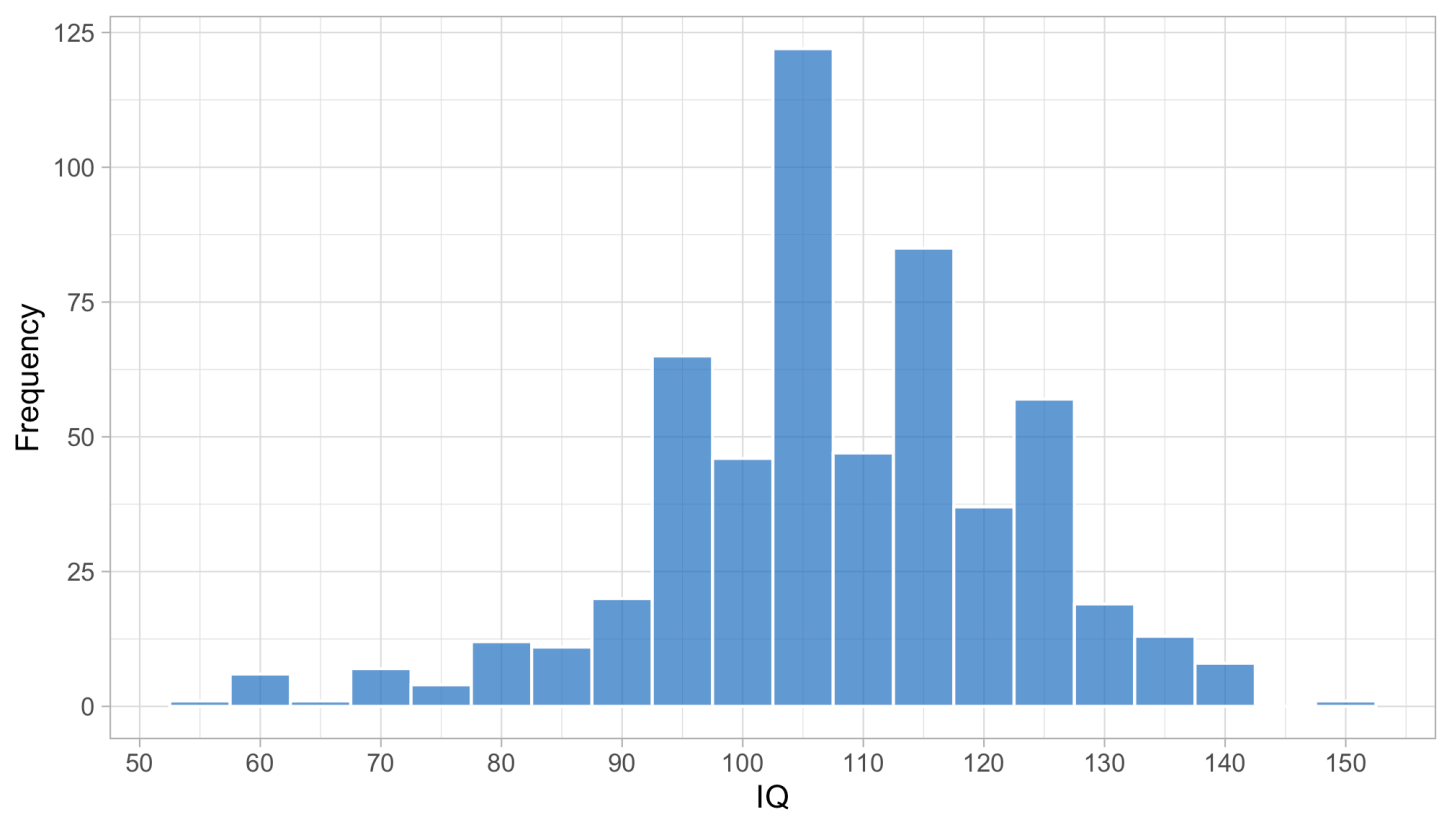
Histogram of UKKI *IQ* scores taken around the age of five years (SMOCC data, *n* = 557).

***8.3.3 Exploratory analysis.*** Figure 8.5 shows the empirical IQ distribution of 557 children. The mean IQ score is 108, and the standard deviation is 15, so the IQ-scores of children in the sample is about a half standard deviation above the 1978 reference sample.

**Figure 8.6.**
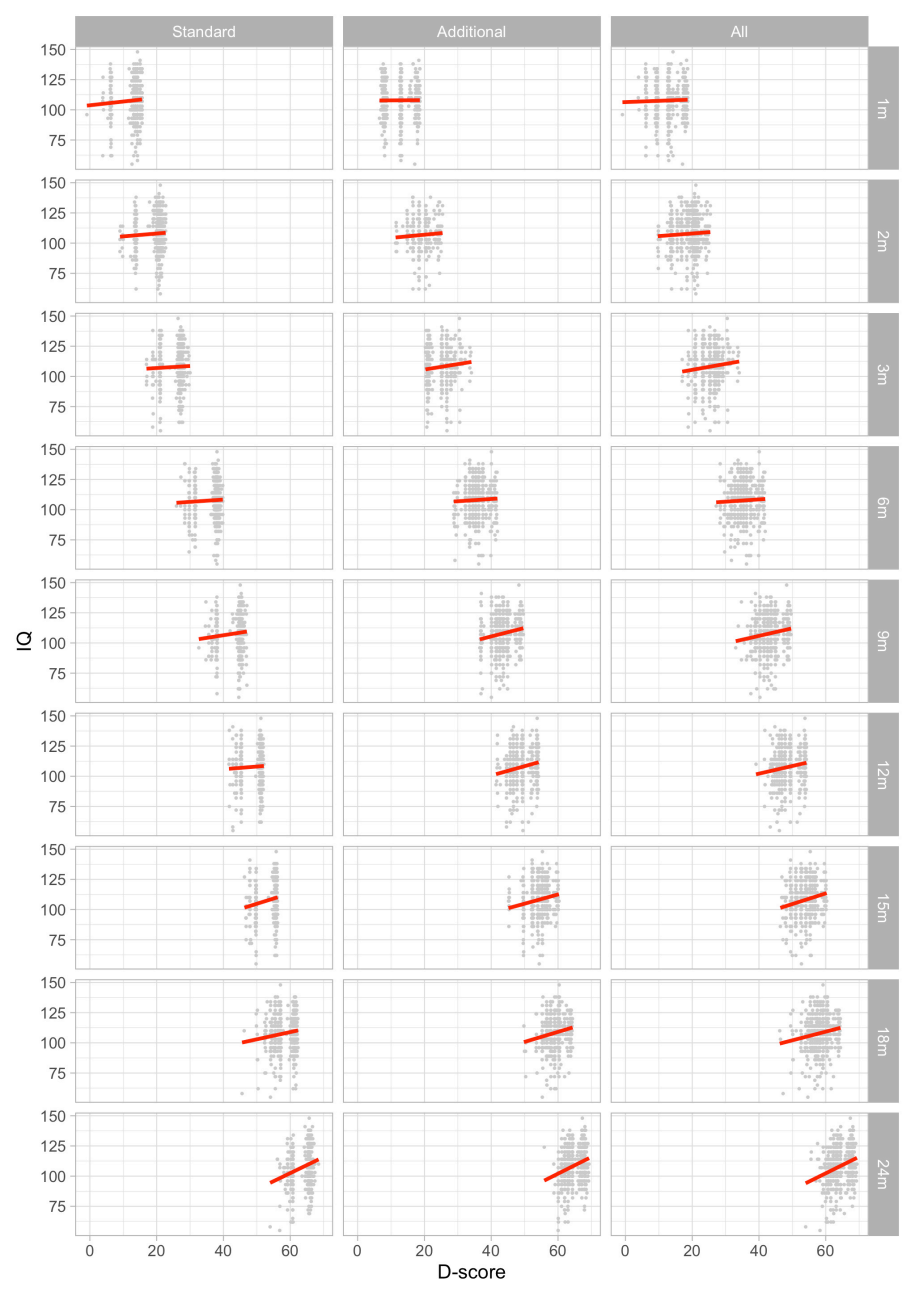
Relation between D-score at infancy and *IQ* at age 5 years according to three milestone sets and nine visits (SMOCC data, *n* = 557).

Figure 8.6 shows that the relation between the D-score 0–2 years and IQ at five years is positive for all milestone sets and all ages. The strength of the association increases with age. At the age of 2 years, the regression coefficient for D-score is equal to *β* ( *D*) = 1.4 (SE: 0.21, *p* < 0.0001), so on average an increase of 1.0 unit in the D-score at the age of 2 years corresponds to a 1.4 IQ-score points increase at the age five years.

Table 8.2 summarizes the Pearson correlations between the D-score and later IQ. The association between D-score and IQ is weak during the first year of life but gets stronger during the second year. In general, having more (and more informative) milestones helps to increase the correlation, but the effects are relatively small. So even from the standard set of the seven easy milestones at 24m, we obtain a reasonable correlation of 0.245.

**Table 8.2.** Pearson correlation between D-score (0–2 years) and IQ at 5 years.

Visit	Standard set	Additional set	All milestones
1m	0.059	0.005	0.027
2m	0.051	0.056	0.048
3m	0.036	0.100	0.102
6m	0.040	0.038	0.036
9m	0.094	0.143	0.132
12m	0.046	0.162	0.137
15m	0.180	0.153	0.187
18m	0.129	0.153	0.146
24m	0.245	0.255	0.267

All in all, these results suggest that neither the amount nor the difficulty level of the milestones is critical in determining the strength of the relation between the D-score and IQ.

## 9 Three studies

This section compares child development between samples from three different studies:

• *SMOCC*, a representative sample of Dutch children (9.1)

• *POPS*, a cohort of all Dutch preterms in 1983 (9.2)

• *TOGO*, a set of medical records from preventive health service in Togo (9.3)

•      A summary of the main findings (9.4)

Each study used the same measurement instrument, the DDI (see Section 4.1). The section compares D-scores between studies.

### 9.1 SMOCC study

**Figure 9.1.**
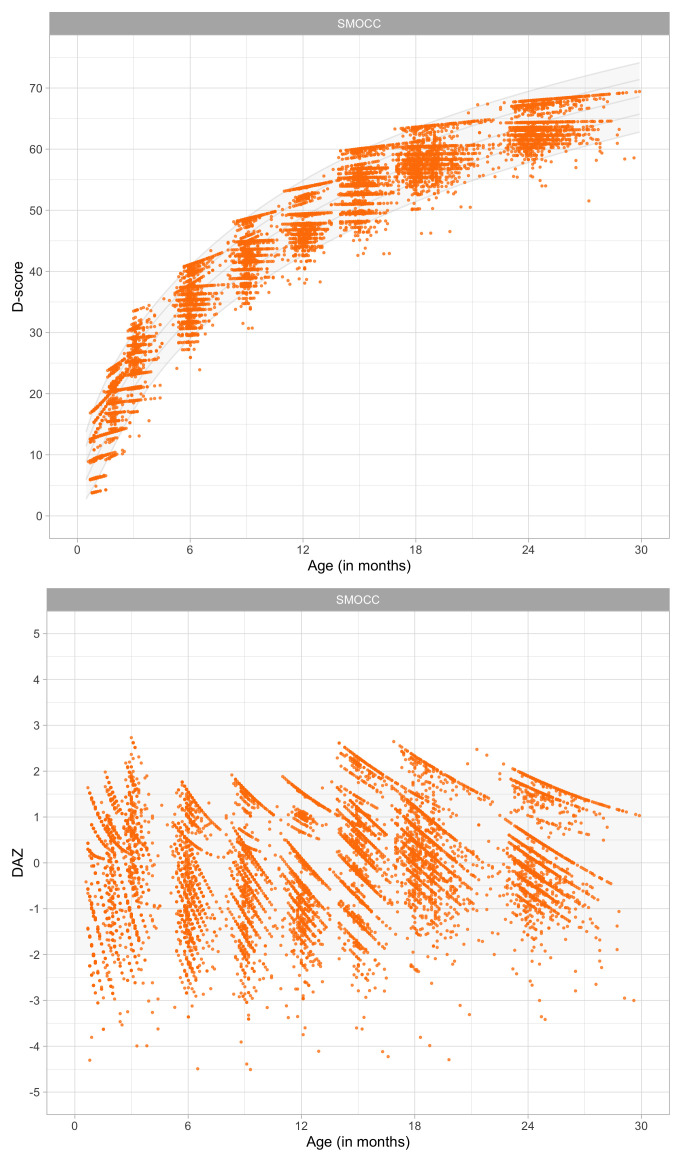
Distribution of D-score and DAZ by child age in a cohort of Dutch children aged 0–2 years (Source: SMOCC data, *n* = 2151, 9 occasions).

Figure 9.1 shows the D-score distribution by age in the SMOCC data. The grey curves represent references calculated from the SMOCC data. The top figure illustrates that rise of the D-score with age, whereas the bottom chart shows that the DAZ distribution covers the references well.

The ceiling effect causes low coverage after the age of 24 months. There are also less prominent ceiling effects for younger children. Without these effects, the references would presumably show some additional variation.

### 9.2 POPS study

**Figure 9.2.**
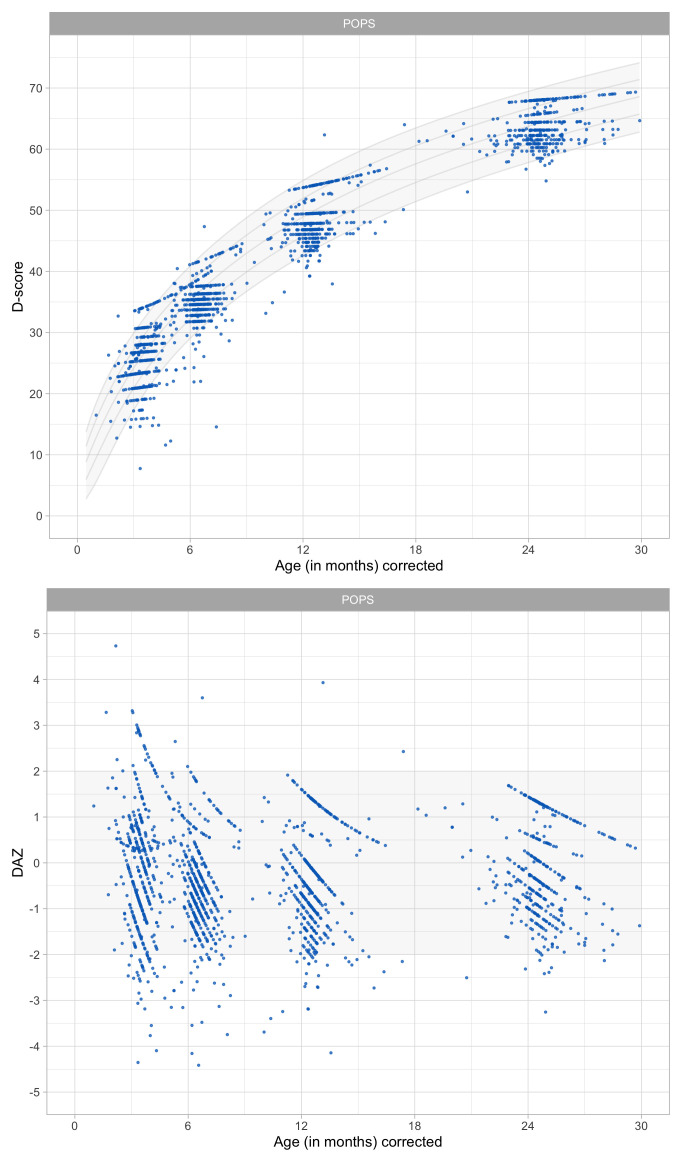
Distribution of D-score and DAZ by child age in a cohort of preterm aged 0–2 years. Ages are corrected for preterm birth by a factor of 0.75 (Source: POPS data, 450 children, four occasions).

Figure 9.2 presents the D-score and DAZ distributions for the POPS cohort of children born very preterm or with very low birth weight. The distributions of the D-score and DAZ are similar to those found in the SMOCC study.

Since the D-scores are calculated using the same milestones and difficulty estimates as used in the SMOCC data, the D-scores are comparable across the two studies. When the milestones differ between studies (e.g. when studies use different measurement instruments), it is still possible to calculate D-scores. This problem is a little more complicated, so we treat it in Chapter II ( van Buuren & Eekhout, 2021).

The primary new complication here is the question whether it is fair to compare *postnatal age* of children born at term with postnatal ages of very preterm children. This section focuses on this issue in some detail.

***9.2.1 POPS design.*** In 1983, the Project On Preterm and Small for Gestational Age Infants (POPS study) collected data on all 1338 infants in the Netherlands who had very preterm birth (gestational age < 32 weeks) or very low birth weight (birth weight < 1500 grams). See Verloove - Vanhorick *et al.* (1986) for details.

The POPS study determined gestational age from the best obstetric estimate, including the last menstrual period, results of pregnancy testing, and ultrasonography findings. The POPS study collected measurements on 450 children using the DDI at four visits at corrected postnatal ages of 3, 6, 12 and 24 months.

***9.2.2 Age-adjustment.*** Assessment of very preterm children at the same chronological age as term children may cause over-diagnosis of developmental delay in very preterm children. Very preterm children may require additional time that allows for development equivalent to that of children born a term.

In anthropometry, it is common to correct chronological age of very preterm born children to enable age-appropriate evaluation of growth. For example, suppose the child is born as a gestational age of 30 weeks, which is ten weeks early. A *full correction* would deduct ten weeks from the child’s postnatal age, and a *half correction* would deduct five weeks. In particular, we calculate the corrected age (in days) as:

correctedage=postnatalage(days)−f×[280−gestationalage(days)],

where 280 is the average gestational age in days, and where we specify several alternatives for *f* as 1.00 (full correction), 0.75, 0.50 (half) or 0.00 (no correction).

Let’s apply the same idea to child development. Using *corrected age* instead of *postnatal age* has two consequences:

•      It will affect the prior distribution for calculating the D-score;

•      It will affect DAZ calculation.

We evaluate these two effects in turn.

**Figure 9.3.**
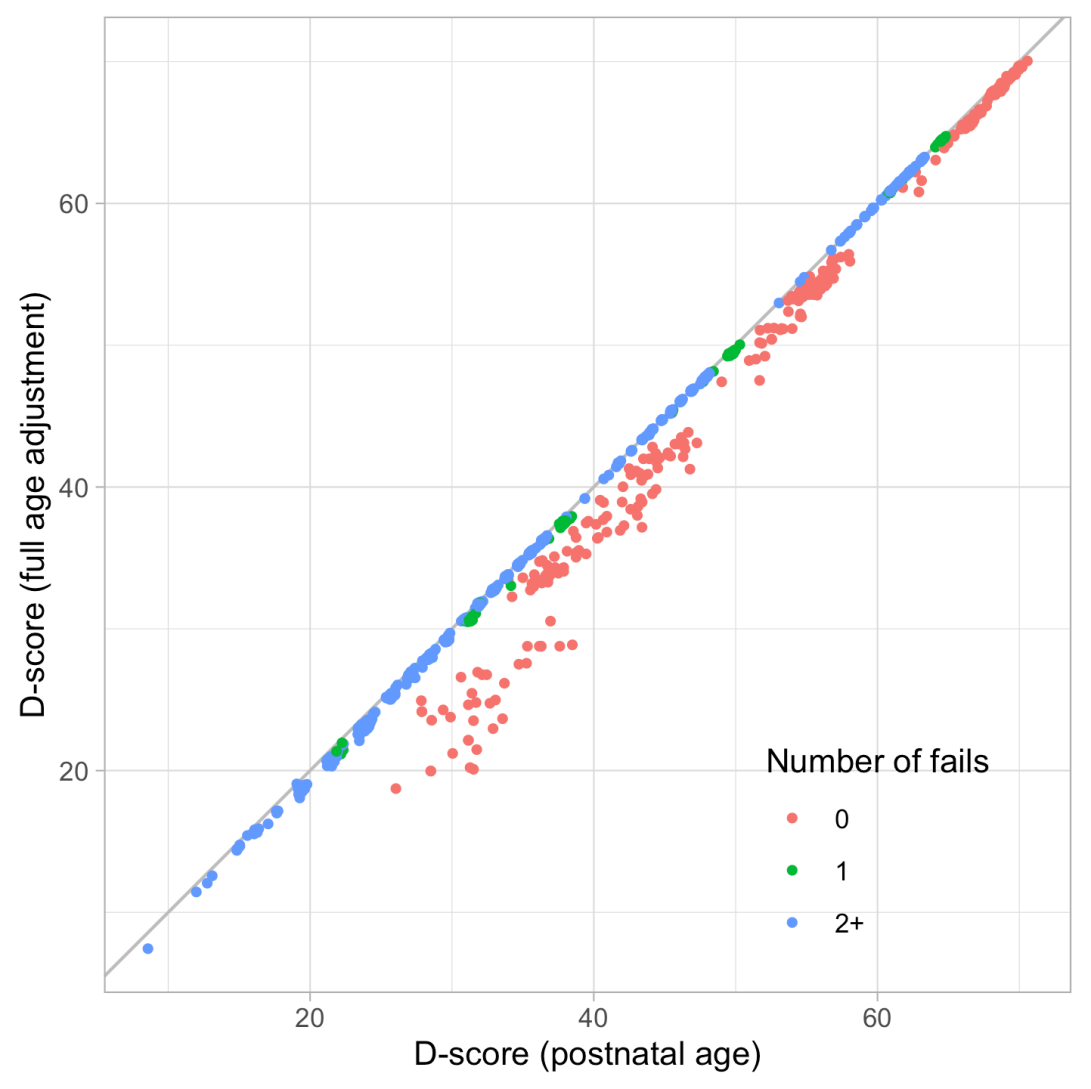
Scatterplot of two versions of the D-score, one calculated using postnatal age ( *f* = 0.00), the other calculated using full age-adjustment ( *f* = 1.00).

***9.2.3 Effect of age-adjustment on the D-score.*** Figure 9.3 plots the fully age-adjusted D-score against the unadjusted D-score. Any discrepancies result only from differences in the ages used in the age-dependent prior (c.f. Section 5.3.2).

All points are on or below the diagonal. Age-adjustment lowers the D-score because a preterm is “made younger” by subtracting the missed pregnancy duration, and hence the prior distribution starts at the lower point. For example, the group of red marks with D-scores between 30 *D* and 40 *D* (age not corrected) will have D-scores between 20 *D* and 30 *D* when fully corrected. Note that only the red points (with perfect scores) are affected, thus illustrating that the prior has its most significant effect on the perfect response pattern. See also Section 5.3.1. The impact of age-correction on the D-score is negligible when the child fails on one or more milestones.

**Figure 9.4.**
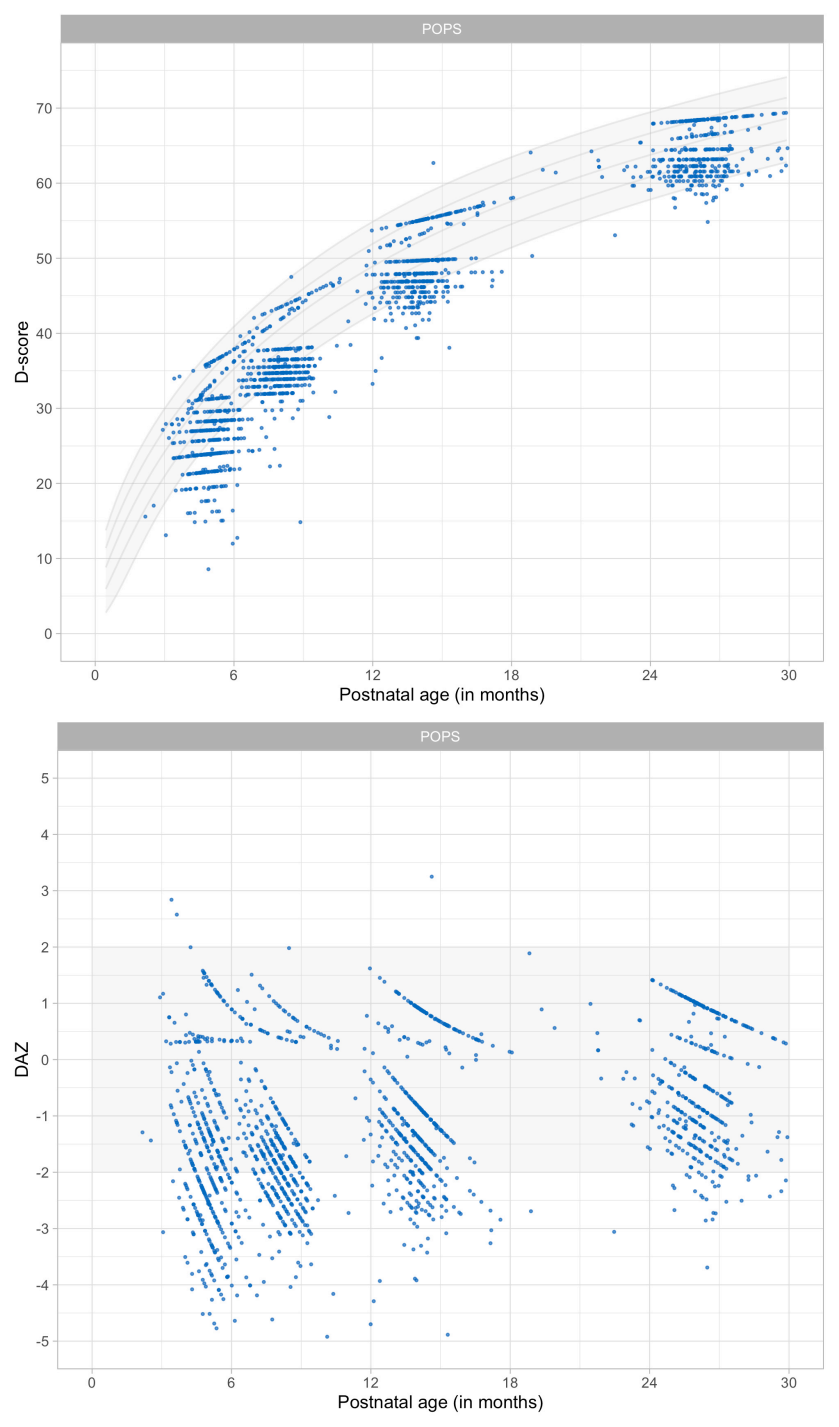
Distribution of D-score and DAZ without age correction for preterm birth ( *f* = 0.00).

***9.2.4 Effect of no age adjustment ( *f* = 0.00) on the DAZ.*** Figure 9.4 illustrates that a considerable number of D-scores fall below the minus -2 SD line of the reference when age is not adjusted, especially during the first year of life. The pattern suggests that the apparent slowness in development is primarily the result of being born early, and does not necessarily reflect delayed development.

**Figure 9.5.**
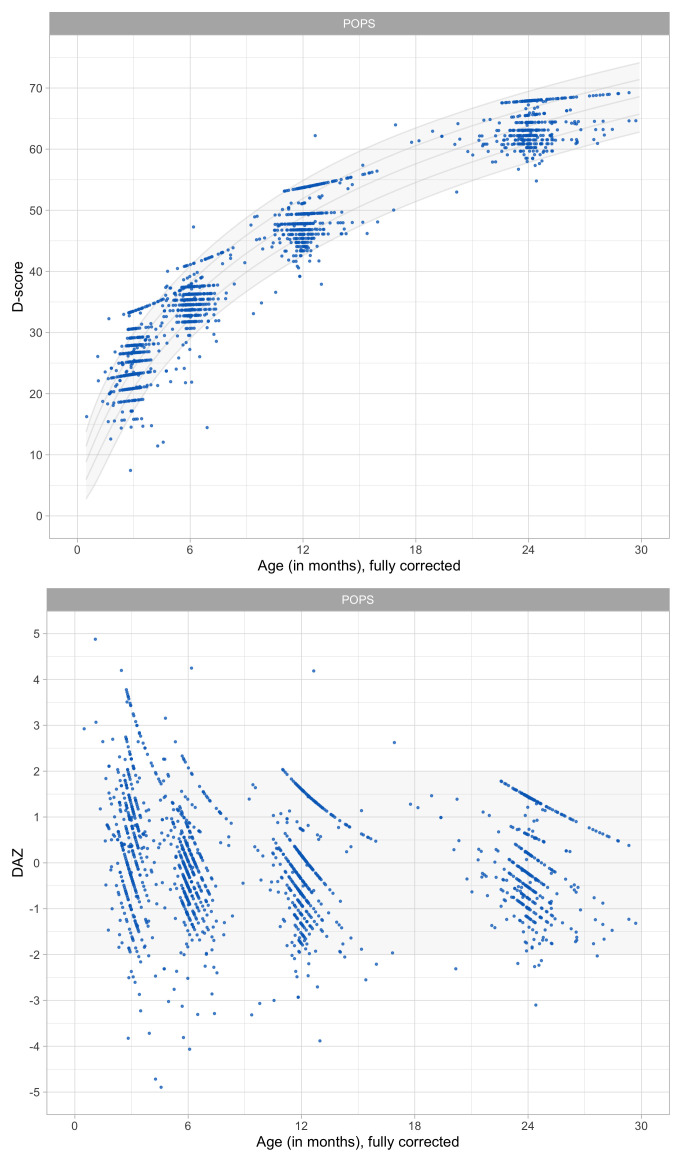
Distribution of D-score and DAZ under full age correction for preterm birth ( *f* = 0.00).

***9.2.5 Effect of full age adjustment ( *f* = 0.00) on the DAZ.*** Full age correction has a notable effect on the DAZ. Figure 9.5 illustrates that the POPS children are now somewhat advanced over the reference children. We ascribe this seemingly odd finding to more prolonged exposure to sound and vision in air. Thus after age correction, development in preterms during early infancy is advanced compared to just-born babies.

Full age correction seems to overcorrect the D-score, so it is natural to try intermediate values for *f* between 0 and 1.

***9.2.6 Partial age adjustment.*** Table 9.1 compares mean DAZ under various specifications for *f*. Values *f* = 0.00 and *f* = 0.50 do not correct for preterm birth enough in the sense that all sign are negative. In contrast, *f* = 1.00 overcorrects. The value of 0.73 is implausibly high, especially because this value is close to birth. Setting *f* = 0.75 seems a good compromise, in the sense that the average DAZ is close to zero in the first age interval. The average DAZ is negative at later ages. We do not know whether this genuinely reflects less than optimal development of very preterm and low birth weight children, so either *f* = 1.00 and *f* = 0.75 are suitable candidates.

**Table 9.1.** Average DAZ at various ages under four correction factors.

Age (months)	0.00	0.50	0.75	1.00
0–3	-1.46	-0.50	0.07	0.73
3–4	-1.77	-0.89	-0.37	0.20
5–6	-1.60	-0.87	-0.46	0.00
7–8	-1.76	-1.13	-0.77	-0.39
9-–1	-1.21	-0.77	-0.53	-0.28
12–14	-0.99	-0.60	-0.39	-0.16
15–23	-0.50	-0.23	-0.10	0.04
24+	-0.70	-0.49	-0.37	-0.24

***9.2.7 Conclusions.***

•      Compared with the general population, more very preterm children reached developmental milestones within chronological age five months when chronological age was fully corrected;

•      Fewer preterm children reached the milestones when chronological age was not corrected;

•      Fewer children reached the milestones when we used a correction of *f* = 0.50;

•      Similar proportions were observed when we used *f* = 0.75 within the first five months after birth.

•      After chronological age five months, we observed similar proportions for very preterm and full-term children when chronological age was fully corrected.

•      We recommend using full age correction ( *f* = 1.00). This advice corresponds to current practice for growth and development. As we have shown, preterms may look better in the first few months under full age-correction. If the focus of the scientific study is on the first few months, we recommend an age correction of *f* = 0.75.

### 9.3 TOGO study

**Figure 9.6.**
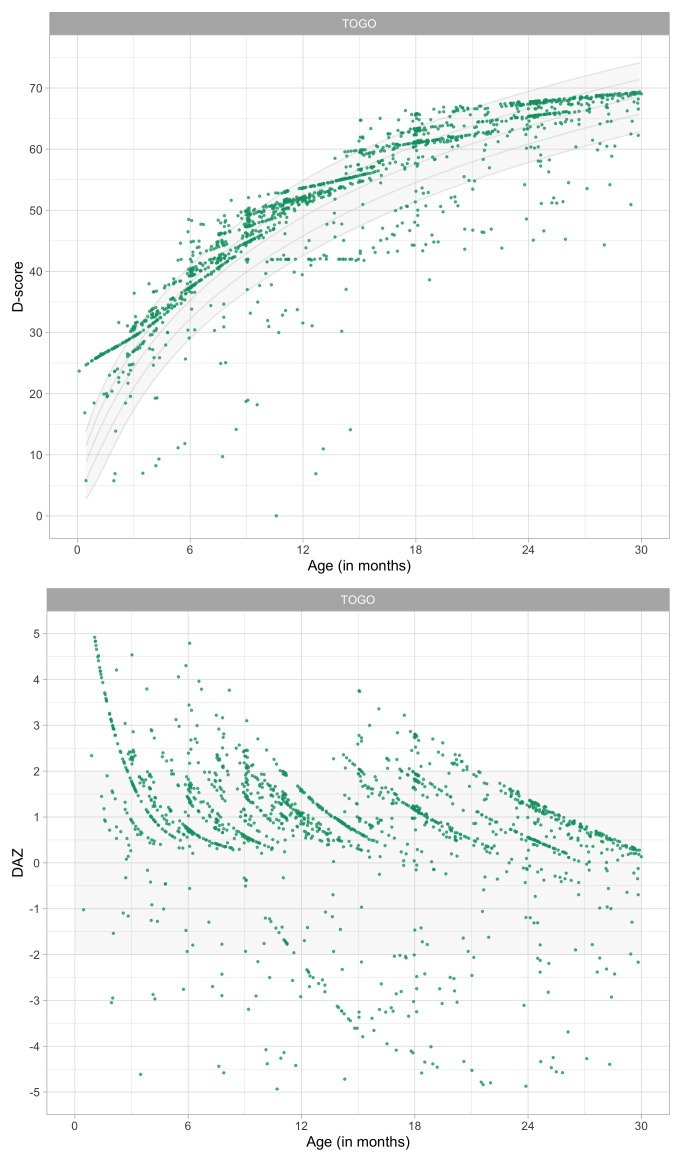
Distribution of D-score and DAZ by child age of children living near Kpalimé, Togo (Source: TOGO data, *n* = 1567).

Figure 9.6 presents the D-score and DAZ distributions of a sample of children living near Kpalimé, Togo. While the primary trend with age conforms to the previous data, the distributions differ from those in Figure 9.1 and Figure 9.2 in two respects:

• *Compression at the upper end*: Most of the D-scores are above the median curve, which suggests that, at these ages, children living in Togo *develop faster* than children living in the Netherlands;

• *Expansion at the lower end*: There is a considerable variation in D-scores on the lower end, with many D-scores below the -2 SD curve, suggesting that some children are *significantly more delayed* than would be expected in both Dutch samples.

The D-scores are calculated using the same 57 milestones and difficulty estimates as before. The resulting D-score distribution is quite unusual. The main question here is what could explain the pattern found in the D-scores. This section explores this question in some detail.

***9.3.1 Togo Kpalimé study, design.*** If the D-score is to be a universal measure, then it should be informative in *low and middle-income countries* (LMIC) as well. We do not yet know much about the usability and validity of the D-score in LMIC’s. The western African country of Togo qualifies as a low-income country, with a 2017 GNI per capita of USD 610, compared to USD 46,180 in the Netherlands, and USD 744 for low-income countries in general (data.worldbank.org).

The data were collected by Cécile Schat-Savy, who initiated a youth health care centre modelled after the Dutch youth health care system in Kpalimé, Togo. See https://www.kinderhulp-togo.nl for more background. Data monitoring included a french translation the DDI for measuring child development. The investigators gathered data from 9747 individuals in the 0–18 age range.

Participants include children and their parents who visited the Kpalimé health centre at least one time. Kpalimé is the fourth largest town in Togo, but the health centre also attracted parents and children from a wide surrounding rural area. Parents visited the health centre for several reasons, including for a preventive health check or because of their child’s apparent health problems.

**Figure 9.7.**
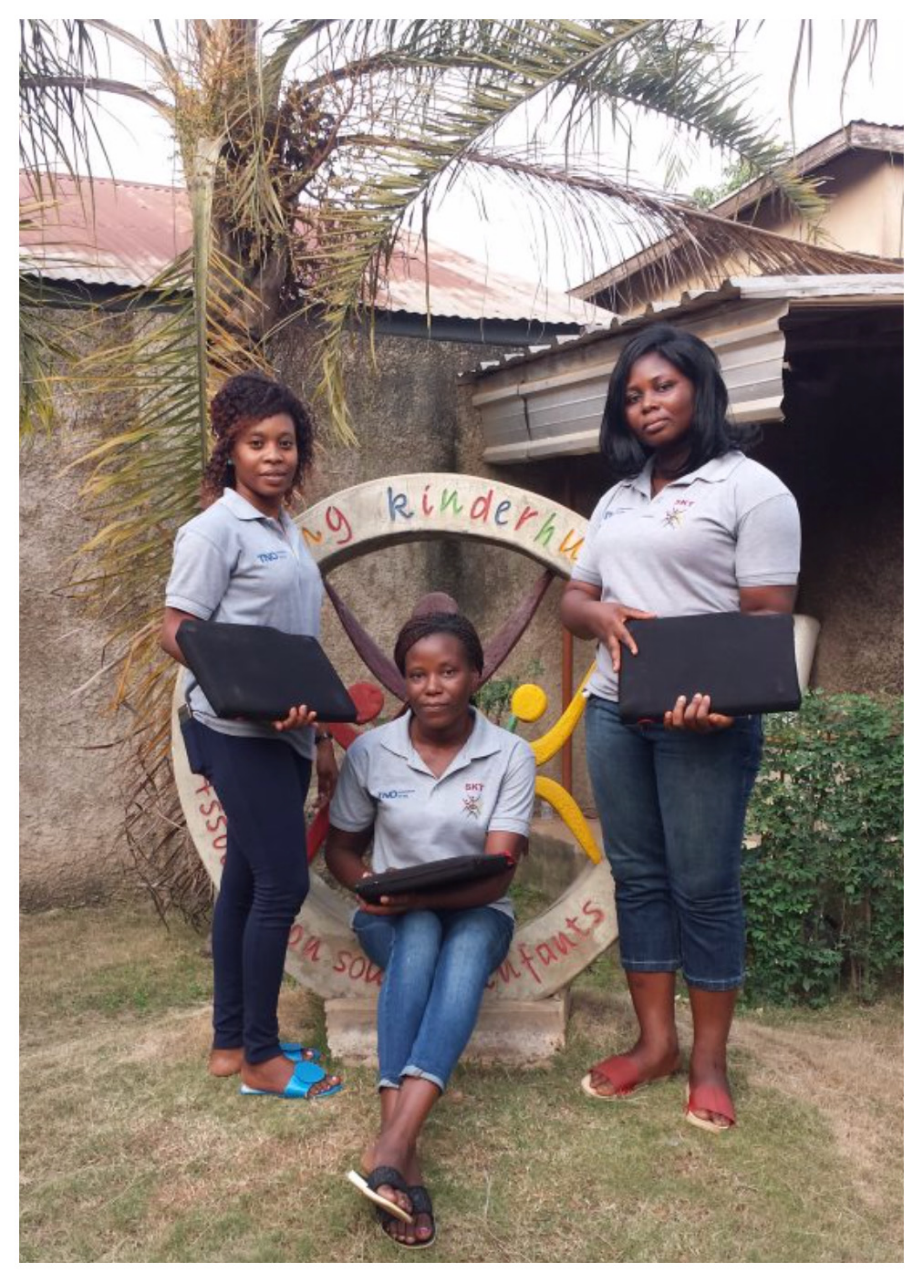
Three of the data-assistants who helped to digitize the paper files. Reproduced with permission from Stichting Kinderhulp Togo https://www.kinderhulp-togo.nl.

The health centre targeted parents through information sessions for parents at primary schools. Parents paid a small amount of money per child (about USD 4.00 for children of 4 years or older, and USD 0.80 for children younger than four years). Four local data-assistants, some portrayed in Figure 9.7, digitized the data from paper archives. TNO Child Health in The Netherlands monitored the process and checked the data for completeness and consistency.

Here we use a subset of 2674 visits from 1644 unique children who scored on the 57 milestones of the DDI 0–2 years. We did not calculate D-scores when age or DDI milestones were missing, which left a dataset of 2425 visits from unique 1567 children. The number of visits varied from 1 – 9. The majority of children visited the centre once.

**Figure 9.8.**
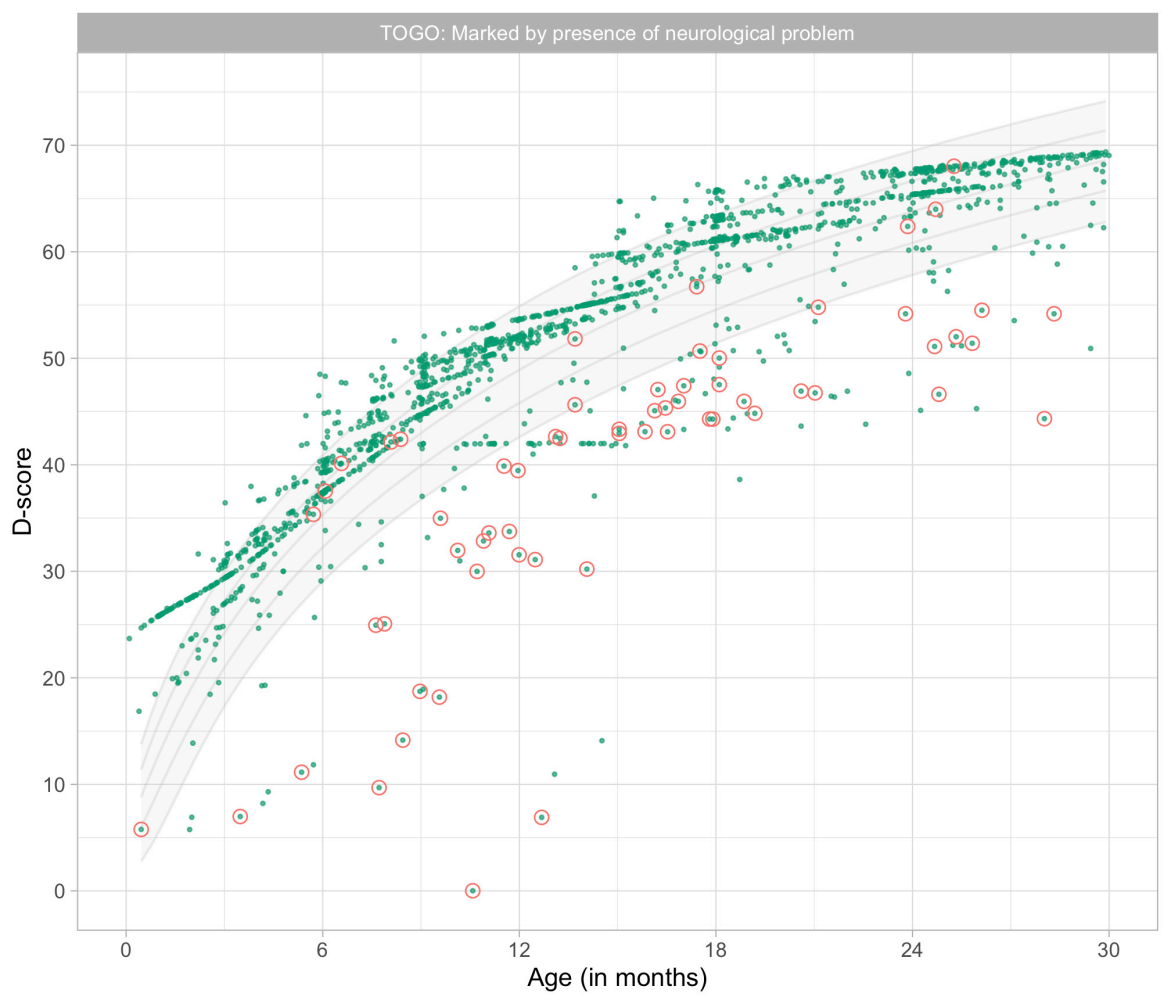
Distribution of D-score by age labelled by neurological (tonus and/or reflex) problems. (Source: TOGO data).

***9.3.2 D-score labelled by neurological problem.*** Figure 9.8 is the same scatter plot as in Figure 9.6, but now marked by whether the physician registered signs of neuropathology in the form of tonus and reflex problems.

Many children with low D-scores also have tonus or reflex problems. This finding alone suggests that extreme D-score are not artefacts (e.g. caused by a wrongly coded age), but indicate main adverse health conditions.

**Figure 9.9.**
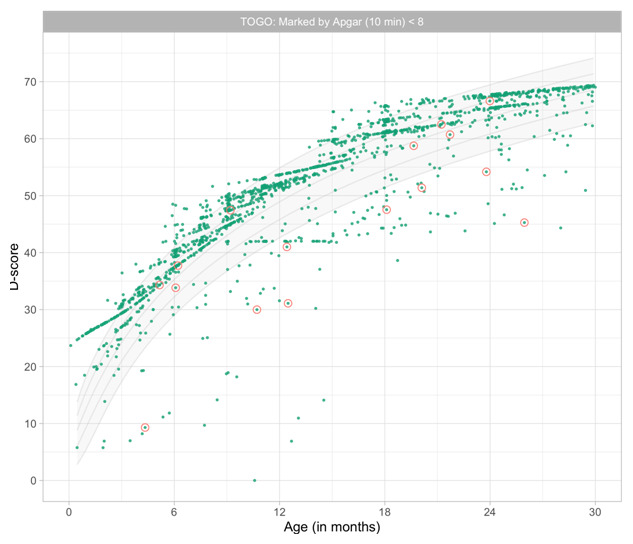
Distribution of D-score by age labelled by Apgar score (10 minutes) lower than 8. (Source: TOGO data).

***9.3.3 D-score labelled by Apgar score*** Figure 9.9 identifies the children who had an Apgar score at 10 minutes after birth that was lower than 8. About half of these children had a D-score below -2 SD curve.

**Figure 9.10.**
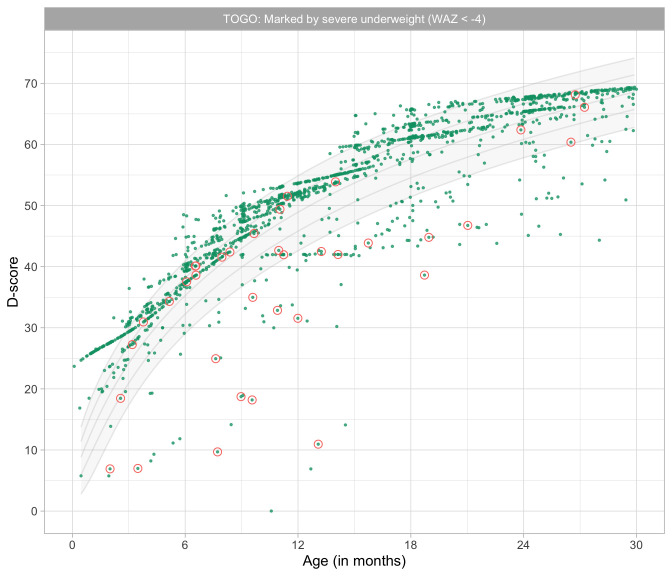
Distribution of D-score by age labelled by severe underweight (WAZ < -4) (Source: TOGO data).

***9.3.4 D-score labelled by severe underweight.*** Many children who visited the Kpalimé health centre had a low body weight for their age. Figure 9.10 marks the subset of severely underweight children (WAZ < -4). A substantial proportion of these children also had a very low D-score.

**Figure 9.11.**
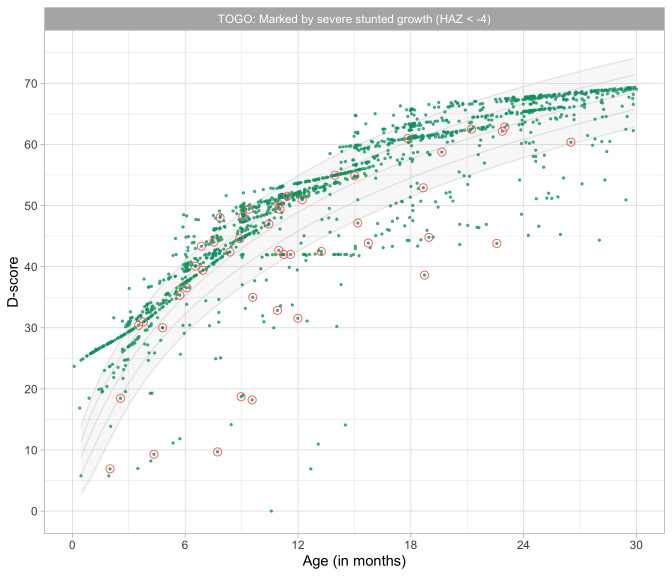
Distribution of D-score by age labelled by severe stunting (HAZ < -4) (Source: TOGO data).

***9.3.5 D-score labelled by severe stunting*.** Figure 9.11 is similar to 9.10, but now marked by the subset of severely stunted children (HAZ < -4). Also here, a sizable proportion has a low D-score.

When taken together, Figure 9.8– Figure 9.11 show that children with very low D-scores often experience (multiple) harsh health problems. Those health problems may have substantially delayed their development.

**Figure 9.12.**
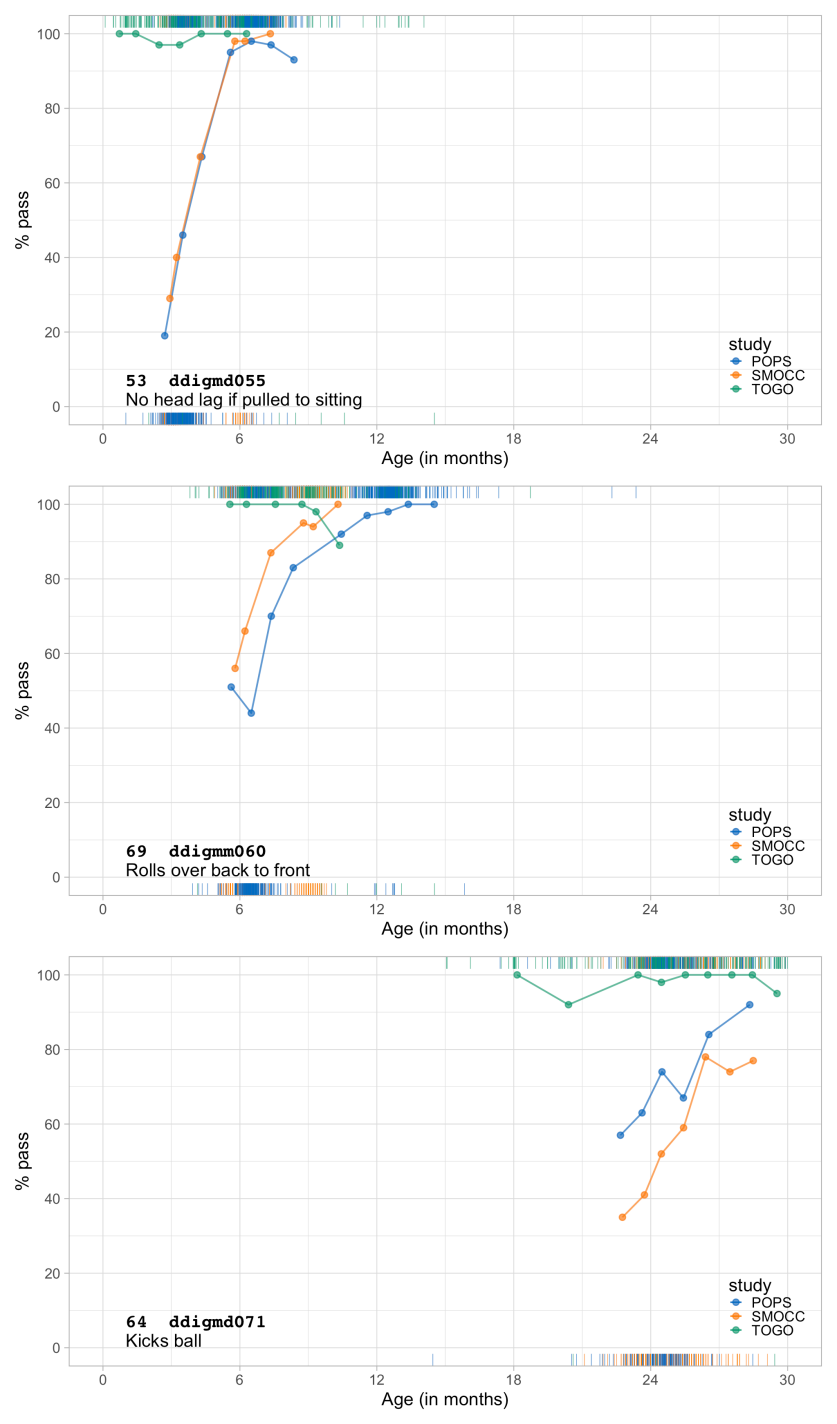
Gross motor milestones. Probability by age for SMOCC, POPS (corrected age) and TOGO studies for three milestones.

***9.3.6 Gross motor development.*** Figure 9.12 shows substantial differences in gross motor development between children from Togo and the Netherlands. For example, at the age of three months, about 30 per cent of the Dutch infants succeed in controlling their head when pulled to sitting. However, infants from Togo seem already capable of head control when they are just one month old.

Moreover, the advantage persists at least until up to the age of two years: children in Togo can roll over and sit much earlier, or kick a ball without falling. As the documentary Babies shows, African children even manage to learn to walk with a tin can on their head, a craft that children in the west never achieve.

**Figure 9.13.**
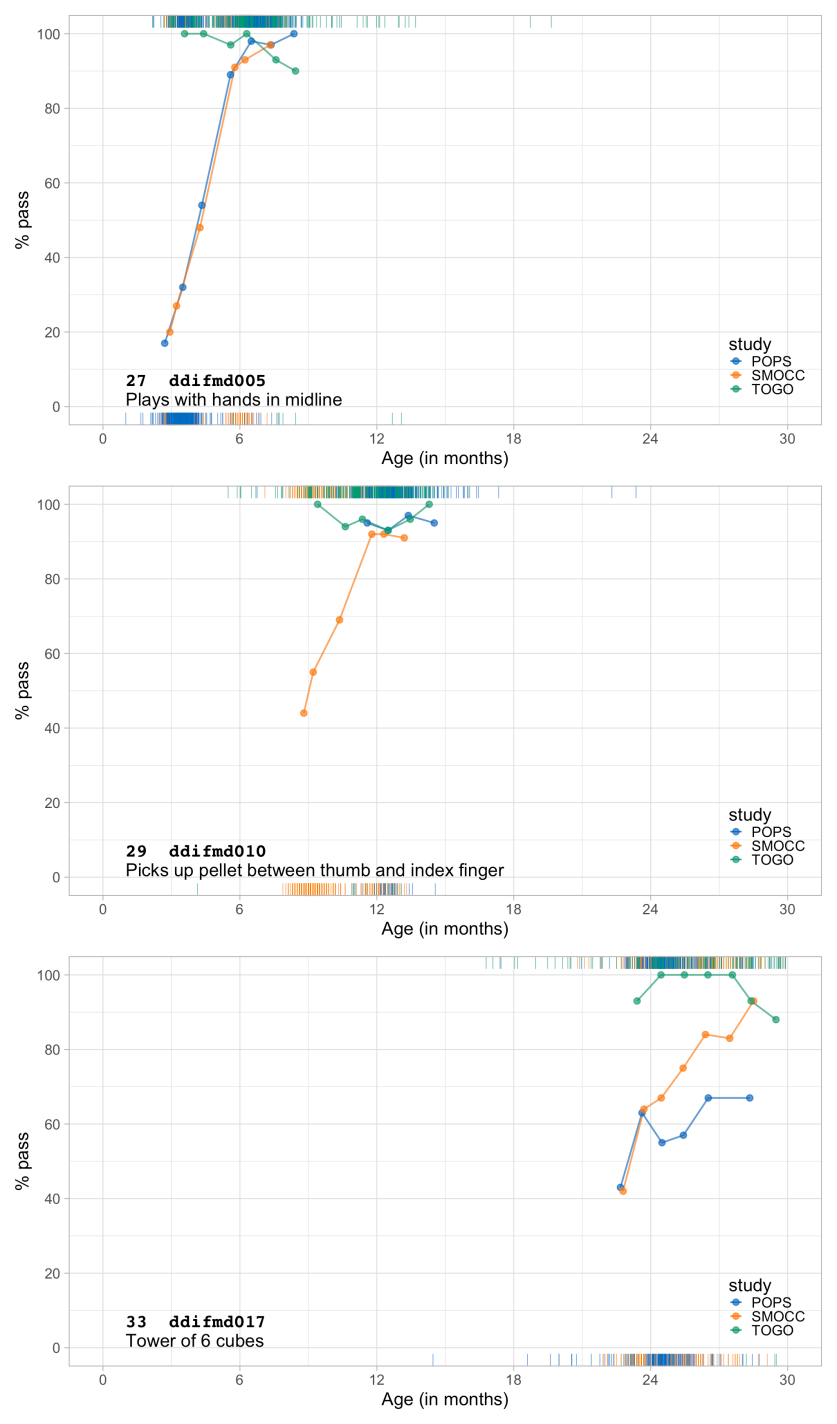
Fine motor milestones. Probability by age for SMOCC, POPS (corrected age) and TOGO studies for three milestones.

***9.3.7 Fine motor development.*** Figure 9.13 shows a less pronounced but similar phenomenon for fine motor skills. These data suggest that children in Togo may have better fine motor skills than the children from the two Dutch cohorts.

**Figure 9.14.**
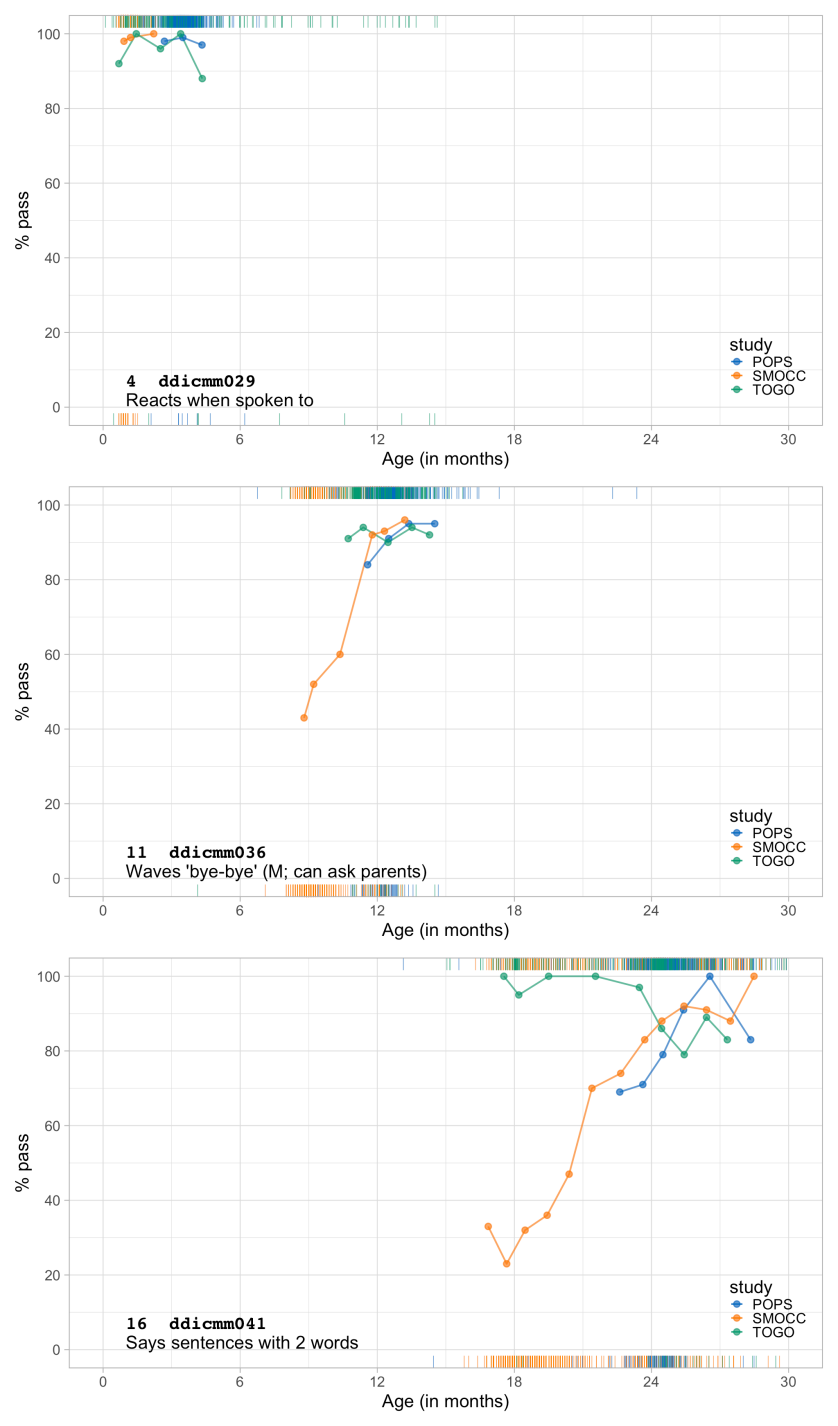
Communication and language milestones. Probability by age for SMOCC, POPS (corrected age) and TOGO studies for three milestones.

***9.3.8 Communication and language.*** Figure 9.14 summarizes the data for three milestones on communication and language. In general, the success probability is similar in the three studies.

One curious finding is that the high proportion of milestones passes in ddicmm041 for the Togo children around the age of 18 months. Note that several of the green lines in Figure 9.12– Figure 9.14 start close to perfect scores, which makes it impossible to show the rising patterns found in the Dutch data.

It may indeed be true that children from Togo develop more rapidly than Dutch children. But we may also wonder: Could there just be reporting bias on the part of the parents who either do not understand the items or have the expectation to say “yes” even if the child can’t do it? It would be desirable if these results could be backed up from other sources.

### 9.4 Conclusions

This section compared the D-scores estimated from the DDI administered to three different groups of children.

We found that

•      The D-score by age plot showed a positive, curved relationship with age in all three studies;

•      Children born very preterm or with very low birth weight had similar development to reference children when their age was corrected for early birth;

•      A relatively small subset of children born in Togo had extremely low D-scores, not found in the Netherlands, likely the result of underlying neuropathology, severe underweight or severe stunting;

•      On average, children from Togo seemed to have faster development during the first two years, especially in motor development, though there may be issues with reporting bias.

All in all, these findings support the usefulness and validity of the D-score as an informative summary of child development during their first two years of life.

## 10 Next steps

This section provides a quick overview of the relevance, concepts and techniques of the D-score. While the results obtained thus far are encouraging, some questions will certainly remain when we put the method to practice.

A rough selection of such questions includes:

•      What is the added value of the D-score in practice?

•      Does the D-score extend to higher ages?

•      Is the assumption of uni-dimensionality reasonable for other ages and populations?

•      Can we calculate the D-score from instruments other than the DDI?

•      Is it reasonable to assume that milestone difficulty is identical in other populations?

•      Does the method apply to caregiver-reported milestones?

•      Would a dedicated D-score instrument be more efficient?

•      How many milestones are “enough?”

•      Can the same scale be used for measurement at individual, group and population levels?

•      Can the D-score detect delayed development?

•      Would the D-score help to target early interventions?

This section briefly reviews some of these issues.

### 10.1 Usefulness of D-score for monitoring child health

The D-score is a new approach to measure child development. The D-score is a scale for quantifying generic child development by a single number. Milestones are selected to fit the Rasch model. We can interpret the resulting measurements as scores on an interval scale, a requirement for answering questions like:

•      What is the difference in development over time for the same child, group or population?

•      What is the difference in development between different children, groups or populations of the same age?

•      How does child development compare to a norm?

The concept of the D-score is broader than a score calculated from the DDI. Any instrument that fits the model underlying the D-score can be used to measure the child’s D-score.

The precision of the measurement depends on the number of milestones and the match between milestone difficulty and person ability. We may thus tailor the measurement instrument to the question at hand.

### 10.2 D-chart, a growth chart for child development

The field of child growth and development roughly divides into two areas:

•      The subfield *child growth* (or *auxology*) emphasizes body measures like height, weight, body mass index, and so on. It is a rigorous quantitative science with intimate ties to statistics since the days of Quetelet and Galton.

•      The subfield *child development* is more recent and builds upon a wide-ranging set of domain-specific instruments for measuring motor, language, cognitive and behavioural states.

The *growth chart* is a widely used tool to monitor physical growth. The D-score can be used in a similar way to create the *D-chart*.

**Figure 10.1.**
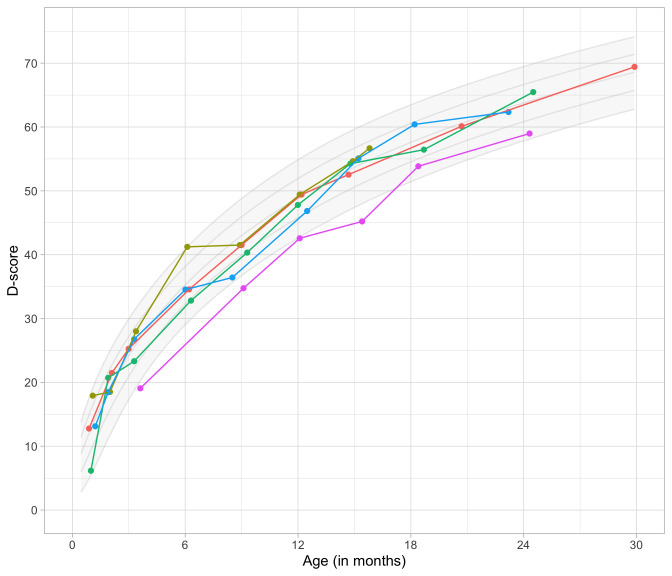
D-chart with five children from the SMOCC study.

Figure 10.1 shows the developmental paths of five randomly chosen children from the SMOCC study. Although the milestones differ across age, there is only one vertical axis. These trajectories will help to track the progress of a child over time.

The D-chart shows that it is straightforward to apply quantitative techniques from child growth to child development. Our hope is that D-score aids in bridging the disparate subfields of child growth and child development.

### 10.3 Opportunities for early intervention

Black *et al.* (2017) estimated that about 250 million children worldwide fail to reach their developmental potential. Developmental delays become evident in the first year and worsen during early childhood. The burden of children not reaching their developmental potential is high.

Interventions aimed at improving child development work best when delivered at the sensitive periods. Programs are to be comprehensive, incorporating a combination of health, nutrition, security and safety, responsive caregiving and early learning. See Engle *et al.* (2011); Grantham‐McGregor *et al.* (2014) and Britto *et al.* (2017) for recent overviews and initiatives.

The lack of a universal measure for child development has long hampered the ability to estimate intervention effects or to compare populations. The D-score can be generalized to other instruments. We expect that the availability of a common yardstick will stimulate informed policy and priority setting. We hope a universal measure improves decision making, ultimately lowering the number of children not reaching their developmental potential.

### 10.4 D-score for international settings

Section 9 compared D-scores between three study samples. We restricted the analysis to studies that used the same instrument (the DDI, in Togo, translated to French) to measure child development.

It is difficult to compare levels of child development worldwide. Existing estimates on children not reaching their developmental potential rely on proxies, such as stunting and poverty. While these proxies have been found to correlate with child development, they are only weak indicators of actual child performance. Arguably, the performance of a child on a set of well-chosen milestones is more informative for his or her future health and productivity than body height or parental income.

There are more than 150 instruments are available that quantify child development. Many of these tools produce not just one but many scores. Such an overwhelming choice may seem a luxury until we realize that we cannot compare their ratings. Of course, we could settle on just one instrument …., but that’s never going to happen. While simple in theory, pre-harmonization is complicated in practice. It requires significant and continued investments by a central agency. It does not address historical data, so it will be challenging to see secular trends. Also, pre-harmonization impedes the adoption of innovative techniques, e.g., using smartphone-assisted evaluations.

The D-score opens up an exciting alternative: *agree on the scale*, and leave some liberty to the data-collector in the exact choice of the instrument. We could build upon the expertise of the data collector about the local population. Also, it will equip is to keep up with innovations in measurement.

The next chapter in our work will address some of the conceptual and technical issues that arise when we attempt to apply the D-score to other populations.

### 10.5 D-score from existing instruments

There is a vast base of historic child developmental data using existing instruments. The problem is that each device defines its own summaries, so we cannot compare scores across tools. Different instruments have different domains, various age forms, different stopping rules, diverse age norms, and so on. Yet, the milestones in these instruments are often very similar. Most tools collect data on milestones like:

•      Can the child stack two blocks?

•      Can the child roll over?

•      Can the child draw a cross?

•      Can the child stand?

•      Can the child say “baba?”

With the D-score methodology in hand, we are ready to exploit the overlap in milestones shared by different instruments. Common items can act as *bridges*, so - with the appropriate item-level data - we may attempt calculating D-scores from other tools as well.

The task is to identify milestones that overlap between both instruments, filter out milestones that do not fit a joint model, and estimate the item difficulties of items that remain. Chapter II ( van Buuren & Eekhout, 2021) will explore this possibility in more detail.

### 10.6 Creating new instruments for D-score

Extending the D-score to other instruments has the side-effect of enlarging the item bank with useful items. As more and more data feed into the item bank, assessment of already present milestones may become more precise.

The enlarged and improved item bank then may act as the fundamental resource for creating instruments for particular settings. For example, if the interest is on finding the most advanced children, we may construct a difficult test that will separate the good and the best. Alternatively, we can use the item bank to create and administer *computerized adaptive tests* ( Jacobusse & van Buuren, 2007; Wainer *et al.*, 2000), a sequential method that selects the next milestone based on the previous test outcome.

Our ongoing work will explore the conceptual and technical challenges, and propose an integrated approach to support instrument construction and validation.

## Appendices

### A - Notation

The notation in this chapter follows Wright & Masters (1982).

**Table:** 

Section	Symbol	Term	Description
4.6	*β*_*n*_	Ability	True (but unknown) developmental score of child *n*
4.6	*δ*_*i*_	Difficulty	True (but unknown) difficulty of item *i*
4.6	*π*_*ni*_	Probability	True (but unknown) probability that child *n* passes item *i*
6.1	${\hat{\beta }}_{n}$	Ability	Estimated developmental score (D-score) of child *n*
6.1	${\hat{\delta }}_{i}$	Difficulty	Estimated difficulty of item *i*
6.1	*P*_*ni*_	Probability	Estimated probability that child *n* passes item *i*
6.1	*x*_*ni*_	Data	Observed response of child *n* on item *i*, 0 or 1
6.1	*W*_*ni*_	Variance	Variance of *x*_*ni*_
6.1	*z*_*ni*_	Residual	Standardized residual between *x*_*ni*_ and *P*_*ni*_
6.1	*N*	Count	Number of measurements (children)
6.1	*L*	Count	Number of items (milestones)
6.4	*P*( ${\hat{\delta }}_{i}$ )	Probability	Conditional probability of passing item *i*
6.4	*I*( ${\hat{\delta }}_{i}$ )	Information	Item information function of item *i*
6.5	*R*	Reliability	True test reliability
6.5	$\hat{R}$	Reliability	Estimated test reliability
6.5	$\sigma_{e}^{2}$	Variance	True error variance
6.5	${\hat{\sigma }}_{\hat{e}}^{2}$	Variance	Estimated error variance
6.5	${\hat{\sigma }}_{\hat{e}}$	Variance	Standard error of measurement (sem)
9.2	*f*	Factor	Age-adjustment factor

### B - Technical information


R version 4.0.4 (2021-02-15)
Platform: x86_64-apple-darwin17.0 (64-bit)
Running under: macOS Big Sur 10.16

Matrix products: default
BLAS:   /Library/Frameworks/R.framework/Versions/4.0/Resources/lib/libRblas.dylib
LAPACK: /Library/Frameworks/R.framework/Versions/4.0/Resources/lib/libRlapack.dylib

locale:
[1] en_US.UTF-8/en_US.UTF-8/en_US.UTF-8/C/en_US.UTF-8/en_US.UTF-8

attached base packages:
[1] stats     graphics  grDevices utils     datasets  methods   base     

other attached packages:
 [1] dinstrument_0.0.1.2 ddata_0.52.0        gseddata_1.5.1     
 [4] dmetric_0.52.0      dscore_1.4.0.9000   forcats_0.5.1      
 [7] haven_2.3.1         scales_1.1.1        plotly_4.9.3       
[10] sirt_3.9-4          gridExtra_2.3       plyr_1.8.6         
[13] reshape2_1.4.4      RColorBrewer_1.1-2  dplyr_1.0.4        
[16] tidyr_1.1.2         ggplot2_3.3.3       officer_0.3.17.001 
[19] officedown_0.2.1    kableExtra_1.3.2    knitr_1.31         

loaded via a namespace (and not attached):
 [1] nlme_3.1-152      webshot_0.5.2     httr_1.4.2        tools_4.0.4      
 [5] R6_2.5.0          DBI_1.1.1         lazyeval_0.2.2    colorspace_2.0-0 
 [9] withr_2.4.1       tidyselect_1.1.0  compiler_4.0.4    polycor_0.7-10   
[13] rvest_0.3.6       TAM_3.5-19        xml2_1.3.2        bookdown_0.21    
[17] mvtnorm_1.1-1     gamlss_5.2-0      systemfonts_1.0.1 stringr_1.4.0    
[21] digest_0.6.27     rmarkdown_2.7     pkgconfig_2.0.3   htmltools_0.5.1.1
[25] fastmap_1.1.0     rvg_0.2.5         htmlwidgets_1.5.3 rlang_0.4.10     
[29] rstudioapi_0.13   shiny_1.6.0       generics_0.1.0    gamlss.data_5.1-4
[33] jsonlite_1.7.2    gtools_3.8.2      zip_2.1.1         magrittr_2.0.1   
[37] Matrix_1.3-2      Rcpp_1.0.6        munsell_0.5.0     gdtools_0.2.3    
[41] lifecycle_1.0.0   stringi_1.5.3     yaml_2.2.1        MASS_7.3-53.1    
[45] gamlss.dist_5.1-7 grid_4.0.4        parallel_4.0.4    promises_1.2.0.1 
[49] crayon_1.4.1      lattice_0.20-41   splines_4.0.4     hms_1.0.0        
[53] pillar_1.4.7      uuid_0.1-4        glue_1.4.2        evaluate_0.14    
[57] data.table_1.13.6 vctrs_0.3.6       httpuv_1.5.5      gtable_0.3.0     
[61] purrr_0.3.4       assertthat_0.2.1  cachem_1.0.4      CDM_7.5-15       
[65] xfun_0.21         mime_0.10         xtable_1.8-4      later_1.1.0.1    
[69] survival_3.2-7    viridisLite_0.3.0 tibble_3.0.6      memoise_2.0.0    
[73] ellipsis_0.3.1 




## Data availability

### Underlying data

The raw data needed to replicate these analyses are not public, so we cannot share it with this publication. However, the reader can apply for access to the data through the study contact. The table given below contains the contact information for each cohort included in this publication.

A subset of studies made their study data publicly available under a CC BY 4.0 license ( https://creativecommons.org/licenses/by/4.0/) ^1^. Authorship remains with the study coordinator, but users are free to redistribute, alter and combine the data, on the condition of giving appropriate credit with any redistributions of the material. The URL of the public data is https://d-score.org/childdevdata/.

**Table:** 

Name in publication	Reference	Contact
GCDG-BGD-7MO	Hamadani *et al.*, 2011; Tofail, 2009	Jena Hamadani ( jena@icddrb.org)
GCDG-BRA-1	Halpern *et al.*, 1996; Victora *et al.*, 2006	Simone Karam ( Karam.simone@gmail.com)
GCDG-BRA-2	Moura *et al.*, 2010	Simone Karam ( Karam.simone@gmail.com)
GCDG-CHL-1	Lozoff *et al.*, 2013	Betsy Lozoff ( blozoff@umich.edu)
GCDG-CHL-2	Conteral & González, 2015	Lia Fernald ( fernald@berkeley.edu)
GCDG-CHN	Angulo-Barroso *et al.*, 2016; Lozoff *et al.*, 2016; Santos *et al.*, 2017	Betsy Lozoff ( blozoff@umich.edu)
GCDG-COL-LT45M	Andrew *et al.*, 2017; Attanasio *et al.*, 2014	Marta Rubio ( martarubio@iadb.org)
GCDG-COL-LT42M	Rubio-Codina *et al.*, 2016	Marta Rubio ( martarubio@iadb.org)
GCDG-ECU	Araujo *et al.*, 2016; Fernald & Hidrobo, 2011; Paxon & Shady, 2010	Caridad Araujo ( mcaraujo@iadb.org)
GCDG-ETH	Hanlon *et al.*, 2016	Charlotte Hanlon ( charlotte.hanlon@kcl.ac.uk)
GCDG-JAM-LBW	Walker *et al.*, 2004; Walker *et al.*, 2010	Susan Walker ( susan.walker@uwimona.edu.jm)
GCDG-JAM- STUNTED	Grantham-McGregor *et al.*, 1991; Grantham-McGregor *et al.*, 1997; Walker *et al.*, 2005	Susan Walker ( susan.walker@uwimona.edu.jm)
GCDG-MDG	Galasso *et al.*, 2011; Galasso *et al.*, 2017	Ann Weber ( annweber@stanford.edu)
GCDG-NLD-SMOCC	Herngreen *et al.*, 1992	Paul Verkerk ( paul.verkerk@tno.nl)
GCDG-NLD-2	Doove *et al.*, 2010; Doove *et al.*, 2019;	Bernice Doove ( bernice.doove@maastrichtuniversity.nl)
GCDG-ZAF	Richter *et al.*, 1995; Richter *et al.*, 2004; Richter *et al.*, 2007; Yach *et al.*, 1991	Linda Richter ( Linda.Richter@wits.ac.za)
TOGO	Van Buuren & Eekhout, 2021	Cécile Schat-Savy ( cschatsavy@kinderhulp-togo.nl)
POPS	Verloove - Vanhorick *et al.*, 1986	Sylvia van de Pal ( sylvia.vanderpal@tno.nl)

## References

[ref-1] Andrich D : A Rating Formulation for Ordered Response Categories. *Psychometrika.* 1978;43(4):561–73. 10.1007/BF02293814

[ref-2] Baarda DB : UKKI: Utrechtse Korte Kleuter Intelligentietest: Handleiding.Lisse: Swets en Zeitlinger,1978. Reference Source

[ref-3] Baird G Simonoff E Pickles A : Prevalence of Disorders of the Autism Spectrum in a Population Cohort of Children in South Thames: The Special Needs and Autism Project (SNAP). *Lancet.* 2006;368(9531):210–15. 10.1016/S0140-6736(06)69041-7 16844490

[ref-4] Bellman M Byrne O Sege R : Developmental Assessment of Children. *BMJ.* 2013;346(e8687):e8687. 10.1136/bmj.e8687 23321410

[ref-5] Berk LE : Child Development. 9th Ed.Boston, MA: Pearson.2011.

[ref-6] Berkson J : Application of the Logistic Function to Bio-Assay. *J Am Stat Assoc.* 1944;39(227):357–65. 10.1080/01621459.1944.10500699

[ref-7] Black MM Walker SP Fernald LCH : Early Childhood Development Coming of Age: Science Through the Life Course. *Lancet.* 2017;389(10064):77–90. 10.1016/S0140-6736(16)31389-7 27717614 PMC5884058

[ref-9] Bock RD Mislevy RJ : Adaptive EAP Estimation of Ability in a Microcomputer Environment. *Appl Psychol Meas.* 1982;6(4):431–44. 10.1177/014662168200600405

[ref-10] Boggs D Milner KM Chandna J : Rating Early Child Development Outcome Measurement Tools for Routine Health Programme Use. *Arch Dis Child.* 2019;104(Suppl 1):S22–33. 10.1136/archdischild-2018-315431 30885963 PMC6557219

[ref-11] Britto PR Lye SJ Proulx K : Nurturing Care: Promoting Early Childhood Development. *Lancet.* 2017;389(10064):91–102. 10.1016/S0140-6736(16)31390-3 27717615

[ref-12] Cameron N Bogin B : Human Growth and Development.London: Academic Press,2012. Reference Source

[ref-13] Caspi A Hariri AR Holmes A : Genetic Sensitivity to the Environment: The Case of the Serotonin Transporter Gene and Its Implications for Studying Complex Diseases and Traits. *Am J Psychiatry.* 2010;167(5):509–27. 10.1176/appi.ajp.2010.09101452 20231323 PMC2943341

[ref-14] Clark H Coll-Seck AM Banerjee A : A Future for the World’s Children? A WHO–UNICEF– *Lancet* Commission. *Lancet.* 2020;395(10224):605–58. 10.1016/S0140-6736(19)32540-1 32085821

[ref-15] Cole TJ : Fitting Smoothed Centile Curves to Reference Data (with Discussion). *J R Stat Soc Ser A.* 1988;151(3):385–418. 10.2307/2982992

[ref-16] Cole TJ Green PJ : Smoothing Reference Centile Curves: The LMS Method and Penalized Likelihood. *Stat Med.* 1992;11(10):1305–19. 10.1002/sim.4780111005 1518992

[ref-17] Coombs CH : A Theory of Data.New York: Wiley,1964. Reference Source

[ref-18] Ellingsen KM : Standardized Assessment of Cognitive Development: Instruments and Issues.In *Early Childhood Assessment in School and Clinical Child Psychology.*edited by E. Garro, Springer,2016;25–49. 10.1007/978-1-4939-6349-2_2

[ref-19] Embretsen SE Reise SP : Item Response Theory for Psychologists.Mahwah, NJ: Lawrence Erlbaum,2000. Reference Source

[ref-20] Engelhard G Jr : Invariant Measurement.New York: Routledge,2013. Reference Source

[ref-21] Engle PL Fernald LCH Alderman H : Strategies for Reducing Inequalities and Improving Developmental Outcomes for Young Children in Low-Income and Middle-Income Countries. *Lancet.* 2011;378(9799):1339–53. 10.1016/S0140-6736(11)60889-1 21944378

[ref-22] Erikson EH : Childhood and Society. 2d Ed., Rev. And Enl.New York, NJ: Norton,1963. Reference Source

[ref-23] Fernald LCH Prado E Kariger P : A Toolkit for Measuring Early Childhood Development in Low and Middle-Income Countries.2017. Reference Source

[ref-24] Frankenburg WK Dodds J Archer P : The Denver II: A Major Revision and Restandardization of the Denver Developmental Screening Test. *Pediatrics.* 1992;89(1):91–97. 1370185

[ref-25] Gesell A : Infant and Child in the Culture of Today.Los Angeles, CA: Read Book Ltd,1943. Reference Source

[ref-26] Grantham‐McGregor SM Fernald LCH Kagawa RMC : Effects of Integrated Child Development and Nutrition Interventions on Child Development and Nutritional Status. *Ann N Y Acad Sci.* 2014;1308(1):11–32. 10.1111/nyas.12284 24673166

[ref-27] Guttman L : The Basis for Scalogram Snalysis.In *Measurement and Prediction. *edited by S. A. Stouffer, L. Guttman, E. A. Suchman, P. F. Lazarsfeld, S. A. Star, and J. A. Clausen, Princeton, NJ: Princeton University Press,1950;IV:60–90.

[ref-28] Hafkamp-de Groen E Dusseldorp E Boere-Boonekamp MM : Relatie Tussen Het van Wiechenonderzoek (d-Score) Op 2 Jaar En Het Intelligentieniveau Op 5 Jaar. [relation Between the Dutch Development Instrument at the Age of 2 Years and Intelligence at the Age of 5 Years]. *Tijdschrift Voor Jeugdgezondheidszorg.* 2009;41(1):10–13. Reference Source

[ref-29] Hattie J : Methodology Review: Assessing Unidimensionality of Tests and ltenls. *Appl Psychol Meas.* 1985;9(2):139–64. 10.1177/014662168500900204

[ref-30] Herngreen WP van Buuren S van Wieringen JC : Growth in Length and Weight from Birth to 2 Years of a Representative Sample of Netherlands Children (born in 1988-89) Related to SocioEconomic Status and Other Background Characteristics. *Ann Hum Biol.* 1994;21(5):449–63. 10.1080/03014469400003472 7985994

[ref-31] Holland PW Wainer H : Differential Item Functioning.Hillsdale, NJ: Lawrence Erlbaum Associates,1983.

[ref-32] Horridge KA : Assessment and Investigation of the Child with Disordered Development. *Arch Dis Child Educ Pract Ed.* 2011;96(1):9–20. 10.1136/adc.2009.182436 20926624

[ref-33] Jacobusse G van Buuren S : Computerized Adaptive Testing for Measuring Development of Young Children. *Stat Med.* 2007;26(13):2629–38. 10.1002/sim.2753 17133649

[ref-34] Jacobusse G van Buuren S Verkerk PH : An Interval Scale for Development of Children Aged 0-2 Years. *Stat Med.* 2006;25(13):2272–83. 10.1002/sim.2351 16143995

[ref-35] Johnson SB Riley AW Granger DA : The Science of Early Life Toxic Stress for Pediatric Practice and Advocacy. *Pediatrics.* 2013;131(2):319–27. 10.1542/peds.2012-0469 23339224 PMC4074672

[ref-36] Kohlberg L : The Psychology of Moral Development: The Nature and Validity of Moral Stages.San Francisco: Harper & Row,1984;2. Reference Source

[ref-37] Kolb B Harker A Gibb R : Principles of Plasticity in the Developing Brain. *Dev Med Child Neurol.* 2017;59(12):1218–23. 10.1111/dmcn.13546 28901550

[ref-38] Liebert RM Poulos RW Strauss GD : Developmental Psychology.Englewood Cliffs, NJ: Prentice-Hall, Inc.1974. Reference Source

[ref-39] Linacre JM : Rasch Model Estimation: Further Topics. *J Appl Meas.* 2004;5(1):95–110. 14757994

[ref-40] Lu C Black MM Richter LM : Risk of Poor Development in Young Children in Low-Income and Middle-Income Countries: An Estimation and Analysis at the Global, Regional, and Country Level. *Lancet Glob Health.* 2016;4(12):e916–22. 10.1016/S2214-109X(16)30266-2 27717632 PMC5881401

[ref-41] Miller AC Murray MB Thomson DR : How Consistent Are Associations Between Stunting and Child Development? Evidence from a Meta-Analysis of Associations Between Stunting and Multidimensional Child Development in Fifteen Low- and Middle-Income Countries. *Public Health Nutr.* 2016;19(8):1339–47. 10.1017/S136898001500227X 26355426 PMC10270805

[ref-42] Mokken RJ : A Theory and Procedure of Scale Analysis: With Applications in Political Research.Berlin: Walter de Gruyter,1971. 10.1515/9783110813203

[ref-43] Molenaar IW : Nonparametric Models for Polytomous Responses.In *Handbook of Modern Item Response Theory.*Springer,1997;369–80. 10.1007/978-1-4757-2691-6_21

[ref-44] Perkins JM Kim R Krishna A : Understanding the Association Between Stunting and Child Development in Low- and Middle-Income Countries: Next Steps for Research and Intervention. *Soc Sci Med.* 2017;193:101–9. 10.1016/j.socscimed.2017.09.039 29028557

[ref-45] Piaget J Inhelder B : The Psychology of the Child.New York, NJ: Basic Books,1969. Reference Source

[ref-46] Rasch G : Probabilistic Models for Some Intelligence and Attainment Tests.Copenhagen: Danish Institute for Educational Research.1960. Reference Source

[ref-47] Rasch G : On Specific Objectivity: An Attempt at Formalizing the Request for Generality and Validity of Scientific Statements. *The Danish Yearbook of Philosophy.* 1977;14:58–93. Reference Source

[ref-48] Robitzsch A : Sirt: Supplementary Item Response Theory Models.2016. Reference Source

[ref-49] Rutter M : Genes and Behavior: Nature-Nurture Interplay Explained.Hogrefe Publishing,2007.

[ref-50] Salkind NJ : Child Development.Macmillan Library Reference,2002. Reference Source

[ref-51] Santrock JW : Child Development: An Introduction. 13th Ed.New York, NJ: McGraw-Hill Higher Education,2011. Reference Source

[ref-52] Shirley MM : The First Two Years: A Study of Twenty-Five Babies. Vol. I: Postural and Locomotor Development.Minneapolis: University of Minnesota Press,1931. Reference Source

[ref-53] Shirley MM : The First Two Years: A Study of Twenty-Five Babies. Vol. II: Intellectual Development.Minneapolis: University of Minnesota Press,1933.

[ref-54] Shonkhoff JP Levitt P Fox NA : From Best Practices to Breakthrough Impacts: A Science-Based Approach to Building a More Promising Future for Young Children and Families.Harvard University, Center on the Developing Child Cambridge, MA.2016. Reference Source

[ref-55] Stasinopoulos DM Rigby RA : Generalized Additive Models for Location Scale and Shape (GAMLSS) in r. *J Stat Softw.* 2008;23(7):1–46. 10.18637/jss.v023.i07

[ref-56] Stott LH : Child Development: An Individual Longitudinal Approach.New York, NJ: Holt, Rinehart; Winston, Inc.1967. 10.1002/1520-6807(196801)5:1<92::AID-PITS2310050120>3.0.CO;2-D

[ref-57] Sudfeld CR McCoy DC Danaei G : Linear Growth and Child Development in Low- and Middle-Income Countries: A Meta-Analysis. *Pediatrics.* 2015;135(5):e1266–75. 10.1542/peds.2014-3111 25847806

[ref-58] UNICEF: The State of the World’s Children 2019: Children, Food and Nutrition: Growing Well in a Changing World. *UNICEF.* 2019. Reference Source

[ref-59] van Buuren S : Growth Charts of Human Development. *Stat Methods Med Res.* 2014;23(4):346–68. 10.1177/0962280212473300 23487019

[ref-60] van Buuren S : D-score/childdevdata: childdevdata 1.0.1. (Version v1.0.1). *Zenodo.* 2021. 10.5281/zenodo.4685979

[ref-61] van Buuren S Eekhout I : Child development with the D-score: tuning instruments to unity. *F1000Res.* (in press).2021. Reference Source 10.12688/gatesopenres.13222.2 PMC11813173 39935809

[ref-62] Verloove-Vanhorick SP Verwey RA Brand R : Neonatal Mortality Risk in Relation to Gestational Age and Birthweight. Results of a National Survey of Preterm and Very-Low-Birthweight Infants in the Netherlands. *Lancet.* 1986;1(8472):55–57. 10.1016/s0140-6736(86)90713-0 2867312

[ref-63] Vlasblom E Boere-Boonekamp MM Hafkamp-de Groen E : Predictive Validity of Developmental Milestones for Detecting Limited Intellectual Functioning. *PLoS One.* 2019;14(3):e0214475. 10.1371/journal.pone.0214475 30921424 PMC6438572

[ref-64] Wainer H Dorans NJ Flaugher R : Computerized Adaptive Testing: A Primer.Routledge,2000. Reference Source

[ref-65] Weber AM Rubio-Codina M Walker SP : The D-score: A Metric for Interpreting the Early Development of Infants and Toddlers Across Global Settings. *BMJ Glob Health.* 2019;4(6):e001724. 10.1136/bmjgh-2019-001724 31803508 PMC6882553

[ref-66] Wit JM Himes JH van Buuren S : Practical Application of Linear Growth Measurements in Clinical Research in Low- and Middle-Income Countries. *Horm Res Paediatr.* 2017;88(1):79–90. 10.1159/000456007 28196362 PMC5804842

[ref-67] Wright BD Masters GN : Rating Scale Analysis: Rasch Measurement.Chicago: MESA Press,1982. Reference Source

[ref-68] Zumbo BD : A Handbook on the Theory and Methods of Differential Item Functioning (DIF). *Ottawa: National Defense Headquarters.* 1999. Reference Source

[ref-69] Zwinderman AH : Pairwise Parameter Estimation in Rasch Models. *Appl Psychol Meas.* 1995;19(4):369–75. 10.1177/014662169501900406

