# Supplementary material for: Child development with the D-score: turning milestones into measurement
Source: Gates Open Res. 2022 Dec 20;5:81. Originally published 2021 May 13. [Version 2] doi: 10.12688/gatesopenres.13222.2 (PMC11813173; doi:10.12688/gatesopenres.13222.2)
Supplement: Peer review history

# Reviewer report: Reviewer response for version 2

**DOI:** 10.21956/gatesopenres.15140.r38861

**Published:** 2025-01-22

**Authors:** James Heckman

**Affiliations:** 1 University of Chicago, Chicago, USA

**Review of “Child Development with the D-Score: Turning Milestones into Measurement”**

 I. The goal of this study is enormously ambitious and perhaps impossible. It seeks a universal scale of child development across multiple domains of human skills. A more focused approach based on well-defined domains of skill would be far more credible, especially if anchors (common measures) could be found that were the same across different studies. In my own work on measuring development of young children, the trajectories by age for gross motor skills, language, fine motor skills and cognition can take very different paths for the same child. Forcing a single trajectory across very different skills will only produce an uninterpretable mélange. This is evident in my own work on rural Chinese skill development.

**Problem P1. Lack of any Convincing Rationale**

 The authors haven't completely taken into account the multiplicity of skills well-documented for human beings, including young children, for whom skills develop at different rates. The authors approach the topic as a purely statistical exercise based on axioms they like but that are not motivated by subject matter expertise. This is an exercise in pure statistics and not science. It is not justified by the science of human development.

 II. The intended audience is not at all clear to me. Is it just clients of the Gates Foundation who will be required to construct D-scores? Is it intended as a cookbook for a compliant audience?  Many crucial steps are missing that will require the reader to read statistics journals of varying mathematical requirements and notation.

 A good cookbook with many illustrative examples combining multiple measurements and panel and cross-section data would be much more useful. Applied statisticians could imitate the steps in the cookbook, and the cookbook would contain ways to analyze a variety of data from different samples.

 The authors claim to have a general methodology, but in the examples given, they show otherwise. Consider their example where they throw out height growth curve analysis because it is too age-related! There are other examples.

 III. The exposition is unclear. D-scores are discussed before they are defined. Reference is made to EAP, which is a standard tool, but the EAP scores are for a single person using multiple assessments (Bock and Mislevy 1982 [Ref-1]). But as I understand it, the authors want to compute D-scores on samples of populations--including cross sections--using empirical Bayes methods. If so, the authors need to tell readers how to construct the prior on cross sections and longitudinal files on heterogenous populations. It is very unclear how the authors propose to adjust for *β* *_n_* ( ***x***), where ***x*** is a vector of agent characteristics.

**Problem P2. How to Construct the Prior for D Using Multiple Datasets Including Cross-Sections the Authors Claim They Can Use.**

 IV. Interpretively, *β* *_n_* ( ***x***) is called “ability.” It is assumed fixed. “Learning” occurs as the thresholds facing agents become easier. In my own version of this framework (see Heckman and Zhou 2025 and many other papers), ability grows with instruction, so β _n,E_ = β (E, θ) + ε _n,E_, where “ *E*” is exposure or education, *θ* is true ability and

*ε _n,E_* are shocks. Their model has aspects of the Raasch model because growth in β _n _– δ (a)  can be interpreted as growth in β (E _a_, θ) which is equivalent to thresholds ( *δ*) decreasing. However, an interesting property of the Raasch model, that *β* *_n_* is fixed, is lost and with it the conditional property they use. *β* *_n_* – *δ*( *a*) is an awkward way to describe growth in ability and learning. Thresholds decrease with age, but ability is constant. A more interpretable model of ability growth consistent with learning would extend the Raasch model in the fashion of Heckman and Zhou (2025).

 V. The paper does not provide proper justifications for many of its claims. It fails to be convincing as an intellectual document. The Raasch model is taken as obviously correct. It fails to capture learning. It is a model of assessment of a fixed ability. Numerous econometric studies of skill growth using it reject it as a good description of economic threshold-crossing models. The local independence assumed (conditional on *β _n_*) is rejected in many panel settings, including some I have personally fit. Many models of learning depend on a person’s characteristics and characteristics of schools and institutions. Learning about *β _n_* often induces martingale dependence and not the conditional independence given a scalar assumed in this study. The univariate skill assumption has been rejected in models of learning and child development (see, e.g., Zhou, Heckman, Liu and Lu 2025 [Ref-3] and other papers).

 There is no attempt to provide a sound, commonsense defense of use of a scalar measure for unobservables. Indeed, there is no mention or use of a class of tests for that hypothesis by Holland and Rosenbaum (1986 [Ref-2]). Phrases like “their approach is to reject data that don’t fit with their model” are rather bizarre and unlikely to convince any audience other than a captive one. Throughout there are various “fix ups” suggested when empirical problems arise, but these are not guided by any general principles. They do not inspire confidence. There is no agreement that the Englehardt axioms are proper requirements for a model of learning or measurement. To this reader, their invocation was an empty appeal to authority.

**Problem P3. Many Important Details and Justifications are Lacking**

 I could go on in much greater detail, but I have written enough. There is no universal scale on which to measure human development. What would the D-scores be of Albert Einstein and Muhammad Ali? How to weight muscle mass and coordination against intellectual prowess that (in Einstein’s case) developed slowly? The exposition is disorderly. Terms are used before they are defined. Crucial issues like how to define the empirical Bayes prior in the general case are missing. The theory of skill development underlying this approach is at odds with a rich new theory of skill dynamics with emergent skills by age and stage of the life cycle.

If applicable, are any methods and analyses appropriate and sufficiently detailed, to allow replication by others?

Yes

Are the conclusions drawn appropriate and justified on the basis of the presented work?

No

Are any opinions stated well-argued, clear, and cogent?

Partly

Are all factual statements correct and adequately supported by citations to the current literature?

Partly

If applicable, are the source data underlying the results available to ensure full reproducibility?

Partly

Is the work clearly and accurately presented?

Partly

Is the work an appropriate addition to this book project, and does it align with the scope and/or objectives?

Partly

Reviewer Expertise:

Econometrics, labor economics, human capital

I confirm that I have read this submission and believe that I have an appropriate level of expertise to state that I do not consider it to be of an acceptable scientific standard, for reasons outlined above.

## References

[1] : Adaptive EAP Estimation of Ability in a Microcomputer Environment. *Applied Psychological Measurement* .1982;6(4) : 10.1177/014662168200600405 431-444 10.1177/014662168200600405

[2] : Conditional Association and Unidimensionality in Monotone Latent Variable Models. *The Annals of Statistics* .1986;14(4) : 10.1214/aos/1176350174 10.1214/aos/1176350174

[3] : The Impact of a Prototypical Home Visiting Program on Child Skills. *Journal of Labor Economics* .2024; 10.1086/732301 10.1086/732301 PMC1296274441799424

[4] : Measuring Knowledge and Learning.2022; 10.3386/w29990 10.3386/w29990

# Reviewer report: Reviewer response for version 2

**DOI:** 10.21956/gatesopenres.15140.r38748

**Published:** 2025-01-06

**Authors:** Nirmala Rao, Zeyi Li

**Affiliations:** 1 Education, The University of Hong Kong, Hong Kong, Hong Kong; 2 The University of Hong Kong, Hong Kong, China

**Child development with the D-score: Turning milestones into measurement**

**Reviewed by Zeyi Li and Nirmala Rao**

 Thank you for inviting us to review this chapter, which has clearly benefitted from earlier reviews. The authors have been very responsive to previous feedback. We first provide some general comments before addressing specific sections of the chapter.

 Overall, the chapter provides an informative introduction to the D-score method. The authors note that it is written for professionals in child growth and development, policymakers in international settings, and statisticians, methodologists, and data scientists. Although the authors try to simplify the concepts, we believe this chapter may be too advanced for child growth and development professionals and policymakers in international settings.

 There are several notable and commendable aspects of the chapter. First, the authors illustrate the rationale and application of the D-score method using authentic datasets. The authors focus on the first 80 weeks of life and mention the first 1000 days in Section 1.1. A more accurate title for the chapter would be “Child Development with the D-score in the First 2 Years: Turning Milestones into Measurement.” Given our common biology, there are remarkable similarities in achieving early developmental milestones across cultures. The maturation of the brain and nervous system follows a similar timeline globally, leading to common milestones such as sitting, walking, and talking. Further, infants and toddlers have similar basic needs for social interaction, which are met in similar ways across different cultures. This results in comparable developmental outcomes. This is why we see similar developmental outcomes in Togo and the Netherlands (although children from Togo appear more advanced than those from the Netherlands). This cross-cultural similarity is due to the predominance of motor items (see Figure 7.3), and this issue should be addressed in detail in the discussion.

**Section 1.2:** There are various reasons why monitoring children’s development at an early age is important. In the chapter, the authors particularly emphasise screening for potential developmental delays. The authors should distinguish between screening at the population level and individual assessment for diagnostic purposes.

**Section 1.4:** Different assessment tools will undoubtedly have strengths and limitations as they are developed for different populations and purposes. A stronger rationale should be presented for using the D-score across different countries in children above 2 years, as motor milestones would be achieved by then.

**Section 4.5.1:** Since development occurs across developmental domains, is it appropriate to assume unidimensionality when measuring early childhood development? Specifically, is there a unified latent variable representing children’s overall development using the IRT model? We should also be cautious regarding the assumption of local independence. Sometimes, in direct assessment, a child’s response to an item does not necessarily reflect their ability; other factors such as unfamiliarity, reluctance, or shyness may also influence their performance on an assessment task.

**Section 6.3.1:** Can the authors elaborate further on the sentence: “DIF is undesirable since it can make the instrument culturally biased”? I wonder about the authors’ thoughts on balancing the need for a tool to be culturally comparable and culturally sensitive. Sometimes, DIF can help us detect cultural differences in children’s development. Other times, it may indicate differences in development based on children’s socioeconomic status, residence, or gender, rather than cultural differences per se.

**Section 7.2.2:** The authors may consider addressing criterion validity, such as the relationships between D-scores and child age, urbanicity, and SES. The statement “Thus, growth and development are entirely different concepts” is not accurate as the authors do not specify what milestones are measured by D-scores. Growth could correlate more with D-scores if we only look at motor development, for example. Therefore, the statement may differ based on the specific domains we assess.

**Section 9.2 POPS study:** The authors state, “Since the D-scores are calculated using the same milestones and difficulty estimates as used in the SMOCC data, the D-scores are comparable across the two studies.” Does this mean that the D-scores for the POPS study were calculated using difficulty estimates derived from children’s responses in the SMOCC data? If so, how can we ensure that the difficulty estimates from the SMOCC data apply to the sample in the POPS study, given that these are two different populations?

**Section 10.5:** The authors highlight the importance of the same milestones between assessment instruments to calculate D-scores. Can the authors explain what “overlapped milestones” mean? Does it mean only the assessment content is the same, or do both the assessment content and the scorings (e.g., “Yes” or “No”) need to be the same between the two measures? Is there any requirement regarding the implementation or assessment approach? A detailed clarification would be needed here.

If applicable, are any methods and analyses appropriate and sufficiently detailed, to allow replication by others?

Yes

Are the conclusions drawn appropriate and justified on the basis of the presented work?

Partly

Are any opinions stated well-argued, clear, and cogent?

Yes

Are all factual statements correct and adequately supported by citations to the current literature?

Partly

If applicable, are the source data underlying the results available to ensure full reproducibility?

Yes

Is the work clearly and accurately presented?

Yes

Is the work an appropriate addition to this book project, and does it align with the scope and/or objectives?

Yes

Reviewer Expertise:

Early Childhood Development and Education

We confirm that we have read this submission and believe that we have an appropriate level of expertise to confirm that it is of an acceptable scientific standard.

# Reviewer report: Reviewer response for version 2

**DOI:** 10.21956/gatesopenres.15140.r35745

**Published:** 2024-02-09

**Authors:** Patricia Kitsao-Wekulo

**Affiliations:** 1 African Population and Health Research Center, Nairobi, Nairobi County, Kenya

The paper provides information on the development of the D-score which could provide a common yardstick on how to measure children's development across different contexts. This has important implications for research, policy, and practice in early child development, especially when considering that it is critical to identify those who require early intervention. 

 General comments:

 1. The paper needs to be checked thoroughly  for typographical and grammatical errors

 2. The authors should be consistent in applying the use of US or UK English

 3. There are misplaced brackets which should be corrected

 4. Shortened words should be written out in full, e.g. instead of 'won't,' please use 'will not'

 Specific comments:

 Abstract:

 The bullet points in the abstract should be presented consistently, that is, all items should begin with the same part of speech.

 Introduction: Sub-heading 1.1. could be modified to 'The first 1000 days.' The first paragraph of the introduction is quite repetitive and could be made more concise. For instance, the word 'architecture' appears 3 times. The entire sub-section 1.1 should be rewritten to minimize repetition and improve clarity.

 Parts of section 1.2 repeat some of the information already presented in section 1.1.

 1.3 - The sub-heading could be amended to 'Stunting as a proxy for child development. Check this statement 'Stunting caused by chronic nutritional deprivation in early childhood is an indicator of child development'

 1.4 - 2017 is not very recent. This is in reference to the statement on the tool review by Fernald and colleagues.

 Short history: There is material on development as a continuous process that has been repeated under sub-sections 2..1 and 2.2.1. The authors should make a decision on where this material is best placed.  

 2.3.1 - The first paragraph indicates that 25 babies were included in Shirley's study. The second paragraph mentions 21 babies. Please indicate why the data of the 25 babies are not provided. Also, were these anonymized data?

 The presentation of the information provided in Table 2.1 and Figure 2.3 is repetitious

 The D-score: This section is the gist of the paper. The sections before it are quite lengthy and the information could be provided more succinctly

 The sub-sections under this section are quite academic. I would prefer to see a more critical analysis of the outcomes of the different studies that have been discussed here. In particular, attempts should be made to show how the use of the D-score could be applied more specifically to studies included in the paper.

 Some of the statements presented in the paper are not adequately supported by literature, or the citations used are quite dated. The conclusion seems to cover only some aspects of the paper and should be made more comprehensive.

If applicable, are any methods and analyses appropriate and sufficiently detailed, to allow replication by others?

Not applicable

Are the conclusions drawn appropriate and justified on the basis of the presented work?

Partly

Are any opinions stated well-argued, clear, and cogent?

Partly

Are all factual statements correct and adequately supported by citations to the current literature?

Partly

If applicable, are the source data underlying the results available to ensure full reproducibility?

Not applicable

Is the work clearly and accurately presented?

Partly

Is the work an appropriate addition to this book project, and does it align with the scope and/or objectives?

Yes

Reviewer Expertise:

Early childhood develpment

I confirm that I have read this submission and believe that I have an appropriate level of expertise to confirm that it is of an acceptable scientific standard, however I have significant reservations, as outlined above.

# Reviewer report: Reviewer response for version 1

**DOI:** 10.21956/gatesopenres.14445.r30848

**Published:** 2021-07-08

**Authors:** Alexander Robitzsch

**Affiliations:** 1 IPN—Leibniz Institute for Science and Mathematics Education, Kiel, Germany

This book chapter is a comprehensive and very informative treatment of the D-score method. I am convinced that the D-score method is beneficial in many applications. I am reserved about some of the promises of the method. I will comment on this in detail in the following:

  

1. The running example is the ”gross-motor functioning” milestone. It seems that this is a polytomous item with five stages (score 0 to 4). However, the stages seem to be handled as separate items in the analysis. Ignoring this kind of local dependence will typically result in overestimated reliabilities and biased item difficulties. It would be interesting why the authors followed their approach.
2. In many parts of the chapter, it is claimed that the D-score provides measurements on an interval scale. I think that the concept of scales in representational measurement theory (RMT) is a concept that does (and should) not have meaning in empirical science. In contrast, I think that particular mathematical operations can be ”admissible” (in the sense of RMT) in particular analyses. Hence, the scale level is not coupled on a variable (or a scale) but using a variable in analysis (see work of Reinhard Niederee).
3. Notably, it has been pointed out by several scholars that more complicated item response theory (IRT) models like the two-parameter logistic (2PL) IRT model can also have ”interval scale” properties by considering a well-defined system of functional equations (e.g., Ballou, 2009 ^1^). Hence, the arguments of why using the Rasch model (RM) as the basis for the D-score method are flawed. Other (more complicated) IRT models can also be used. In my opinion, the only reason for using the RM might be that the sum score is a sufficient statistic. Hence, items are equally weighed in the scale score, which might be considered a desirable property.
4. P. 18: I suspect that many of the biased statements of the authors about IRT models come from a selective reading of the literature. While ”Embretson & Reise, Wright & Masters and Engelhard” could be classified as ”good introductory books” (I would disagree with such a statement), these transport many of the myths of the RM compared to more complex IRT models. The myths surround the concepts of ”sample-free measurement”, ”specific objectivity” and ”invariant measurement” (Engelhard). After appropriate identification constraints of a latent variable, there is no difference concerning these concepts for the RM and the 2PL model. The 2PL model also provides sample-free measurements as the RM because the invariance property can hold for any IRT model. The invariance property is based on the local independence assumption and the absence of differential item functioning (DIF) with respect to age. Moreover, specific objectivity is usually equated with the concept of parameter separability for persons and items, which can also be achieved for the 2PL model (Irtel, Ballou). I do not ask authors to adopt the D-score to the 2PL or other alternative IRT models, but to remove their flawed statements about the ”unique properties” of the RM.
5. Authors should be more careful with the statement that cross-cultural DIF implies biased measurement. There is rich literature arguing that the concepts of DIF and bias (or fairness) should not be equated (Camilli), and a DIF item is important for assessing differences between countries (or cultures). Hence, just removing DIF items is a misguided recommendation.
6. In the same vein, removing items from the scale that does not fit the RM might threaten validity. This should be emphasized in the section ”validity” which is silent about this issue. I would rather see the RM as a device for linking items using equal weights for items. Typically, the RM is misspecified; but why bother about it?
7. The authors propose the pairwise estimation approach (PAIR) of Zwinderman. If I read the chapter correctly, items are (partly) administered to children depending on their (expected) level of ability. This means that items are missing at random (MAR). PAIR estimation does not work for (general) MAR data but provides consistent estimates under MCAR. It might be that a particular missingness design does not lead to biased estimates in the PAIR method, but authors should elaborate on why preferring this estimation approach over likelihood-based estimation approaches.
8. See above. I do not think that the information function is a valuable concept for assessing the uncertainty of the D-score. First, local dependence seems to be ignored. Second, it presupposes a correctly specified measurement model, which is certainly violated. Resampling item (groups) is a much better alternative for quantifying standard errors.

Is the book chapter original and does it add to the body of knowledge?

Yes

If applicable, are any methods and analyses appropriate and sufficiently detailed, to allow replication by others?

Partly

Are the conclusions drawn appropriate and justified on the basis of the presented work?

Partly

Is the book chapter applicable to the community that it is targeting, and, if applicable, does it include practical or social implications?

Yes

Are any opinions stated well-argued, clear, and cogent?

Partly

Are all factual statements correct and adequately supported by citations to the current literature?

Yes

If applicable, are the source data underlying the results available to ensure full reproducibility?

Not applicable

Is there a clear aim of the book chapter, and is it placed in the context of previous work?

Yes

Is the work clearly and accurately presented?

Yes

Is the work an appropriate addition to this book project, and does it align with the scope and/or objectives?

Partly

Reviewer Expertise:

NA

I confirm that I have read this submission and believe that I have an appropriate level of expertise to confirm that it is of an acceptable scientific standard, however I have significant reservations, as outlined above.

## References

[1] : Test Scaling and Value-Added Measurement. *Education Finance and Policy* .2009;4(4) : 10.1162/edfp.2009.4.4.351 351-383 10.1162/edfp.2009.4.4.351

# Response

**Published:** 2022-09-23

**Authors:** Stef van Buuren

**Response to Alexander Robitzsch**

 Stef van Buuren, Iris Eekhout

*This book chapter is a comprehensive and very informative treatment of the D-score method. I am convinced that the D-score method is beneficial in many applications. I am reserved about some of the promises of the method. I will comment on this in detail in the following:*

 We thank Alexander Robitzsch for his thoughtful comments. Robitzsch mentions a number of methodological issues. We considered most of these during our writing, but opted to leave out method discussions that we felt were out of scope for the intended audience. Nevertheless, those problems are relevant, so here we take the opportunity to highlight some of our thoughts.

*1. The running example is the ”gross-motor functioning” milestone. It seems that this is a polytomous item with five stages (score 0 to 4). However, the stages seem to be handled as separate items in the analysis. Ignoring this kind of local dependence will typically result in overestimated reliabilities and biased item difficulties. It would be interesting why the authors followed their approach.*

 In practice items are administered as **independent binary milestones**, typically a subset of milestones appropriate for age. We have no full assessment of “five stages” at all ages, but an assessment of a priori chosen transition at a particular age. The sequence of development stages is not the same for all children. Children may take different routes (e.g. some children never crawl, but find other ways to propel themselves). In such cases, it is not possible to formulate an item-sequence that would be appropriate for all children. Thus for data collected in this way, the binary model is the appropriate choice.

*2. In many parts of the chapter, it is claimed that the D-score provides measurements on an interval scale. I think that the concept of scales in representational measurement theory (RMT) is a concept that does (and should) not have meaning in empirical science. In contrast, I think that particular mathematical operations can be ”admissible” (in the sense of RMT) in particular analyses. Hence, the scale level is not coupled on a variable (or a scale) but using a variable in analysis (see work of Reinhard Niederee).*

 In practice we wish to interpret the difference between two D-scores (within or between children) as being independent of its location on the scale. According to the Stevens’ classification, a scale with a constant unit (an interval scale) will achieve this. Strictly speaking, we might argue that “interval” should not be a property of the scale. However, the concept of “interval scale” is well accepted and widely used, and its slight misnomer does not seem to have implications for everyday work.

*3. Notably, it has been pointed out by several scholars that more complicated item response theory (IRT) models like the two-parameter logistic (2PL) IRT model can also have ”interval scale” properties by considering a well-defined system of functional equations (e.g., Ballou, 2009 1). Hence, the arguments of why using the Rasch model (RM) as the basis for the D-score method are flawed. Other (more complicated) IRT models can also be used. In my opinion, the only reason for using the RM might be that the sum score is a sufficient statistic. Hence, items are equally weighed in the scale score, which might be considered a desirable property.*

 First of all, having an interval scale is not the only reason for preferring the Rasch model. The Rasch model is a special case of the 2PL model, so the 2PL will always fit the data better. We found that the Rasch model fits the data quite well and hence provides a more parsimonious description of the data than the 2PL. Also, on the danger of sounding zealous, we find interpretation of 2PL models less intuitive. In particular, we cannot say anymore that one milestone is more difficult than another, but should qualify that statement by the ability level. We find that hard to explain to practitioners. We were not aware of the Ballou 2009 paper. The model may have its merits in particular cases. For our application we feel it is more complex than we need.

*4. P. 18: I suspect that many of the biased statements of the authors about IRT models come from a selective reading of the literature. While ”Embretson & Reise, Wright & Masters and Engelhard” could be classified as ”good introductory books” (I would disagree with such a statement), these transport many of the myths of the RM compared to more complex IRT models. The myths surround the concepts of ”sample-free measurement”, ”specific objectivity” and ”invariant measurement” (Engelhard). After appropriate identification constraints of a latent variable, there is no difference concerning these concepts for the RM and the 2PL model. The 2PL model also provides sample-free measurements as the RM because the invariance property can hold for any IRT model. The invariance property is based on the local independence assumption and the absence of differential item functioning (DIF) with respect to age. Moreover, specific objectivity is usually equated with the concept of parameter separability for persons and items, which can also be achieved for the 2PL model (Irtel, Ballou). I do not ask authors to adopt the D-score to the 2PL or other alternative IRT models, but to remove their flawed statements about the ”unique properties” of the RM.*

 Sample-free measurement is indeed crucial because our samples have disparate health distributions. We were not aware of the Ballou paper, and of the circumstances under 2PL models can also be sample-free. We added:

 “Note that the Rasch model is not unique in all aspects. A reviewer indicated that specific objectivity and invariant measurement might also be achieved in certain 2PL models. For us, the combination of simplicity, interpretability and convenient properties makes the Rasch model stand out.

*5. Authors should be more careful with the statement that cross-cultural DIF implies biased measurement. There is rich literature arguing that the concepts of DIF and bias (or fairness) should not be equated (Camilli), and a DIF item is important for assessing differences between countries (or cultures). Hence, just removing DIF items is a misguided recommendation.*

 We agree that we need to be careful in wording. Our statement was “DIF is undesirable since it can make the instrument culturally biased”. Thus, we say that DIF (biased measurement) can lead to unfair cross-cultural comparison (not the other way around). In our conception, DIF of an item means: **at same ability level** the probabilities of endorsing differ. In general, we consider DIF as undesirable.

*6. In the same vein, removing items from the scale that does not fit the RM might threaten validity. This should be emphasized in the section ”validity” which is silent about this issue. I would rather see the RM as a device for linking items using equal weights for items. Typically, the RM is misspecified; but why bother about it?*

 It is possible that item removal lowers content validity. Our section 6.1 (on item fit) emphasizes that the availability of sufficient number of alternative items should be a factor in considering removal of an item. Of course, removing a “bad” item (e.g. that does not assess development, but some other trait) may increase construct validity. We have added to the validity section:

 “Content validity assessment is part of modelling when we examine what milestones fit the model. Content validity also means that all relevant facets of development are measured. As discussed in Section 6.1, we may remove items that do not fit the model and hence fail to measure development in the technical sense. As a result, we may lose items considered relevant by subject-matter specialists. If we want to preserve these, we could fit a separate model that captures another development aspect. We did not encounter the issue with the DDI. In contrast, our finding that items allocated to different domains form a unidimensional scale underlines the content validity of the D-score.”

*7. The authors propose the pairwise estimation approach (PAIR) of Zwinderman. If I read the chapter correctly, items are (partly) administered to children depending on their (expected) level of ability. This means that items are missing at random (MAR). PAIR estimation does not work for (general) MAR data but provides consistent estimates under MCAR. It might be that a particular missingness design does not lead to biased estimates in the PAIR method, but authors should elaborate on why preferring this estimation approach over likelihood-based estimation approaches.*

 The data are partially observed since only age-appropriate items are administered. Consequently, all non-administered items have missing values. Since the missing data mechanism is under control of the investigator the data are MAR. The PAIR method provides excellent estimates of item difficulty in this setup. In contrast, likelihood-based estimation approaches do not work and lead to biased estimates because these methods may require unrealistic assumptions on the marginal distribution.

 The simulation script at the end of this document demonstrates that the PAIR method yield correct item difficulty estimates for partially observed data, whereas maximum likelihood does not.

*8. See above. I do not think that the information function is a valuable concept for assessing the uncertainty of the D-score. First, local dependence seems to be ignored. Second, it presupposes a correctly specified measurement model, which is certainly violated. Resampling item (groups) is a much better alternative for quantifying standard errors.*

 We do not employ the information function to assess uncertainty in the D-score. Our preferred way to quantify uncertainty is the variance of the individual D-score posterior, as in Section 5.3. This variance is sensitive to the number and targeting of the items administered, which may vary from individual to individual. The information function in Section 6.4 has a role in targeting items to ages. We were not quite sure how to interpret the suggestion on resampling, in particular whether that method would apply to standard errors for item parameters, for individual D-scores, or for the test as a whole.

 We thank the reviewer for his penetrating comments. These have helped us to improve our work.

 Please find the simulation script (mentioned above) in the file linked here.
